# Potential placebo bias in current trials of delirium prevention: a network meta‐analysis of 86 randomized controlled trials

**DOI:** 10.1111/pcn.13850

**Published:** 2025-06-18

**Authors:** Bing‐Yan Zeng, Chih‐Sung Liang, Chih‐Wei Hsu, Wei‐Te Lei, Trevor Thompson, Yen‐Wen Chen, Tien‐Yu Chen, Ping‐Tao Tseng, Yow‐Ling Shiue

**Affiliations:** ^1^ Institute of Biomedical Sciences National Sun Yat‐sen University Kaohsiung Taiwan; ^2^ Department of Internal Medicine E‐Da Dachang Hospital, I‐Shou University Kaohsiung Taiwan; ^3^ Department of Psychiatry, Beitou Branch, Tri‐Service General Hospital; School of Medicine National Defense Medical Center Taipei Taiwan; ^4^ Department of Psychiatry National Defense Medical Center Taipei Taiwan; ^5^ Department of Psychiatry Kaohsiung Chang Gung Memorial Hospital and Chang Gung University College of Medicine Kaohsiung Taiwan; ^6^ Section of Immunology, Rheumatology, and Allergy Department of Pediatrics Hsinchu Munipical MacKay Children’s Hospital Hsinchu Taiwan; ^7^ Center for Molecular and Clinical Immunology Chang Gung University Taoyuan Taiwan; ^8^ Centre for Chronic Illness and Ageing University of Greenwich London UK; ^9^ Prospect Clinic for Otorhinolaryngology & Neurology Kaohsiung Taiwan; ^10^ Department of Psychiatry Tri‐Service General Hospital; School of Medicine, National Defense Medical Center Taipei Taiwan; ^11^ Institute of Brain Science National Yang Ming Chiao Tung University Taiwan; ^12^ Institute of Precision Medicine National Sun Yat‐sen University Kaohsiung Taiwan

**Keywords:** delirium, network meta‐analysis, placebo effect, prevention, prophylaxis

## Abstract

**Aim:**

Numerous network meta‐analyses (NMAs) have reported inconsistent findings on the efficacy of various pharmacologic treatments for delirium prevention in high‐risk patients. A potential confounder was the study design—using a placebo control or standard‐of‐care (SoC) control. We reexamined the incident delirium between SoC plus placebo and SoC alone in randomized controlled trials (RCTs) on delirium prevention.

**Methods:**

We systematically searched for relevant RCTs on pharmacotherapies for delirium prevention from the inception of electronic databases through 30 November 2023. The primary outcome was delirium incidence, with secondary consideration given to all‐cause mortality. We conducted a frequentist NMA using risk ratios (RRs) with 95% confidence intervals (CIs) as the effect size.

**Results:**

The NMA results from 86 RCTs (19,889 participants, with a mean age of 68.5 years, and a mean female proportion of 44.0%) revealed that SoC plus placebo was associated with a lower risk of incident delirium than SoC alone (RR, 0.60 [95% CI, 0.41–0.88]). The RR of all‐cause mortality was not significant between these two study designs.

**Conclusion:**

We found placebo effects on the incidence of delirium in RCTs involving high‐risk patients. Further RCTs should consider a three‐arm, parallel design including both a placebo group and an SoC group to replicate our study findings.

**Trial Registration:**

CRD42023488481.

Delirium represents a significant complication in critically ill patients, demonstrating 3.2 to 6.5 times higher mortality and in‐hospital complications compared with controls.^1^ Delirium is also associated with a heightened risk of falls, functional decline, permanent cognitive impairment (e.g. dementia), prolonged hospitalization, and eventual institutionalization.^2^ Several large‐scale meta‐analyses and network meta‐analyses (NMAs) using data from randomized controlled trials (RCTs) have examined pharmacotherapies for delirium prevention in high‐risk patients.^3^, ^4^, ^5^, ^6^ However, the results were inconsistent. On inspecting the included RCTs in the aforementioned NMAs, we observed a trend suggesting potentially lower incident delirium in RCTs with placebo control than those RCTs with standard of care (SoC) alone.^3^, ^4^, ^5^, ^6^

The application of a placebo arm in RCTs has been a subject of debate,^7^ although a placebo arm might show placebo effects on behavioral changes.^8^ Placebo or nocebo effects are usually assumed to play a role in only subjective or minor outcomes, such as pain^9^ or cough^10^; however, accumulating evidence suggests its potential impact on major outcomes of several critical diseases. For instance, in a systematic review of cardiovascular studies utilizing all‐cause mortality as the outcome,^11^ the placebo‐controlled arm of a study on peripheral arterial disease showed nocebo effects on mortality rate compared with the average mortality in the general peripheral arterial disease population.^12^ From that review, the placebo‐related behavioral change might be one of the key causes of the biased outcome of all‐cause mortality. Similarly, in another recent important study, the authors noticed that application of placebo in a statin trial (i.e. a medication to lower serum lipids to reduce cardiovascular risk) might result in the false belief that ‘he or she can eat anything and stop exercising’ so that it might lead to a worse outcome than the waiting list.^13^

While few RCTs have directly examined the impact of placebo application on major outcomes, some statistical evidence from NMAs has been presented in RCTs related to all‐cause mortality in COVID‐19 treatment. In a recent large‐scale NMA,^14^ significant differences in all‐cause mortality were found between patients receiving SoC plus placebo and those in the control group (i.e. SoC alone) in COVID‐19 treatment RCTs. To be specific, in RCTs of interleukin 6 antagonists, patients who received SoC plus placebo showed a statistically lower rate of all‐cause mortality than the control group (i.e. SoC alone).^14^ Despite ongoing discussions about the impact of placebo use in minor diseases or subjective outcomes,^15^ our previous NMA revealed differences in an objective outcome (i.e. all‐cause mortality) in critical diseases (i.e. moderate to severe COVID‐19 infection) between SoC plus placebo and the control group (i.e. SoC alone). This difference might result from placebo‐related behavioral change. For example, in a previous meta‐analysis, good adherence to placebo was associated with lower mortality compared with poor adherence.^16^

Based on our previous work,^3^ if there is a placebo effect in delirium prevention, it may lead to an overestimation of the efficacy of the compared treatment. Conversely, if a nocebo effect is present, it could result in an underestimation of the effectiveness. Therefore, it is important to conduct a comprehensive reinvestigation of this life‐critical treatment for delirium prevention. The current NMA aims to reexamine whether patients at high risk of delirium development, treated with SoC plus placebo, exhibit a different incidence of delirium compared with those in the control group (i.e. SoC alone).

## Methods

The present investigation adhered to the guidelines outlined in the PRISMA (Preferred Reporting Items for Systematic Reviews and Meta‐Analysis) extension for NMA (Table S1).^17^ Registration was completed in PROSPERO under the registration number CRD42023488481, and approval was granted by the institutional review board of the Tri‐Service General Hospital, National Defense Medical Center, Taipei, Taiwan (TSGHIRB No. B‐109‐29). The Institutional Review Board of the Tri‐Service General Hospital has confirmed that no ethical approval is required (TSGHIRB: B‐109‐29). The current study did not directly involve individual participant so that we did not have the chance to approach individual participant or explore individual participant’s information. Therefore, it would be impossible to obtain Consent to Participate in the current study.

### Database Searches and Study Identification

Database searches encompassed PubMed, Embase, ClinicalKey, Cochrane CENTRAL, ProQuest, ScienceDirect, Web of Science, and ClinicalTrials.gov (refer to Table S2). The systematic review and NMA search spanned from the earliest entry in each database to the most recent date of search (30 November 2023). Two authors (PTT and BYZ) independently executed electronic searches, evaluated titles and abstracts, and resolved eligibility through consensus. In addition, manual searches were conducted, scrutinizing reference lists of review articles for relevant studies. Language restrictions were not imposed on the search.

### Inclusion and Exclusion Criteria

The NMA adopted the PICOS model (population, intervention, comparison, outcome, and study) with specific criteria: (1) population: human participants at high risk of delirium development, varying across included studies (refer to Table S4); (2) intervention: pharmacologic treatment for delirium prevention; (3) comparison: control group with SoC or placebo control; (4) outcome: delirium development (incidence of delirium); and (5) study: RCT design. Delirium definitions were based on diverse diagnostic criteria in the included studies (refer to Table S4). To limit heterogeneity, only pharmacologic treatments were compared, excluding studies on different anesthesiologic methods or painkillers due to the prevalence of mixed applications in this field. Studies without a clear delirium definition or those only mentioning disorientation were also excluded.

To enhance the reliability of this NMA, only RCTs were included, applying the following criteria: (1) RCTs recruiting high‐risk delirium patients; (2) RCTs investigating delirium development differences by various pharmacologic treatments; and (3) control groups involving SoC or placebo control. Exclusion criteria covered (1) non‐RCTs; (2) RCTs recruiting baseline delirium patients; (3) RCTs not comparing different pharmacologic treatments; (4) RCTs unrelated to delirium outcomes; (5) RCTs related to emergence delirium prevention (a distinct outcome from delirium); and (6) RCTs on electroconvulsive therapy situations involving seizure induction, differing from other RCTs.

### Methodological Quality Appraisal

Two independent authors assessed the bias risk for each domain using the Cochrane Risk of Bias tool 1.0^18^ (interrater reliability, 0.87). Discrepancies were resolved by a third author. The Cochrane Risk of Bias tool 2.0^19^ was not employed due to the following rationale. In the assessment process with Cochrane Risk of Bias tool 2.0, we would have to evaluate the item of ‘*Blinding participants, most commonly through use of a placebo or sham intervention, may prevent such differences. If participants experienced side effects or toxicities that they knew to be specific to one of the interventions, answer this question “Yes” or “Probably yes”*’. However, this item was one of the key questions that was evaluated in this NMA. To be specific, in this NMA, we intended to evaluate whether the application of placebo would impact the incidence of delirium. Therefore, the Cochrane Risk of Bias tool 2.0 might be less suitable in this NMA under this consideration so that we chose the Cochrane Risk of Bias tool 1.0 to be our methodology appraisal tool.

### Outcome Definition

The primary outcome was delirium development (i.e. incidence of delirium), with varying definitions among RCTs, which are listed in the Table S4. The secondary outcome was the all‐cause mortality rate, indicating mortality during the study period irrespective of the cause.

### Data Extraction, Management, and Conversion

Two authors (PTT and BYZ) independently conducted data extraction, covering demographic information, study design, treatment details, and outcomes (primary and secondary) from the evaluated studies. In cases where necessary data were absent, the corresponding authors were contacted. Data processes adhered to recommendations outlined in the Cochrane Handbook for Systematic Reviews of Interventions and relevant medical literature.^20^

### Statistical Analyses

We used random‐effects model in this NMA with multiple treatment arms,^21^ using MetaInsight version 4.0.2 (Complex Reviews Support Unit, National Institute for Health Research) under a frequentist framework. MetaInsight was a web‐based platform for NMA that included the netmeta package in R software (R Foundation for Statistical Computing) for conducting frequentist statistical calculations.^22^

Initially, a forest plot and network plot were generated to display all pairwise comparisons from individual studies. Subsequently, forest plots were created for risk ratios (RRs) and 95% confidence intervals (CIs) in the primary outcome of incidence of delirium and secondary outcome of all‐cause mortality rate for each treatment compared with the control group to provide an overall summary of the effects.^23^ In the presentation of the forest plots, we chose the SoC arm as the reference arm based on the following rationale: since delirium frequently occurs in critical conditions, investigators typically provide at least basic treatment (referred to as SoC) to all patients based on ethical concerns. Therefore, the so‐called ‘placebo group’ in each RCT is, in fact, receiving ‘placebo plus SoC’. Therefore, the SoC should be considered the true foundation of every treatment arm. Therefore, we selected SoC to be our reference in the forest plot. To increase the clinical applicability of our findings, we calculated the surface under the cumulative ranking curve (SUCRA) to rank the relative superiority of individual treatments compared with others.^24^ Further, in order to improve readability, we presented the sort order of forest plot according to the relative superiority based on SUCRA results. We used a ‘node‐splitting’ approach to assess the consistency between treatment effect estimates derived from direct and indirect sources. This method breaks down the evidence for a specific comparison (node) into direct and indirect parts and is suitable for NMA when we have access to trial‐level data.^22^, ^25^ Statistical significance was defined as a two‐tailed *P* value <0.05.

### Sensitivity Analyses

To assess the robustness of our results, we conducted a sensitivity analysis by subgrouping RCTs based on the environment in which they were conducted. This approach was designed to more accurately evaluate the potential impacts on the patients' subjective perceptions of placebo treatments. To be specific, the sensitivity analysis involved subgroup analysis based on clinical settings (intensive care unit [ICU]/ward vs surgery) where trials were conducted. If an ICU stay was planned after a surgery in the original RCT, then it could be classified into the surgery subgroup here.

### General Declaration

This study conforms to the provisions of the Declaration of Helsinki.

## Results

### Eligibility of the studies

**Fig. 1:**
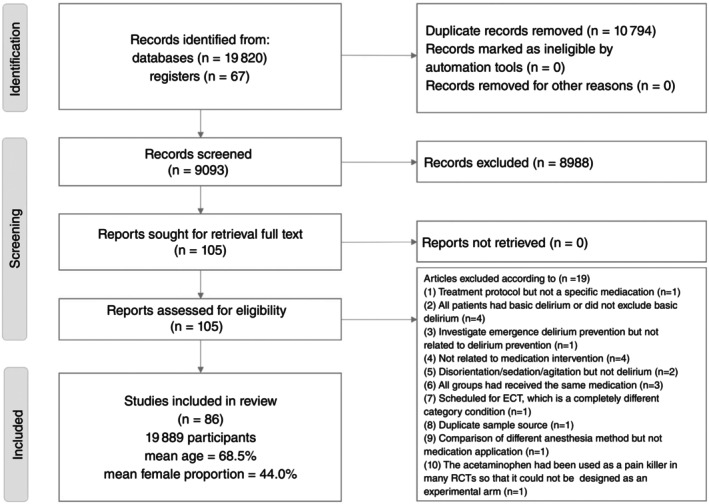
Flow diagram of the current network meta‐analysis. ECT, electroconvulsive therapy; RCT, randomized controlled trial.

Figure 1 illustrates the flowchart detailing the literature search and screening process for the current NMA. After excluding 19 articles for various reasons (Table S3), a total of 86 RCTs were included. The included studies comprised 19,889 participants (mean age, 68.5 years [range, 33.0–84.8 years]; mean proportion of females, 44.0% [range, 2.0%–80.0%]) (Table S4). A total of 34 experimental arms had been investigated (one placebo arm, one SoC arm, and 32 pharmacologic regimen arms).

### Primary outcome: incidence of delirium

**Fig. 2:**
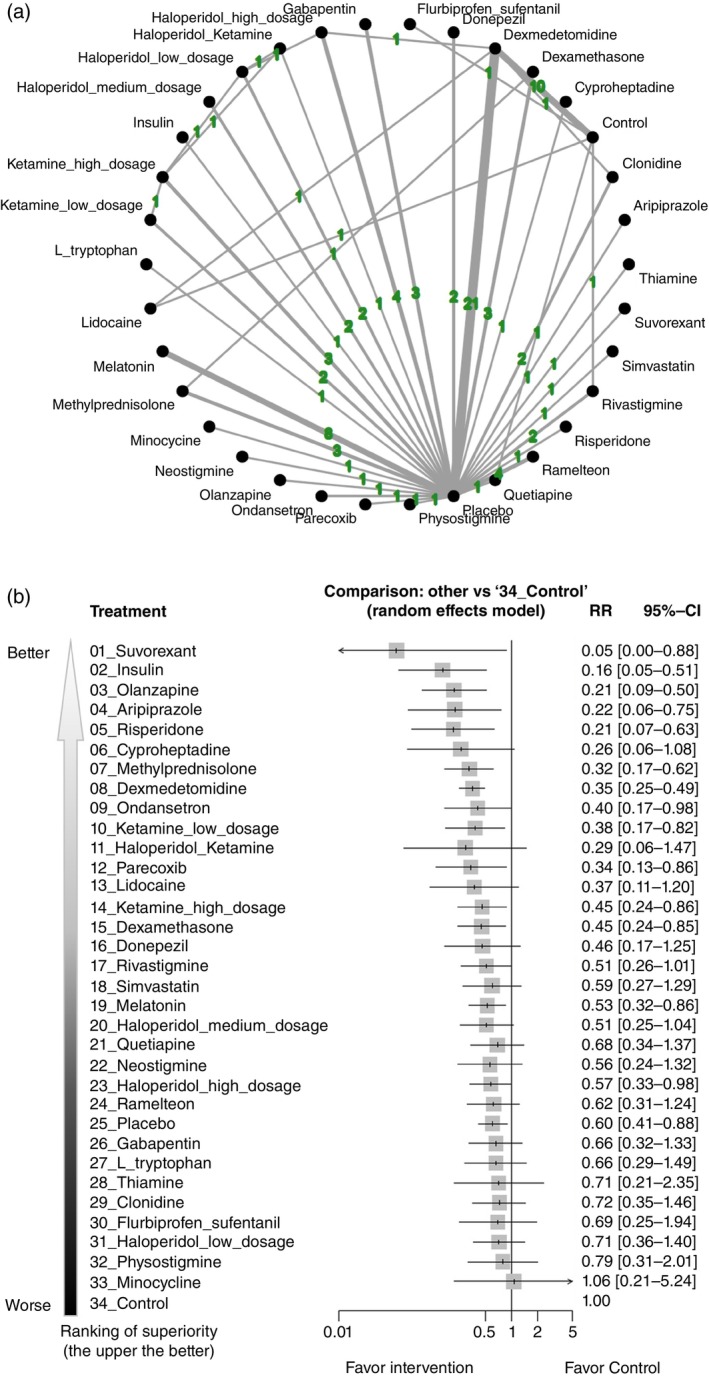
(a) Network plot of the primary outcomes: incidence of delirium. The lines between nodes represent direct comparisons in various trials, and the values overlaying the lines are the number of trials that compared these two treatments. (b) Forest plot of the incidence of delirium in reference to the control group (i.e. SoC). Specific treatments were associated with a decreased incidence of delirium than the control group (i.e. SoC) if the risk ratio was <1. CI, confidence interval; RR, risk ratio; SoC, standard of care.

In comparison to the control group (i.e. SoC alone), both the SoC plus placebo group (reported as RR, 0.60 [95% CI, 0.41–0.88]) and certain pharmacologic treatments were associated with a significantly lower incidence of delirium. Among these interventions, suvorexant ranked highest, while SoC plus placebo ranked 25th among all interventions (Fig. 2a,b, and Table S1).

### Sensitivity analysis by subgroup analysis of ICU/ward vs surgery settings

Within the subgroup of ICU/ward settings, the main results echoed similar findings, indicating that the SoC plus placebo group (RR, 0.51 [95% CI, 0.30–0.87) and eight pharmacologic treatments were linked to a significantly lower incidence of delirium compared with the control group (i.e. SoC alone). Among these interventions, ramelteon ranked the highest, while SoC plus placebo ranked 10th among all interventions (Figures S1a and S2b, and Table S5A).

In the subgroup of surgery settings, however, no significant difference was observed in the incidence of delirium between the SoC plus placebo group and the control group (i.e. SoC alone) (RR, 0.62 [95% CI, 0.28–1.34] in comparison with the control group). Five pharmacological interventions have been identified as having a reduced incidence of delirium compared with the SoC group. Among these interventions, insulin ranked the highest, while SoC plus placebo ranked 19th among all interventions (Figures S1b and S2b, and Table S5C).

### Secondary outcome: all‐cause mortality

None of the investigated treatments were associated with a significantly different all‐cause mortality rate compared with the control group (i.e. SoC alone) (Figures S1c and S2c, and Table S5D).

### Risk of bias, inconsistency, heterogeneity, publication bias, and GRADE

We identified that 86.7% (522 of 602 items), 10.1% (61 of 602 items), and 3.2% (19 of 602 items) of the included studies had low, unclear, and high risks of bias, respectively (Figures S3a,b). The inconsistency test, evaluating the assumption of consistency, showed no significant inconsistencies in the present NMA (Table S6).

## Discussion

To the best of our knowledge, this NMA represents the first attempt to systematically examine the potential impact of placebo prescription on the incidence of delirium from a statistical perspective. The primary findings of this NMA revealed a statistically significant difference in the incidence of delirium between the SoC plus placebo group and the control group (i.e. SoC alone) (RR, 0.60 [95% CI, 0.41–0.88]) in RCTs evaluating pharmacological prophylactic treatments in patients at a high risk of delirium development. Moreover, this statistical significance was observed specifically in the subgroup of ICU/ward settings but not in the subgroup of surgery settings. The lower incidence of delirium in the SoC plus placebo group suggests that RCTs on delirium prevention need to consider a placebo control arm rather than an SoC control because of observed placebo effects on incident delirium.

This observation is intriguing given that delirium development is typically considered an objective and severe outcome, rather than a subjective or minor outcome. While some may argue that placebo effects are limited to subjective outcomes such as pain,^9^ psychiatric disorders,^26^ or other minor symptoms (e.g. cough),^10^ an increasing body of evidence suggests that placebo effects, both positive and adverse, can influence objective and serious outcomes.^14^, ^27^, ^28^ Through various sensitivity and consistency tests, no evidence of transitivity and consistency assumption violations was detected.^29^

The distinction between the two subgroups indicates potentially different characteristics of recruited patients at baseline. This hypothesis could be supported by the different rankings of the same medication in these two subgroups. Specifically, ramelteon was ranked the first in ICU/ward subgroup but 27th in the surgery subgroup.

In light of these considerations, we propose two potential hypotheses to explain the placebo effect on the incidence of delirium in patients at high risk for developing delirium: (i) psychobehavioral impact, and (ii) immunomodulatory effect. Although previous evidence^16^ suggests that good adherence is associated with a beneficial effect regarding mortality in placebo arms, the psychobehavioral impact might play a lesser role in the current study. Specifically, since participants in delirium prevention trials are hospitalized patients, the risk of loss of follow‐up due to nonattendance is not applicable here. According to the second hypothesis, patient awareness of receiving a placebo may mimic the expected or conditioned pharmacological effects, resulting in endogenous neurotransmitter release,^30^ which involves the circuit of prefrontal cortex, limbic system, and hypothalamus. This neurobioimmunological network would support the findings in our subgroup analysis that the potential impact of placebo prescription might only exist in the situation where ‘patients might have the chance to know/guess the prescription of placebo’.

### Strengths and limitations

Several strengths characterize our NMA. First, it differentiates between the control group (i.e. SoC alone) and the SoC plus placebo group, a distinction not feasible in traditional pairwise meta‐analyses. Second, we examined RCTs exclusively to mitigate potential biases associated with observational studies or simple case‐control studies. Third, the considerable number of RCTs and participants (86 RCTs with 19,889 participants) provided ample opportunity for robust sensitivity tests and inconsistency assessments.

However, the current NMA has its limitations. First, some analyses were underpowered due to heterogeneity in participant characteristics, such as the settings where studies were conducted. To overcome this potential limitation, we performed subgroup analyses based on the different settings to eliminate the potential impact from the aforementioned heterogeneity. Second, because we specifically selected RCTs of pharmacologic treatment in patients at a high risk of delirium development, the observed placebo effects cannot be generalized to nonpharmacologic treatments. Last, despite distinguishing between the SoC plus placebo group and the control group (i.e. SoC alone), we lacked evidence for their direct comparison. Therefore, although the sensitivity test yielded insignificant findings, confirmation of this placebo effect requires direct evidence.

## Conclusion

**Fig. 3:**
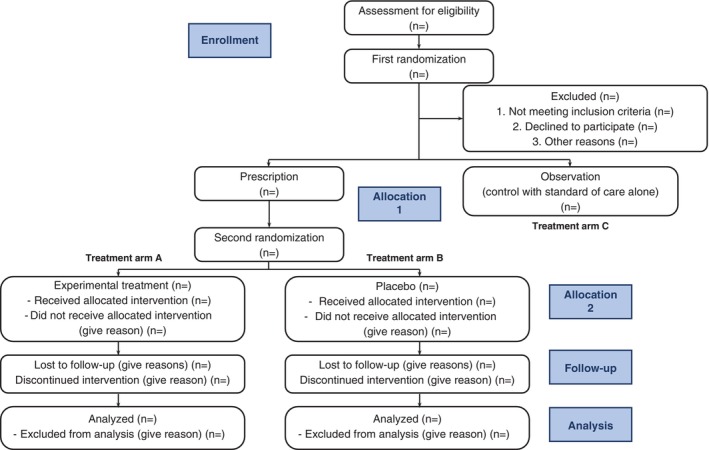
The revised CONSORT flowchart in the form of a three‐arm, parallel double randomization design. To ensure fair allocation to between observation (control with standard of care alone) and prescription (either with placebo or experimental treatment), we arranged the first randomization after enrolling eligible participants. Later, to achieve balance between placebo and experiment treatment, we arranged the second randomization for participants in the prescription group. Finally, this yielded three parallel groups after two randomization processes. CONSORT, Consolidated Standards of Reporting Trials.

The current NMA demonstrated a statistically lower average incidence of delirium in RCTs evaluating pharmacologically prophylactic treatments in patients at high risk of delirium development who were treated with SoC plus placebo compared with the control group (i.e. SoC alone). This suggests the potential existence of a placebo effect. Importantly, statistical significance was observed only in the subgroup of ICU/ward settings and not in the subgroup of surgery settings. These findings indicate that a portion of the efficacy of delirium prevention by pharmacologic treatment may be, at least partially, attributable to the placebo effect. Consequently, future trials of pharmacologically prophylactic treatments in patients at a high risk of delirium development should consider incorporating a three‐arm, parallel RCT, including a placebo group (i.e. placebo + SoC) and a control group (i.e. SoC alone), to provide more robust evidence (see Fig. 3).^15^ We recognize that, in real‐world settings, it is challenging to acquire a sufficient number of patients to achieve statistical significance in two‐arm RCTs. However, this proposed flowchart of a three‐arm RCT, centered on the inclusion of an SoC arm, is a blueprint to guide future clinical trials toward results that more closely reflect clinical reality.

## Disclosure statement

The authors report no financial interests or potential conflicts of interest. All data included in current study are available on reasonable request to the corresponding authors. The institutional review board of the Tri‐Service General Hospital has confirmed that no ethical approval was required for the current study (TSGHIRB: B‐109‐29).

## Author contribution

Bing‐Yan Zeng, Chih‐Sung Liang, Chih‐Wei Hsu, and Wei‐Te Lei, who contributed equally as first authors, took whole responsibility of the literature search, data extraction, data analysis, and manuscript drafting. Trevor Thompson, Yen‐Wen Chen, and Tien‐Yu Chen contributed to study design, concept formation, and manuscript revision. Ping‐Tao Tseng and Yow‐Ling Shiue, who contributed equally as corresponding authors, took whole responsibility of collection of information from the other authors, manuscript major revision, and manuscript submission.

## Supporting information


**Data S1.** Supporting Information.

## Data Availability

The data of this study are available on reasonable request.
