# Supplementary material for: Potential placebo bias in current trials of delirium prevention: a network meta‐analysis of 86 randomized controlled trials
Source: Psychiatry Clin Neurosci. 2025 Jun 18;79(8):481–7. doi: 10.1111/pcn.13850 (PMC12319638; doi:10.1111/pcn.13850)
Supplement: Supplementary file 1 — Data S1. Supporting Information. [file PCN-79-481-s001.docx]

**List of content in supplement materials**

**Potential placebo bias in current trials of delirium prevention**

**a network meta‐analysis of 86 randomized controlled trials**

*Bing-Yan Zeng, Chih-Sung Liang, Chih-Wei Hsu, Wei-Te Lei, Trevor Thompson, Yen-Wen Chen, Tien-Yu Chen, Ping-Tao Tseng, Yow-Ling Shiue*

| eFigure 1 | (A) Network structure of primary outcome: incidence of delirium: subgroup of ICU/ward setting |
| --- | --- |
|  | (B) Network structure of primary outcome: incidence of delirium: subgroup of surgery setting |
|  | (C) Network structure of secondary outcome: all-cause mortality rate |
| eFigure 2 | (A) Forest plot of primary outcome: incidence of delirium: subgroup of ICU/ward setting |
|  | (B) Forest plot of primary outcome: incidence of delirium: subgroup of surgery setting |
|  | (C) Forest plot of secondary outcome: all-cause mortality rate |
| eFigure 3 | (A) Overview of risk of bias |
|  | (B) Detailed risk of bias in each study |
| eTable 1 | PRISMA 2020 checklist of the current network meta-analysis |
| eTable 2 | Keyword used in each database and search results |
| eTable 3 | Excluded studies and reason |
| eTable 4 | Characteristics of the included studies |
| eTable 5 | (A): League table of primary outcome: incidence of delirium |
|  | (B): League table of primary outcome: incidence of delirium: subgroup of ICU/ward setting |
|  | (C): League table of primary outcome: incidence of delirium: subgroup of surgery setting |
|  | (D): League table of secondary outcome: all-cause mortality |
|  | (E): League table of NMA of safety profile: drop-out rate |
| eTable 6 | inconsistency within the network meta-analysis of primary outcome: incidence of delirium |

**eFigure 1A Network structure of primary outcome: incidence of delirium: subgroup of ICU/ward setting**

**eFigure 1B Network structure of primary outcome: incidence of delirium: subgroup of surgery setting**

**eFigure 1C Network structure of secondary outcome: all-cause mortality rate**

**Figure legend of eFigure 1A-1C**

The lines between nodes represent direct comparisons in various trials, and the numbers over the lines is the numbers of trials that compared these two treatments

**eFigure 2A Forest plot of primary outcome: incidence of delirium: subgroup of ICU/ward setting**

**
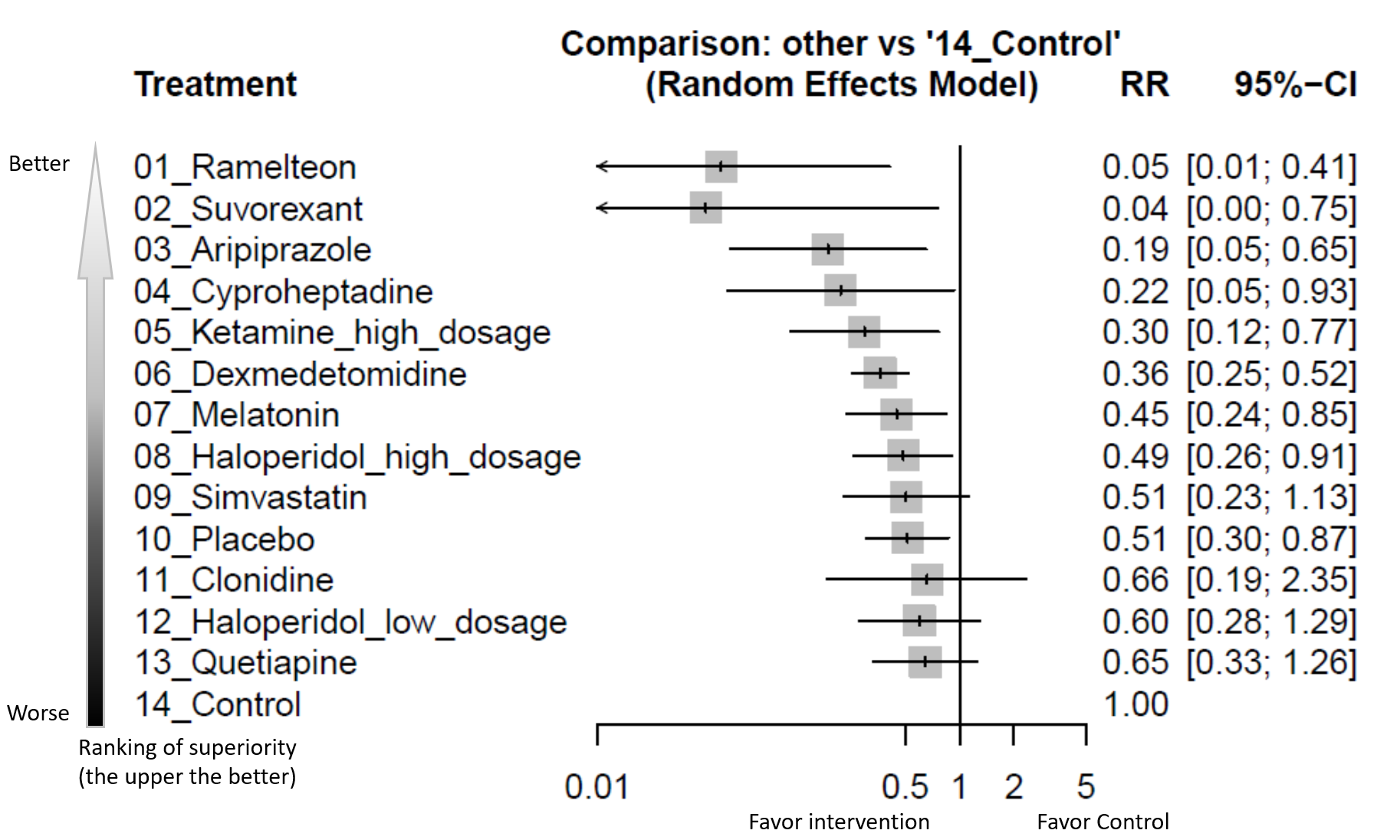
**

**eFigure 2B Forest plot of primary outcome: incidence of delirium: subgroup of surgery setting**

**
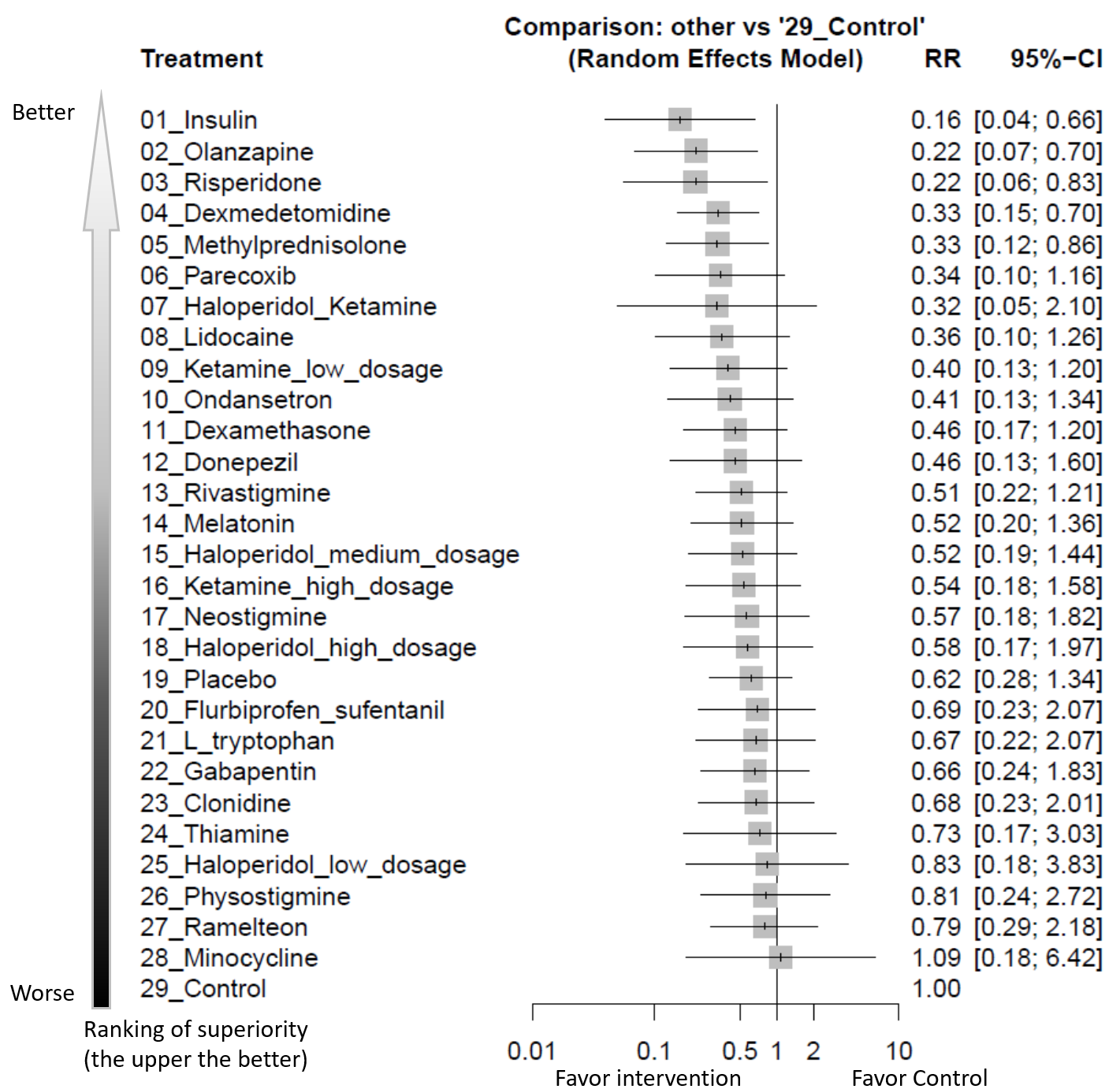
**

**eFigure 2C Forest plot of secondary outcome: all-cause mortality rate**

**
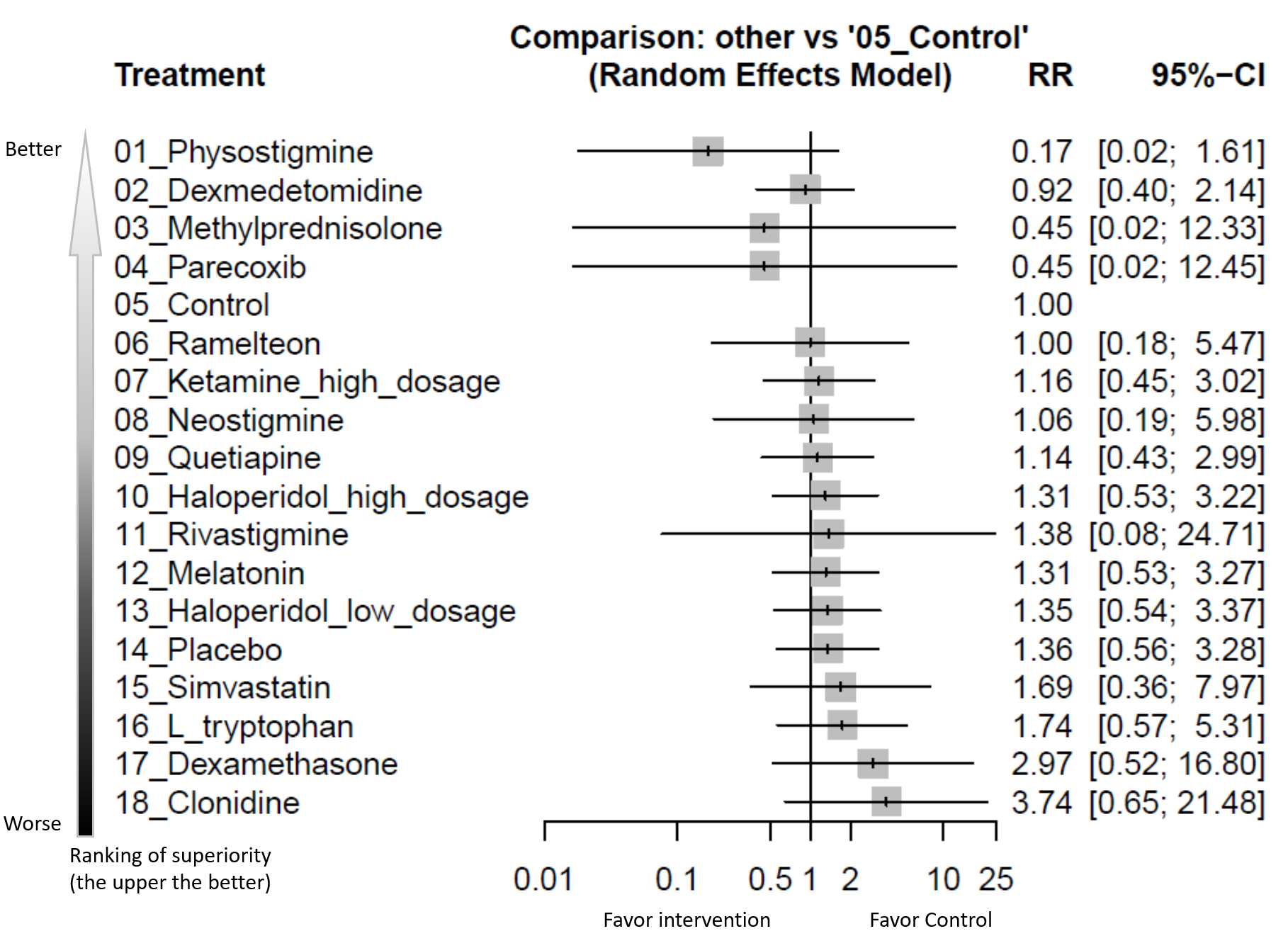
**

**Figure legend of eFigure 2A-2C**

Specific treatments were associated with more desirable results (i.e. less incidence of delirium or less all-cause mortality rate) than the control group (i.e. standard of care) if the risk ratio was less than 1.

**eFigure 3A overview of risk of bias**

**
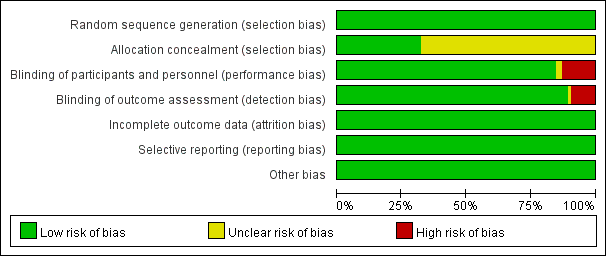
**

**eFigure 3B detailed risk of bias in each study**

**
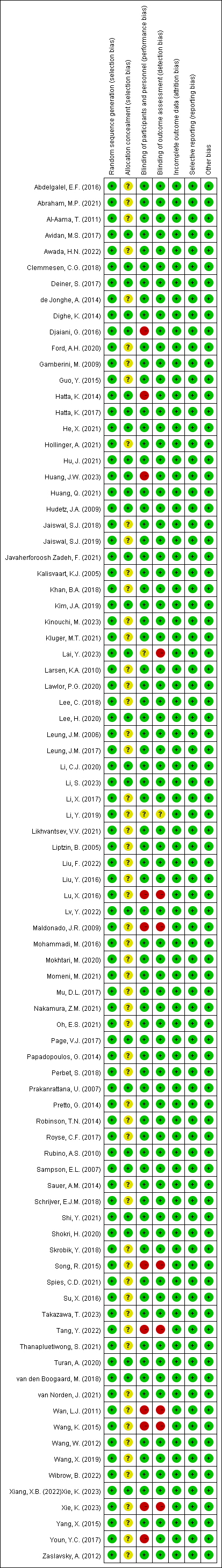
**

**
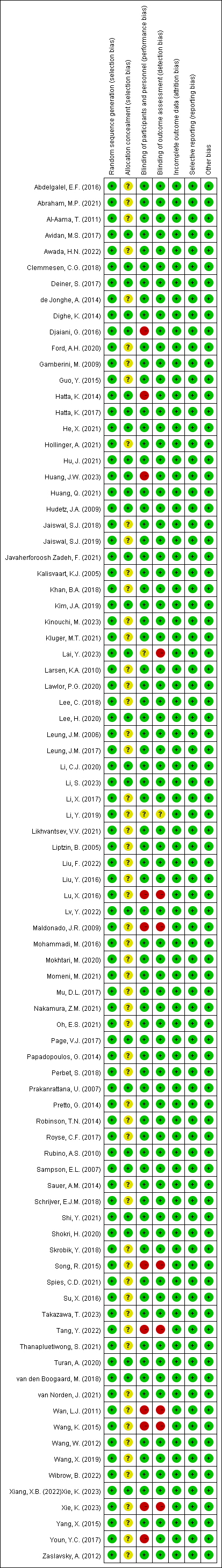
**

**
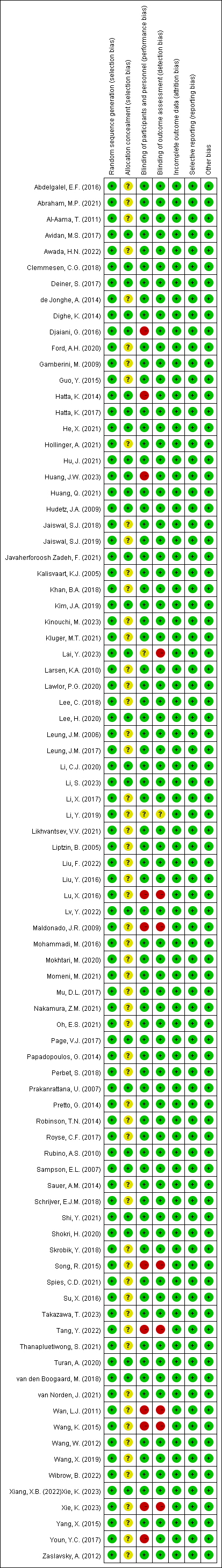
**

**eTable 1:** **PRISMA 2020 checklist of current network meta-analysis**

| **Section and Topic** | **Item #** | **Checklist item** | **Page where item is reported** |
| --- | --- | --- | --- |
| **TITLE** | | |  |
| Title | 1 | Identify the report as a systematic review. | 1 |
| **ABSTRACT** | | |  |
| Abstract | 2 | See the PRISMA 2020 for Abstracts checklist. | 7 |
| **INTRODUCTION** | | |  |
| Rationale | 3 | Describe the rationale for the review in the context of existing knowledge. | 8-9 |
| Objectives | 4 | Provide an explicit statement of the objective(s) or question(s) the review addresses. | 8-9 |
| **METHODS** | | |  |
| Eligibility criteria | 5 | Specify the inclusion and exclusion criteria for the review and how studies were grouped for the syntheses. | 10-11 |
| Information sources | 6 | Specify all databases, registers, websites, organisations, reference lists and other sources searched or consulted to identify studies. Specify the date when each source was last searched or consulted. | 10-11 |
| Search strategy | 7 | Present the full search strategies for all databases, registers and websites, including any filters and limits used. | 10-11 |
| Selection process | 8 | Specify the methods used to decide whether a study met the inclusion criteria of the review, including how many reviewers screened each record and each report retrieved, whether they worked independently, and if applicable, details of automation tools used in the process. | 10-11 |
| Data collection process | 9 | Specify the methods used to collect data from reports, including how many reviewers collected data from each report, whether they worked independently, any processes for obtaining or confirming data from study investigators, and if applicable, details of automation tools used in the process. | 10-11 |
| Data items | 10a | List and define all outcomes for which data were sought. Specify whether all results that were compatible with each outcome domain in each study were sought (e.g. for all measures, time points, analyses), and if not, the methods used to decide which results to collect. | 10-11 |
|  | 10b | List and define all other variables for which data were sought (e.g. participant and intervention characteristics, funding sources). Describe any assumptions made about any missing or unclear information. | 11-12 |
| Study risk of bias assessment | 11 | Specify the methods used to assess risk of bias in the included studies, including details of the tool(s) used, how many reviewers assessed each study and whether they worked independently, and if applicable, details of automation tools used in the process. | 11-12 |
| Effect measures | 12 | Specify for each outcome the effect measure(s) (e.g. risk ratio, mean difference) used in the synthesis or presentation of results. | 11-12 |
| Synthesis methods | 13a | Describe the processes used to decide which studies were eligible for each synthesis (e.g. tabulating the study intervention characteristics and comparing against the planned groups for each synthesis (item #5)). | 11-12 |
|  | 13b | Describe any methods required to prepare the data for presentation or synthesis, such as handling of missing summary statistics, or data conversions. | 11-12 |
|  | 13c | Describe any methods used to tabulate or visually display results of individual studies and syntheses. | 11-12 |
|  | 13d | Describe any methods used to synthesize results and provide a rationale for the choice(s). If meta-analysis was performed, describe the model(s), method(s) to identify the presence and extent of statistical heterogeneity, and software package(s) used. | 12-13 |
|  | 13e | Describe any methods used to explore possible causes of heterogeneity among study results (e.g. subgroup analysis, meta-regression). | 12-13 |
|  | 13f | Describe any sensitivity analyses conducted to assess robustness of the synthesized results. | 12-13 |
| Reporting bias assessment | 14 | Describe any methods used to assess risk of bias due to missing results in a synthesis (arising from reporting biases). | 12-13 |
| Certainty assessment | 15 | Describe any methods used to assess certainty (or confidence) in the body of evidence for an outcome. | 12-13 |
| **RESULTS** | | |  |
| Study selection | 16a | Describe the results of the search and selection process, from the number of records identified in the search to the number of studies included in the review, ideally using a flow diagram. | 14-15, Fig 1 |
|  | 16b | Cite studies that might appear to meet the inclusion criteria, but which were excluded, and explain why they were excluded. | 14-15, eTab 2 |
| Study characteristics | 17 | Cite each included study and present its characteristics. | 14-15, eTab 3 |
| Risk of bias in studies | 18 | Present assessments of risk of bias for each included study. | 14-15, eFig 2 |
| Results of individual studies | 19 | For all outcomes, present, for each study: (a) summary statistics for each group (where appropriate) and (b) an effect estimate and its precision (e.g. confidence/credible interval), ideally using structured tables or plots. | 14-15, eTab 3 |
| Results of syntheses | 20a | For each synthesis, briefly summarise the characteristics and risk of bias among contributing studies. | 14-15, eFig 3 |
|  | 20b | Present results of all statistical syntheses conducted. If meta-analysis was done, present for each the summary estimate and its precision (e.g. confidence/credible interval) and measures of statistical heterogeneity. If comparing groups, describe the direction of the effect. | 14-15, Fig 2, eFig 2 |
|  | 20c | Present results of all investigations of possible causes of heterogeneity among study results. | 14-15, eTab 6 |
|  | 20d | Present results of all sensitivity analyses conducted to assess the robustness of the synthesized results. | 14-15 |
| Reporting biases | 21 | Present assessments of risk of bias due to missing results (arising from reporting biases) for each synthesis assessed. | 14-15, eFig 3 |
| Certainty of evidence | 22 | Present assessments of certainty (or confidence) in the body of evidence for each outcome assessed. | 14-15, eTab 6 |
| **DISCUSSION** | | |  |
| Discussion | 23a | Provide a general interpretation of the results in the context of other evidence. | 16-17 |
|  | 23b | Discuss any limitations of the evidence included in the review. | 17-18 |
|  | 23c | Discuss any limitations of the review processes used. | 17-18 |
|  | 23d | Discuss implications of the results for practice, policy, and future research. | 19 |
| **OTHER INFORMATION** | | |  |
| Registration and protocol | 24a | Provide registration information for the review, including register name and registration number, or state that the review was not registered. | 7 |
|  | 24b | Indicate where the review protocol can be accessed, or state that a protocol was not prepared. | 7 |
|  | 24c | Describe and explain any amendments to information provided at registration or in the protocol. | 7 |
| Support | 25 | Describe sources of financial or non-financial support for the review, and the role of the funders or sponsors in the review. | 20 |
| Competing interests | 26 | Declare any competing interests of review authors. | 20 |
| Availability of data, code and other materials | 27 | Report which of the following are publicly available and where they can be found: template data collection forms; data extracted from included studies; data used for all analyses; analytic code; any other materials used in the review. | 20 |

The current checklist followed the latest PRISMA 2020 guideline.^1^

**eTable 2: Keywords in each database and search result**

| Database | Keyword | Filter | Date | Result |
| --- | --- | --- | --- | --- |
| PubMed | (prevention OR prophylactic OR prophylaxis OR preventive OR prevent) AND (delirium) AND (random OR randomized OR randomised) | RCT | 2023/11/30 | 553 |
| Embase | (prevention OR prophylactic OR prophylaxis OR preventive OR prevent) AND (delirium) AND (random OR randomized OR randomised) | N/A | 2023/11/30 | 1613 |
| ClinicalKey | (prevention OR prophylactic OR prophylaxis OR preventive OR prevent) AND (delirium) AND (random OR randomized OR randomised) | N/A | 2023/11/30 | 1234 |
| Cochrane CENTRAL | (prevention OR prophylactic OR prophylaxis OR preventive OR prevent) AND (delirium) AND (random OR randomized OR randomised) | N/A | 2023/11/30 | 1298 |
| ProQuest | (prevention OR prophylaxis) AND (delirium) AND (random OR randomized OR randomised) | N/A | 2023/11/30 | 8982 |
| ScienceDirect | (prevention OR prophylactic OR prophylaxis OR preventive OR prevent) AND (delirium) AND (random OR randomized OR randomised) | research article | 2023/11/30 | 5117 |
| Web of Science | (prevention OR prophylactic OR prophylaxis OR preventive OR prevent) AND (delirium) AND (random OR randomized OR randomised) | N/A | 2023/11/30 | 1023 |
| ClinicalTrials.gov | (prevention OR prophylactic OR prophylaxis OR preventive OR prevent) AND (delirium) AND (random OR randomized OR randomised) | N/A | 2023/11/30 | 67 |

Abbreviation: N/A: not applied

**eTable 3: Excluded studies and reason**

| Study name | Title | Reason |
| --- | --- | --- |
| Aizawa, K. (2002)^2^ | A novel approach to the prevention of postoperative delirium in the elderly after gastrointestinal surgery | A treatment protocol but not a specific medication |
| Al-Qadheeb, N.S. (2016)^3^ | Preventing ICU Subsyndromal Delirium Conversion to Delirium With Low-Dose IV Haloperidol: A Double-Blind, Placebo-Controlled Pilot Study | All patients had baseline delirium |
| Bartoszek, M. (2023)^4^ | The Effectiveness of Dexmedetomidine as a Prophylactic Treatment for Emergence Delirium Among Combat Veterans With High Anxiety: A Randomized Placebo-Controlled Trial | Investigate emergence delirium prevention but not related to delirium prevention |
| Chen, J. (2022)^5^ | Effect of Preoperative Oral Saline Administration on Postoperative Delirium in Older Persons: A Randomized Controlled Trial | Not related to medication intervention |
| Fukata, S. (2014)^6^ | Haloperidol prophylaxis does not prevent postoperative delirium in elderly patients: a randomized, open-label prospective trial | Including patients with baseline NEECHAM confusion scale between 20-24, which was Mild to early development of confusion (mild delirium) |
| Fukata, S. (2017)^7^ | Haloperidol prophylaxis for preventing aggravation of postoperative delirium in elderly patients: a randomized, open-label prospective trial | All patients had baseline delirium |
| Kawazoe, Y. (2017)^8^ | Effect of Dexmedetomidine on Mortality and Ventilator-Free Days in Patients Requiring Mechanical Ventilation With Sepsis: A Randomized Clinical Trial | Did not exclude patients with baseline delirium |
| Levanen, J. (1995)^9^ | Dexmedetomidine premedication attenuates ketamine-induced cardiostimulatory effects and postanesthetic delirium | Disorientation but not delirium |
| Niu, J.J. (2023)^10^ | Effect of Different Administration Routes of Dexmedetomidine on Postoperative Delirium in Elderly Patients Undergoing Elective Spinal Surgery: A Prospective Randomized Double-Blinded Controlled Trial | All groups had received dexmedetomidine |
| Qiu, Z. (2020)^11^ | Preventive effect of dexmedetomidine on postictal delirium after electroconvulsive therapy: A randomised controlled study | Scheduled for ECT, which is a completely different category condition |
| Rood, P.J.T. (2019)^12^ | Prophylactic Haloperidol Effects on Long-term Quality of Life in Critically Ill Patients at High Risk for Delirium: Results of the REDUCE Study | Duplicate samples with van den Boogaard, M. (2018) |
| Shehabi, Y. (2019)^13^ | Early Sedation with Dexmedetomidine in Critically Ill Patients | Agitation and sedation but not delirium |
| Siripoonyothai, S. (2021)^14^ | Comparison of postoperative delirium within 24 hours between ketamine and propofol infusion during cardiopulmonary bypass machine: A randomized controlled trial | Comparison of different anesthesia method but not medication application |
| Subramaniam, B. (2019)^15^ | Effect of Intravenous Acetaminophen vs Placebo Combined With Propofol or Dexmedetomidine on Postoperative Delirium Among Older Patients Following Cardiac Surgery: The DEXACET Randomized Clinical Trial | The acetaminophen had been used as a pain killer in many RCTs so that it could not be designed as an experimental arm |
| Tang, C. (2020)^16^ | Dexmedetomidine with sufentanil in intravenous patient-controlled analgesia for relief from postoperative pain, inflammation and delirium after esophageal cancer surgery | Both groups had received dexmedetomidine |
| Wang, H.B. (2023)^17^ | A randomised controlled trial of dexmedetomidine for delirium in adults undergoing heart valve surgery | Both groups had received dexmedetomidine |
| Wong, J. (2022)^18^ | The prevention of delirium in elderly surgical patients with obstructive sleep apnea (PODESA): a randomized controlled trial | Not related to medication intervention |
| Xin, X. (2017)^19^ | Effects of preconditioning with hypertonic saline solution on postoperative delirium in the aged | Not related to medication intervention |
| Xin, X. (2017)^20^ | Hypertonic saline for prevention of delirium in geriatric patients who underwent hip surgery | Not related to medication intervention |

**eTable 4: Characteristics of the included studies**

| Study name | Baseline eligible criteria | Setting | Comparison | Subjects | Mean age (year) | Female (%) | Tools for delirium Dx | Delirium occurrence | Result | Region |
| --- | --- | --- | --- | --- | --- | --- | --- | --- | --- | --- |
| Huang, J.W. (2023)^21^ | Geriatric intertrochanteric fracture require surgery | Surgery | Dexamethasone 10mg Placebo (normal saline) | 80 80 | 84.5 85.0 | 62.5 60.0 | Nu-DESC | 9 21 | lower occurrence of delirium in dexamethasone group | China |
| Kinouchi, M. (2023)^22^ | Scheduled for major elective surgery under general anesthesia | Surgery | Ramelteon 8 mg/day Placebo | 54 49 | 78.1±6.9 75.4±5.6 | 48.1 46.9 | CAM-ICU | 7 4 | No statistically significant difference | Japan |
| Lai, Y. (2023)^23^ | Planed to elective thoracoscopic lobectomy or segmentectomy | Surgery | Lidocaine 1.0 mg/kg/h Dexmedetomidine 1.0 ug/kg/h Standard of care | 30 29 30 | 70.9±6.0 70.6±5.3 71.2±5.1 | 53.3 65.5 63.3 | 3D-CAM | 4 5 9 | No statistically significant difference | China |
| Li, S. (2023)^24^ | Patients with frontotemporal brain tumors scheduled for elective craniotomy with general anesthesia | Surgery | Dexmedetomidine 0.6 ug/kg/h Placebo (normal saline) | 130 130 | 45.0 45.0 | 44.6 50.8 | CAM-ICU | 28 60 | lower occurrence of delirium in dexmedetomidine group | China |
| Takazawa, T. (2023)^25^ | Scheduled for total knee arthroplasty under general anesthesia | Surgery | Minocycline 200 mg/day Placebo | 90 95 | 74.5 75.0 | 77.8 80.0 | CAM-ICU | 5 3 | No statistically significant difference | Japan |
| Xie, K. (2023)^26^ | Scheduled for elective surgery for gastrointestinal or lung tumors | ICU/Ward | Dexmedetomidine 3 ug/kg Standard of care (no extra placebo application in the fluid) | 117 119 | 67.9±5.6 68.6±5.5 | 29.1 35.3 | CAM-ICU | 4 12 | lower occurrence of delirium in dexmedetomidine group | China |
| Awada, H.N. (2022)^27^ | Scheduled for open liver resection | Surgery | Methylprednisolone 10mg/kg Dexamethasone 8mg | 26 27 | NA | 52.8 | 3D-CAM | 0 5 | lower occurrence of delirium in methylprednisolone group | Denmark |
| Liu, F. (2022)^28^ | Scheduled for colon carcinoma surgery was performed under general anesthesia | Surgery | Neostigmine 0.04 mg/kg Placebo | 196 205 | 64.2±10.5 63.3±10.4 | 45.9 44.9 | CAM | 38 43 | No statistically significant difference | China |
| Lv, Y. (2022)^#29^ | Scheduled for total hip arthroplasty surgery | Surgery | Dexmedetomidine 0.1 ug/kg/h Placebo (normal saline) | 152 157 | 67.9±5.9 68.4±6.6 | 52.0 52.9 | CAM | 21 46 | lower occurrence of delirium in dexmedetomidine group | China |
| Tang, Y. (2022)^30^ | Scheduled for liver lobectomy | Surgery | Dexmedetomidine 0.3-0.6 ug/kg/h Standard of care | 80 40 | 68.0±7.2 68.7±6.6 | 45.0 47.5 | CAM | 5 9 | lower occurrence of delirium in dexmedetomidine group | China |
| Wibrow, B. (2022)^31^ | Admitted to ICU with expected stay greater than 72 hours | ICU/Ward | Melatonin 4mg/day Placebo | 419 422 | 61.9±15.1 61.9±15.2 | 40.9 33.6 | CAM-ICU | 147 138 | No statistically significant difference | Australia |
| Xiang, X.B. (2022)^32^ | Scheduled for laparoscopic gastrointestinal surgery | Surgery | Methylprednisolone 2mg/kg Placebo (normal saline) | 84 84 | 71.0 70.0 | 39.3 35.7 | CAM | 9 20 | lower occurrence of delirium in methylprednisolone group | China |
| Abraham, M.P. (2021)^33^ | Admitted to the surgical trauma ICU | ICU/Ward | Quetiapine 25 mg/day Standard of care | 22 49 | 55.0±24.0 59.0±18.0 | 27.3 44.9 | CAM-ICU | 10 38 | lower occurrence of delirium in quetiapine group | USA |
| He, X. (2021)^34^ | Scheduled for elective intracranial procedure for cerebral tumors under general anesthesia | Surgery | Dexmedetomidine 0.1 ug/kg/h Placebo (normal saline) | 30 30 | 51.0±11.0 48.0±11.0 | 56.7 50.0 | CAM-ICU | 0 4 | lower occurrence of delirium in dexmedetomidine group | China |
| Hollinger, A. (2021)^35^ | Scheduled for elective or emergency surgery under general or combined anaesthesia | Surgery | Haloperidol 0.391mg/day (mean BW 78.2 kg x 5 ug/kg) Ketamine 1.0 mg/kg Haloperidol + Ketamine Placebo | 45 47 46 44 | 73.4±6.3 73.4±6.1 73.2±5.2 74.8±6.6 | 35.6 44.7 37.0 54.5 | CAM | 5 3 2 4 | No statistically significant difference | Switzerland |
| Hu, J. (2021)^36^ | Scheduled for an open transthoracic oesophagectomy under general endotracheal anaesthesia | Surgery | Dexmedetomidine 0.4 ug/kg/h Placebo (normal saline) | 90 87 | 69.6±4.5 69.1±5.1 | 16.7 18.4 | RSA | 15 32 | lower occurrence of delirium in dexmedetomidine group | China |
| Huang, Q. (2021)^37^ | Scheduled for laparoscopic radical gastrointestinal tumor surgery | Surgery | Insulin intranasal 20 U Placebo (normal saline) | 40 40 | 68.0 65.0 | 37.5 47.5 | RASS | 5 19 | lower occurrence of delirium in insulin group | China |
| Javaherforoosh Zadeh, F. (2021)^38^ | Scheduled for elective on-pump coronary artery bypass graft surgery | Surgery | Melatonin total 9mg Placebo | 30 30 | 60.3±9.5 62.9±8.1 | 33.3 26.6 | CAM-ICU | 3 14 | lower occurrence of delirium in melatonin group | Iran |
| Kluger, M.T. (2021)^39^ | Admitted for hip fracture | Surgery | Dexamethasone 20mg Placebo | 40 39 | 81.4±7.2 81.4±8.9 | 55.0 61.5 | 4AT | 6 9 | No statistically significant difference | New Zealand |
| Likhvantsev, V.V. (2021)^40^ | Scheduled for elective coronary artery bypass grafting (CABG) or valve surgery | Surgery | Dexmedetomidine 0.7 ug/kg/h Placebo (normal saline) | 84 85 | 62.6±6.7 62.4±7.2 | 29.4 26.2 | CAM-ICU | 6 16 | lower occurrence of delirium in dexmedetomidine group | Russia |
| Momeni, M. (2021)^41^ | Scheduled for cardiac surgery with cardiopulmonary bypass | ICU/Ward | Dexmedetomidine 0.4 ug/kg/h Placebo (normal saline) | 177 172 | 71.0±10.0 70.0±11.0 | 26.8 21.7 | RASS | 31 33 | No statistically significant difference | Belgium |
| Nakamura, Z.M. (2021)^42^ | Scheduled for allogeneic hematopoietic stem cell transplantation | Surgery | Thiamine 200 mg IV TID Placebo (normal saline) | 28 33 | 54.9±12.5 53.6±14.7 | 39.3 39.4 | DRS | 7 7 | No statistically significant difference | USA |
| Oh, E.S. (2021)^43^ | Scheduled for elective primary or revision joint (hip or knee) replacement | Surgery | Ramelteon 8 mg/day Placebo | 33 38 | 74.3±5.5 75.4±5.0 | 58.0 63.0 | AldS | 10 5 | No statistically significant difference | USA |
| Shi, Y. (2021)^44^ | Received percutaneous transluminal coronary intervention in ICU | ICU/Ward | Melatonin 3mg/day Placebo | 148 149 | 71.5±6.7 71.6±6.6 | 37.2 40.3 | CAM-ICU | 40 59 | lower occurrence of delirium in melatonin group | China |
| Spies, C.D. (2021)^45^ | Scheduled for liver resection | Surgery | Physostigmine 0.01-0.02mg/kg Placebo | 130 131 | 61.0 60.0 | 45.4 38.2 | DSM-IV criteria | 26 20 | No statistically significant difference | Germany |
| Thanapluetiwong, S. (2021)^46^ | Acutely hospitalized in a medical specialty | ICU/Ward | Quetiapine 12.5 mg/day Placebo | 57 57 | 75.4±7.5 75.2±6.9 | 43.9 47.4 | CAM | 8 5 | No statistically significant difference | Thailand |
| van Norden, J. (2021)^47^ | Scheduled for major elective cardiac or major open abdominal surgery | Surgery | Dexmedetomidine 0.7 ug/kg/h Placebo | 28 32 | 70.4±7.1 70.5±6.2 | 32.1 28.1 | CAM-ICU | 5 14 | lower occurrence of delirium in dexmedetomidine group | Germany |
| Ford, A.H. (2020)^48^ | Scheduled for coronary artery bypass grafting and/or valve replacement | Surgery | Melatonin 3mg/day Placebo | 98 104 | 69.0±8.3 67.6±8.0 | 24.8 19.0 | CAM | 21 21 | No statistically significant difference | Australia |
| Lawlor, P.G. (2020)^49^ | Diagnosis of advanced cancer and admitted to the inpatient PCU | ICU/Ward | Melatonin 3mg/day Placebo | 30 30 | 67.0 67.0 | 46.7 43.3 | CAM | 11 10 | No statistically significant difference | Canada |
| Lee, H. (2020)^50^ | Scheduled for liver transplantation | Surgery | Dexmedetomidine 0.1 ug/kg/h + 0.9% saline Placebo (normal saline) | 100 101 | 56.0 55.0 | 29.0 27.7 | CAM-ICU | 9 6 | No statistically significant difference | Korea |
| Li, C.J. (2020)^51^ | Scheduled for elective major non-cardiac surgery under general anaesthesia | Surgery | Dexmedetomidine 0.6 ug/kg/h Placebo (normal saline) | 309 310 | 69.0±6.6 69.0±6.4 | 59.2 61.3 | CAM-ICU | 17 32 | lower occurrence of delirium in dexmedetomidine group | China |
| Mokhtari, M. (2020)^52^ | Admitted to the ICU post-neurosurgical intervention | ICU/Ward | Aripiprazole 15mg/day Placebo | 20 20 | 44.5±16.5 49.3±14.5 | 15.0 40.0 | RASS | 4 11 | lower occurrence of delirium in aripiprazole group | Iran |
| Shokri, H. (2020)^53^ | Scheduled for elective isolated coronary artery bypass grafting (CABG) | Surgery | Dexmedetomidine 0.7-1.2 ug/kg/h Clonidine | 144 142 | 63.8±3.3 64.4±4.8 | 46.5 57.7 | RASS | 12 23 | lower occurrence of delirium in dexmedetomidine group | Egypt |
| Turan, A. (2020)^54^ | Scheduled for cardiac surgery with bypass | Surgery | Dexmedetomidine 0.1-0.4 ug/kg/h Placebo (normal saline) | 398 396 | 63.0±11.0 62.0±12.0 | 32.7 27.0 | CAM-ICU | 67 46 | No statistically significant difference | USA |
| Jaiswal, S.J. (2019)^55^ | Scheduled for elective pulmonary thromboendarterectomy | Surgery | Ramelteon 8 mg/day Placebo | 59 58 | 58.1±14.1 56.1±15.8 | 50.8 50.0 | CAM-ICU | 19 22 | No statistically significant difference | USA |
| Kim, J.A. (2019)^56^ | Scheduled for elective video-assisted thoracoscopic lobectomy/segmentectomy for lung cancer | Surgery | Dexmedetomidine 0.5 ug/kg/h Placebo (normal saline) | 60 60 | 63.0 59.0 | 53.3 50.0 | CAM-ICU | 15 15 | No statistically significant difference | Korea |
| Li, Y. (2019)^57^ | Every considered ICU patient was under continuous sedation and analgesia for 48 hours or longer | ICU/Ward | Dexmedetomidine 0.8 ug/kg/h Standard of care | 64 62 | 43.0±15.0 45.0±13.0 | 42.2 45.2 | CAM-ICU | 18 34 | lower occurrence of delirium in dexmedetomidine group | China |
| Wang, X. (2019)^58^ | Scheduled for selective major noncardiac surgeries | Surgery | Flurbiprofen axetil + sufentanil Standard of care (sufentanil) | 70 70 | 69.5±4.1 69.3±4.6 | 51.4 41.4 | CAM | 9 13 | No statistically significant difference | China |
| Clemmesen, C.G. (2018)^59^ | Old age admitted acutely after hip fracture | Surgery | Methylprednisolone sodium succinate 125 mg/2ml Placebo | 59 58 | 79.0±8.0 81.0±9.0 | 62.7 65.5 | CAM | 10 19 | lower occurrence of delirium in methylprednisolone group | Denmark |
| Jaiswal, S.J. (2018)^60^ | Admitted to internal medicine service, with expected stay of at least 48 hours | ICU/Ward | Melatonin 3mg/day Placebo | 43 44 | 81.2±7.3 80.1±8.3 | 58.1 65.9 | CAM | 9 4 | No statistically significant difference | USA |
| Khan, B.A. (2018)^61^ | Scheduled for thoracic surgery | Surgery | Haloperidol 1.5 mg/day Placebo | 68 67 | 60.0 62.3 | 32.4 19.4 | CAM-ICU | 15 19 | No statistically significant difference | USA |
| Lee, C. (2018)^62^ | Scheduled for laparoscopic major non-cardiac surgery under general anesthesia | Surgery | Dexmedetomidine Placebo (normal saline) | 209 109 | 72.7±6.0 73.8±6.1 | 55.0 56.9 | CAM | 30 27 | lower occurrence of delirium in dexmedetomidine group | Korea |
| Perbet, S. (2018)^63^ | Expected to require mechanical ventilation for longer than 24 hours | ICU/Ward | Ketamine 2.5 mg/kg then continuous infusion Placebo | 80 82 | 63.0±15.0 61.0±14.0 | 30.0 35.4 | CAM-ICU | 17 30 | lower occurrence of delirium in ketamine group | France |
| Schrijver, E.J.M. (2018)^64^ | Acutely hospitalised through the emergency department | ICU/Ward | Haloperidol 2mg/day Placebo | 118 124 | 83.5±6.2 83.4±6.4 | 51.7 58.9 | DSM-IV criteria | 23 18 | No statistically significant difference | the Netherlands |
| Skrobik, Y. (2018)^65^ | Admitted to the ICU with intermittent or continuous sedatives and expected to require 48 or more hours of ICU care | ICU/Ward | Dexmedetomidine 0.7 ug/kg/h Placebo (D5W) | 50 50 | 62.1±13.2 62.4±14.1 | 38.0 34.0 | RASS | 10 23 | lower occurrence of delirium in dexmedetomidine group | Multiple countries |
| van den Boogaard, M. (2018)^66^ | Anticipated ICU stay for at least 2 days | ICU/Ward | Haloperidol 1mg/day Haloperidol 2mg/day Placebo | 350 732 707 | 66.1±12.6 66.7±12.7 67.0±12.6 | 41.1 37.3 38.6 | CAM-ICU | 139 244 233 | No statistically significant difference | the Netherlands |
| Avidan, M.S. (2017)^67^ | Scheduled for major open cardiac or non-cardiac surgerie under general anaesthesia. | Surgery | Ketamine 0.5 mg/kg Ketamine 1.0 mg/kg Placebo (normal saline) | 227 223 222 | 70.0±7.2 70.0±7.3 70.0±6.9 | 36.6 37.7 39.2 | CAM-ICU | 40 47 44 | No statistically significant difference | Multiple countries |
| Deiner, S. (2017)^68^ | Having at least a 2-day hospital stay | ICU/Ward | Dexmedetomidine 0.5 ug/kg/h Placebo (normal saline) | 189 201 | 74.0 74.0 | 51.3 51.2 | CAM-ICU | 34 35 | No statistically significant difference | USA |
| Hatta, K. (2017)^69^ | Admitted due to emergency | ICU/Ward | Suvorexant 15mg/day Placebo | 36 36 | 78.5±6.5 78.3±6.2 | 36.1 47.2 | DSM-5 criteria | 0 6 | lower occurrence of delirium in suvorexant group | Japan |
| Leung, J.M. (2017)^70^ | Scheduled for spine surgery or joint replacement surgery | Surgery | Gabapentin 900 mg Placebo | 350 347 | 73.0±6.0 73.0±6.0 | 55.1 45.5 | CAM-ICU | 84 72 | No statistically significant difference | USA |
| Li, X. (2017)^71^ | 60 years or older with elective coronary artery bypass graft and/or valve replacement surgery | Surgery | Dexmedetomidine 0.4-0.6 ug/kg/h Placebo | 142 143 | 66.4±5.4 67.5±5.3 | 33.1 28.7 | CAM-ICU | 7 11 | No statistically significant difference | China |
| Mu, D.L. (2017)^72^ | Scheduled for elective total hip or knee replacement surgery | Surgery | Parecoxib 40mg Placebo | 310 310 | 69.6±6.5 70.5±6.9 | 73.9 73.2 | CAM | 19 34 | lower occurrence of delirium in parecoxib group | China |
| Page, V.J. (2017)^73^ | Adult patients undergoing mechanical ventilation | ICU/Ward | Simvastatin 80mg/day Placebo | 71 71 | 61.9±15.3 62.1±17.3 | 36.6 47.9 | CAM-ICU | 66 67 | No statistically significant difference | UK |
| Royse, C.F. (2017)^74^ | Scheduled for cardiopulmonary bypass | Surgery | Methylprednisolone 250mg Placebo | 250 248 | 73.4±10.5 74.3±9.3 | 37.7 34.1 | CAM-ICU | 20 26 | No statistically significant difference | Multiple countries |
| Youn, Y.C. (2017)^75^ | Scheduled for surgical treatment after femoral neck or intertrochanter fracture | Surgery | Rivastigmine patch (4.6 mg) Standard of care | 31 31 | 79.4±6.3 79.2±5.8 | 61.3 54.8 | CAM | 5 14 | lower occurrence of delirium in rivastigmine group | Korea |
| Abdelgalel, E.F. (2016)^76^ | ICU noninvasive mechanical ventilation patients | ICU/Ward | Dexmedetomidine 0.2-0.7 ug/kg/h Haloperidol 2.5mg/day Placebo (normal saline) | 30 30 30 | 51.1±8.4 51.0±8.8 49.1±8.0 | 20.0 26.7 30.0 | CAM-ICU | 3 10 13 | lower occurrence of delirium in dexmedetomidine group | Egypt |
| Djaiani, G. (2016)^77^ | Scheduled for elective complex cardiac surgery | ICU/Ward | Dexmedetomidine 0.4 ug/kg/h Standard of care (propofol) | 91 92 | 72.7±6.4 72.4±6.2 | 25.3 24.0 | CAM | 16 29 | lower occurrence of delirium in dexmedetomidine group | Canada |
| Liu, Y. (2016) normal cognition^78^ | Scheduled for total hip joint or knee joint or shoulder joint replacement surgery with general anesthesia | Surgery | Dexmedetomidine 0.2-0.4 ug/kg/h Placebo (normal saline) | 60 58 | 71.2±8.1 72.8±9.2 | 56.7 50.0 | CAM | 5 18 | lower occurrence of delirium in dexmedetomidine group | China |
| Liu, Y. (2016) MCI^78^ | Scheduled for total hip joint or knee joint or shoulder joint replacement surgery with general anesthesia | Surgery | Dexmedetomidine 0.2-0.4 ug/kg/h Placebo (normal saline) | 39 40 | 72.8±8.2 75.3±7.8 | 53.8 42.5 | CAM | 10 25 | lower occurrence of delirium in dexmedetomidine group | China |
| Lu, X. (2016)^79^ | Critical ill under mechanical ventilation | ICU/Ward | Dexmedetomidine 0.2-1.0 mg/kg/h Standard of care (midazolam) | 40 40 | 64.2±9.9 62.5±9.6 | 55.0 47.5 | RASS | 8 18 | lower occurrence of delirium in dexmedetomidine group | China |
| Mohammadi, M. (2016)^80^ | Admitted to the ICU after noncardiac surgery | ICU/Ward | Cyproheptadine 4-mg TID Placebo | 20 20 | 59.5±17.9 59.9±12.9 | 60.0 70.0 | CAM-ICU | 3 7 | lower occurrence of delirium in cyproheptadine group | Iran |
| Su, X. (2016)^81^ | Scheduled for elective non-cardiac surgery under general anaesthesia | Surgery | Dexmedetomidine 200 ug Placebo (normal saline) | 350 350 | 74.3±6.7 74.4±7.0 | 36.9 42.3 | CAM-ICU | 32 79 | lower occurrence of delirium in dexmedetomidine group | China |
| Guo, Y. (2015)^82^ | Scheduled for oral cancer surgery | Surgery | Dexmedetomidine 0.2 ug/kg/h Placebo | 78 78 | 71.9±5.1 70.7±4.6 | 47.4 50.0 | CAM-ICU | 6 21 | lower occurrence of delirium in dexmedetomidine group | China |
| Song, R. (2015)^83^ | ICU ventilator-assisted therapy | ICU/Ward | Dexmedetomidine 0.2-0.7 ug/kg/h Standard of care (midazolam) | 42 48 | 46.3±12.9 45.4±10.1 | 35.7 52.1 | CAM-ICU | 4 15 | lower occurrence of delirium in dexmedetomidine group | China |
| Wang, K. (2015)^84^ | 60 years or older with spine surgery | ICU/Ward | Dexmedetomidine 3 ug/kg Standard of care (sufentanil) | 75 77 | 67.2±5.6 68.3±5.2 | 48.0 50.6 | CAM | 3 8 | No statistically significant difference | China |
| Yang, X. (2015)^85^ | Scheduled for selected maxillofacial surgery with microvascular free flap reconstruction | Surgery | Dexmedetomidine 0.5 ug/kg/h Placebo (normal saline) | 39 40 | 50.3±15.0 50.6±12.3 | 46.2 47.5 | CAM-ICU | 2 5 | No statistically significant difference | China |
| de Jonghe, A. (2014)^86^ | Emergent surgical treatment of hip fracture | Surgery | Melatonin 3mg/day Placebo | 186 192 | 84.1±8.0 83.4±7.5 | 71.5 67.7 | DSM-IV criteria | 55 49 | No statistically significant difference | the Netherlands |
| Dighe, K. (2014)^87^ | Scheduled for total knee arthroplasty | Surgery | Gabapentin 600 mg Placebo | 83 78 | 62.5±6.5 62.9±7.2 | 53.0 47.4 | CAM | 10 7 | No statistically significant difference | Canada |
| Hatta, K. (2014)^88^ | Admitted due to serious medical problems | ICU/Ward | Ramelteon 8 mg/day Placebo | 33 34 | 78.2±6.6 78.3±6.8 | 51.5 67.6 | DSM-IV criteria | 1 11 | lower occurrence of delirium in ramelteon group | Japan |
| Papadopoulos, G. (2014)^89^ | Scheduled for femoral or hip fracture rehabilitation surgery | Surgery | Ondansetron 8mg/day Placebo | 51 55 | 71.9±12.5 70.7±13.3 | NA | CAM | 18 29 | lower occurrence of delirium in ondansetron group | Greece |
| Pretto, G. (2014)^90^ | Major burn | ICU/Ward | Clonidine 0.5 ug/kg Placebo | 24 22 | 34.3±11.9 31.5±13.5 | 33.3 45.5 | Not mentioned | 7 5 | No statistically significant difference | Brazil |
| Robinson, T.N. (2014)^91^ | Scheduled for elective operation with a planned postoperative intensive care unit (ICU) admission | Surgery | L-tryptophan 3 g/day Placebo | 152 149 | 69.0±8.0 69.0±7.0 | 1.3 2.7 | CAM-ICU | 61 55 | No statistically significant difference | USA |
| Sauer, A.M. (2014)^92^ | Scheduled cardiac surgery with cardiopulmonary bypass | Surgery | Dexamethasone 1 mg/kg Placebo | 367 370 | 67.0±12.0 66.0±12.0 | 30.5 39.2 | CAM | 52 55 | No statistically significant difference | USA |
| Wang, W. (2012)^93^ | Admitted to the ICU after noncardiac surgery | ICU/Ward | Haloperidol 5 mg Placebo | 229 228 | 74.0±5.8 74.4±7.0 | 36.7 37.3 | RASS | 35 53 | lower occurrence of delirium in haloperidol group | China |
| Zaslavsky, A. (2012)^94^ | 65 years and older admitted for elective surgery | Surgery | Rivastigmine 3mg/day Placebo | 11 17 | 84.3±11.2 80.6±8.5 | 50.0 42.9 | CAM | 2 4 | lower occurrence of delirium in rivastigmine group | USA |
| Al-Aama, T. (2011)^95^ | Admitted through the emergency department to internal medicine ward | ICU/Ward | Melatonin 0.5mg/day Placebo | 61 61 | 84.3±5.9 84.6±6.2 | 54.1 60.7 | CAM | 7 19 | lower occurrence of delirium in melatonin group | Canada |
| Wan, L.J. (2011)^96^ | Critical ill under mechanical ventilation | ICU/Ward | Dexmedetomidine Standard of care (midazolam) | 102 98 | NA | 41.2 45.9 | SAS | 4 31 | lower occurrence of delirium in dexmedetomidine group | China |
| Larsen, K.A. (2010)^97^ | Scheduled for unilateral or bilateral joint-replacement surgery | Surgery | Olanzapine 5mg before surgery Placebo | 196 204 | 73.4±6.1 74.0±6.2 | 48.0 60.3 | CAM | 28 82 | lower occurrence of delirium in olanzapine group | USA |
| Rubino, A.S. (2010)^98^ | Scheduled for acute type-A aortic dissection | Surgery | Clonidine 0.5-2 ug/kg Placebo (normal saline) | 15 15 | 63.9±8.9 61.3±6.3 | 33.3 46.7 | DDS | 6 5 | No statistically significant difference | Italy |
| Gamberini, M. (2009)^99^ | Scheduled for elective cardiac surgery with cardiopulmonary bypass | Surgery | Rivastigmine 4.5mg/day Placebo | 56 57 | 74.1±5.2 74.4±5.9 | 33.9 29.8 | CAM | 18 17 | No statistically significant difference | Switzerland |
| Hudetz, J.A. (2009)^100^ | Scheduled for elective coronary artery bypass graft surgery or valve replacement/repair procedures | Surgery | Ketamine 0.5 mg/kg Placebo (normal saline) | 29 29 | 68.0±8.0 60.0±8.0 | NA | ICDS | 1 9 | lower occurrence of delirium in ketamine group | USA |
| Maldonado, J.R. (2009)^101^ | Elective cardiac valve operations | ICU/Ward | Dexmedetomidine 0.2-0.7 ug/kg/h Standard of care (midazolam) | 30 30 | 55.0±16.0 60.0±16.0 | 35.0 32.5 | CAM-ICU | 1 15 | lower occurrence of delirium in dexmedetomidine group | USA |
| Prakanrattana, U. (2007)^102^ | Scheduled for elective cardiac surgery with cadiopulmonary bypass | Surgery | Risperidone 1mg Placebo | 63 63 | 61.3±9.7 60.7±9.8 | 42.9 39.7 | CAM | 7 20 | lower occurrence of delirium in risperidone group | Thailand |
| Sampson, E.L. (2007)^103^ | Patients undergoing elective total hip replacement surgery | Surgery | Donepezil 5mg/day Placebo | 19 14 | 69.7±8.4 65.1±11.1 | 42.1 57.1 | DSI | 2 5 | lower occurrence of delirium in donepezil group | UK |
| Leung, J.M. (2006)^104^ | Scheduled for surgery involving the spine, requiring general anesthesia | Surgery | Gabapentin 900 mg Placebo | 9 12 | 57.2±10.3 61.4±11.3 | 55.6 41.7 | CAM | 0 5 | lower occurrence of delirium in gabapentin group | USA |
| Kalisvaart, K.J. (2005)^105^ | Admitted for acute or elective hip surgery | Surgery | Haloperidol 1.5 mg/day Placebo | 212 218 | 78.7±6.0 79.6±6.3 | 81.1 78.9 | CAM | 32 36 | No statistically significant difference | the Netherlands |
| Liptzin, B. (2005)^106^ | Scheduled for elective total knee or hip arthroplasty | Surgery | Donepezil 10mg/day Placebo | 39 41 | 66.8±8.9 67.6±8.6 | 64.1 51.2 | CAM | 8 7 | No statistically significant difference | USA |

^#^: there is discrepancy between its table and text. We extract data from main text because it was mostly consistent with the whole description throughout the manuscript

Abbreviation: 3D-CAM: 3-Minute Diagnostic Assessment for Delirium using the Confusion Assessment Method; 4AT: 4 'A's Test; AldS: Aldrete score; CAM: Confusion Assessment Method; CAM-ICU: Confusion Assessment Method for the ICU; DDS: Delirium Detection Score; DRS: Delirium Rating Scale; DSI: Delirium Symptom Interview; DSM: Diagnostic and Statistical Manual of Mental Disorders; Dx: diagnosis; ICDS: Intensive Care Delirium Screening Checklist; MCI: minimal cognition impairment; Nu-DESC: Nursing Delirium Screening Scale; RASS: Richmond Agitation and Sedation Score; RSA: Riker Sedation Agitation; SAS: Riker Sedation-Agitation Scale

**eTable 5A: League table of primary outcome: incidence of delirium**

| Suvorexant | . | . | . | . | . | . | . | . | . | . | . | . | . | . | . | . | . | . | . | . | . | 0.08 [0.00; 1.42] | . | . | . | . | . | . | . | . | . | . | . |
| --- | --- | --- | --- | --- | --- | --- | --- | --- | --- | --- | --- | --- | --- | --- | --- | --- | --- | --- | --- | --- | --- | --- | --- | --- | --- | --- | --- | --- | --- | --- | --- | --- | --- |
| 0.29 [0.01; 6.62] | Insulin | . | . | . | . | . | . | . | . | . | . | . | . | . | . | . | . | . | . | . | . | ***0.26 [0.09; 0.80]** | . | . | . | . | . | . | . | . | . | . | . |
| 0.22 [0.01; 4.42] | 0.74 [0.19; 2.85] | Olanzapine | . | . | . | . | . | . | . | . | . | . | . | . | . | . | . | . | . | . | . | ***0.36 [0.16; 0.77]** | . | . | . | . | . | . | . | . | . | . | . |
| 0.22 [0.01; 4.85] | 0.75 [0.17; 3.42] | 1.02 [0.28; 3.68] | Risperidone | . | . | . | . | . | . | . | . | . | . | . | . | . | . | . | . | . | . | ***0.35 [0.12; 0.98]** | . | . | . | . | . | . | . | . | . | . | . |
| 0.21 [0.01; 4.90] | 0.72 [0.14; 3.63] | 0.98 [0.24; 3.97] | 0.96 [0.20; 4.59] | Aripiprazole | . | . | . | . | . | . | . | . | . | . | . | . | . | . | . | . | . | 0.36 [0.11; 1.17] | . | . | . | . | . | . | . | . | . | . | . |
| 0.18 [0.01; 4.52] | 0.61 [0.11; 3.59] | 0.83 [0.17; 4.01] | 0.82 [0.15; 4.56] | 0.85 [0.14; 5.17] | Cyproheptadine | . | . | . | . | . | . | . | . | . | . | . | . | . | . | . | . | 0.43 [0.11; 1.70] | . | . | . | . | . | . | . | . | . | . | . |
| 0.14 [0.01; 2.77] | 0.49 [0.14; 1.67] | 0.66 [0.26; 1.68] | 0.65 [0.20; 2.08] | 0.68 [0.19; 2.45] | 0.80 [0.18; 3.48] | Methylprednisolone | . | . | . | . | . | . | 0.09 [0.01; 1.75] | . | . | . | . | . | . | . | . | ***0.58 [0.34; 0.99]** | . | . | . | . | . | . | . | . | . | . | . |
| 0.13 [0.01; 2.47] | 0.45 [0.15; 1.40] | 0.61 [0.28; 1.36] | 0.60 [0.21; 1.72] | 0.63 [0.19; 2.05] | 0.74 [0.18; 2.96] | 0.93 [0.53; 1.63] | Dexmedetomidine | . | . | . | 1.29 [0.32; 5.16] | . | . | . | . | . | . | . | . | 0.30 [0.08; 1.17] | . | ***0.57 [0.46; 0.70]** | . | . | . | . | . | . | 0.51 [0.20; 1.31] | . | . | . | ***0.37 [0.26; 0.53]** |
| 0.16 [0.01; 4.43] | 0.55 [0.08; 3.78] | 0.74 [0.13; 4.30] | 0.73 [0.11; 4.82] | 0.76 [0.11; 5.43] | 0.89 [0.11; 7.27] | 1.12 [0.21; 5.94] | 1.21 [0.25; 5.96] | Haloperidol_Ketamine | . | . | . | . | . | 0.68 [0.11; 4.40] | . | . | . | . | . | . | . | 0.48 [0.08; 2.83] | . | . | . | . | . | . | . | 0.39 [0.07; 2.19] | . | . | . |
| 0.14 [0.01; 2.88] | 0.47 [0.12; 1.91] | 0.64 [0.20; 2.01] | 0.63 [0.16; 2.40] | 0.65 [0.15; 2.78] | 0.77 [0.15; 3.88] | 0.96 [0.35; 2.64] | 1.04 [0.43; 2.51] | 0.86 [0.14; 5.20] | Parecoxib | . | . | . | . | . | . | . | . | . | . | . | . | 0.56 [0.24; 1.32] | . | . | . | . | . | . | . | . | . | . | . |
| 0.12 [0.01; 2.46] | 0.42 [0.11; 1.54] | 0.57 [0.20; 1.59] | 0.56 [0.16; 1.92] | 0.58 [0.15; 2.25] | 0.68 [0.15; 3.18] | 0.86 [0.36; 2.03] | 0.93 [0.46; 1.89] | 0.77 [0.14; 4.20] | 0.89 [0.30; 2.67] | Ketamine_low_dosage | . | . | . | 0.84 [0.39; 1.80] | . | . | . | . | . | . | . | 0.70 [0.34; 1.44] | . | . | . | . | . | . | . | . | . | . | . |
| 0.13 [0.01; 2.96] | 0.43 [0.08; 2.21] | 0.58 [0.14; 2.43] | 0.57 [0.12; 2.80] | 0.59 [0.11; 3.20] | 0.70 [0.11; 4.37] | 0.88 [0.24; 3.29] | 0.95 [0.29; 3.13] | 0.78 [0.11; 5.74] | 0.91 [0.21; 4.02] | 1.02 [0.26; 4.10] | Lidocaine | . | . | . | . | . | . | . | . | . | . | . | . | . | . | . | . | . | . | . | . | . | 0.44 [0.13; 1.56] |
| 0.11 [0.01; 2.37] | 0.39 [0.10; 1.54] | 0.53 [0.17; 1.62] | 0.52 [0.14; 1.94] | 0.54 [0.13; 2.25] | 0.64 [0.13; 3.15] | 0.80 [0.31; 2.10] | 0.87 [0.38; 1.99] | 0.72 [0.12; 4.23] | 0.83 [0.26; 2.71] | 0.94 [0.33; 2.68] | 0.91 [0.21; 3.90] | Ondansetron | . | . | . | . | . | . | . | . | . | 0.67 [0.30; 1.50] | . | . | . | . | . | . | . | . | . | . | . |
| 0.10 [0.01; 2.00] | 0.35 [0.10; 1.20] | 0.48 [0.19; 1.21] | 0.47 [0.15; 1.50] | 0.49 [0.14; 1.76] | 0.58 [0.13; 2.51] | 0.72 [0.35; 1.50] | 0.78 [0.45; 1.37] | 0.65 [0.12; 3.42] | 0.75 [0.28; 2.05] | 0.84 [0.36; 1.99] | 0.82 [0.22; 3.07] | 0.90 [0.35; 2.35] | Dexamethasone | . | . | . | . | . | . | . | . | 0.70 [0.41; 1.18] | . | . | . | . | . | . | . | . | . | . | . |
| 0.10 [0.01; 1.99] | 0.35 [0.10; 1.19] | 0.47 [0.19; 1.20] | 0.47 [0.15; 1.48] | 0.49 [0.13; 1.75] | 0.57 [0.13; 2.49] | 0.72 [0.34; 1.51] | 0.77 [0.44; 1.35] | 0.64 [0.13; 3.23] | 0.75 [0.27; 2.03] | 0.84 [0.42; 1.68] | 0.82 [0.22; 3.04] | 0.89 [0.34; 2.33] | 0.99 [0.47; 2.07] | Ketamine_high_dosage | . | . | . | . | . | . | . | 0.80 [0.47; 1.36] | . | . | . | . | . | . | . | 0.57 [0.12; 2.64] | . | . | . |
| 0.10 [0.00; 2.16] | 0.35 [0.08; 1.47] | 0.47 [0.14; 1.56] | 0.46 [0.11; 1.85] | 0.48 [0.11; 2.13] | 0.56 [0.11; 2.97] | 0.71 [0.24; 2.06] | 0.76 [0.29; 1.98] | 0.63 [0.10; 3.96] | 0.73 [0.21; 2.61] | 0.82 [0.26; 2.61] | 0.80 [0.18; 3.69] | 0.88 [0.26; 3.01] | 0.98 [0.34; 2.84] | 0.99 [0.34; 2.86] | Donepezil | . | . | . | . | . | . | 0.76 [0.30; 1.93] | . | . | . | . | . | . | . | . | . | . | . |
| 0.09 [0.00; 1.80] | 0.31 [0.09; 1.12] | 0.42 [0.15; 1.14] | 0.41 [0.12; 1.39] | 0.43 [0.11; 1.63] | 0.51 [0.11; 2.31] | 0.63 [0.28; 1.46] | 0.69 [0.36; 1.32] | 0.57 [0.10; 3.13] | 0.66 [0.23; 1.93] | 0.74 [0.29; 1.89] | 0.72 [0.19; 2.77] | 0.79 [0.28; 2.21] | 0.88 [0.38; 2.01] | 0.89 [0.39; 2.03] | 0.90 [0.29; 2.78] | Rivastigmine | . | . | . | . | . | 1.00 [0.47; 2.16] | . | . | . | . | . | . | . | . | . | . | 0.36 [0.12; 1.09] |
| 0.09 [0.00; 1.78] | 0.31 [0.09; 1.09] | 0.42 [0.16; 1.10] | 0.41 [0.13; 1.35] | 0.43 [0.11; 1.59] | 0.50 [0.11; 2.25] | 0.63 [0.29; 1.40] | 0.68 [0.36; 1.28] | 0.56 [0.10; 3.06] | 0.66 [0.23; 1.87] | 0.74 [0.30; 1.82] | 0.72 [0.19; 2.76] | 0.79 [0.29; 2.14] | 0.87 [0.40; 1.93] | 0.88 [0.40; 1.94] | 0.89 [0.30; 2.70] | 1.00 [0.41; 2.39] | Haloperidol_medium_dosage | . | . | . | . | 0.85 [0.47; 1.54] | . | . | . | . | . | . | . | . | . | . | . |
| 0.09 [0.00; 1.66] | ***0.30 [0.10; 0.95]** | ***0.41 [0.18; 0.94]** | 0.40 [0.14; 1.18] | 0.42 [0.12; 1.40] | 0.49 [0.12; 2.01] | 0.62 [0.33; 1.14] | ***0.67 [0.46; 0.97]** | 0.55 [0.11; 2.76] | 0.64 [0.26; 1.60] | 0.72 [0.34; 1.52] | 0.70 [0.20; 2.44] | 0.77 [0.32; 1.82] | 0.85 [0.46; 1.57] | 0.86 [0.47; 1.58] | 0.87 [0.33; 2.33] | 0.97 [0.47; 1.99] | 0.98 [0.50; 1.91] | Melatonin | . | . | . | 0.87 [0.64; 1.19] | . | . | . | . | . | . | . | . | . | . | . |
| 0.08 [0.00; 1.70] | 0.28 [0.07; 1.10] | 0.38 [0.13; 1.15] | 0.38 [0.10; 1.38] | 0.39 [0.10; 1.60] | 0.46 [0.10; 2.25] | 0.58 [0.23; 1.49] | 0.63 [0.28; 1.40] | 0.52 [0.09; 3.02] | 0.60 [0.19; 1.92] | 0.68 [0.24; 1.90] | 0.66 [0.16; 2.78] | 0.72 [0.24; 2.21] | 0.80 [0.32; 2.05] | 0.81 [0.32; 2.06] | 0.82 [0.25; 2.76] | 0.92 [0.33; 2.51] | 0.92 [0.35; 2.44] | 0.94 [0.41; 2.17] | Neostigmine | . | . | 0.92 [0.43; 2.00] | . | . | . | . | . | . | . | . | . | . | . |
| 0.08 [0.00; 1.54] | ***0.28 [0.09; 0.90]** | ***0.38 [0.16; 0.89]** | 0.37 [0.12; 1.11] | 0.38 [0.11; 1.32] | 0.45 [0.11; 1.89] | 0.57 [0.29; 1.10] | ***0.61 [0.40; 0.95]** | 0.51 [0.10; 2.54] | 0.59 [0.23; 1.52] | 0.66 [0.30; 1.45] | 0.65 [0.18; 2.30] | 0.71 [0.29; 1.73] | 0.78 [0.41; 1.51] | 0.79 [0.42; 1.51] | 0.80 [0.29; 2.20] | 0.89 [0.42; 1.90] | 0.90 [0.44; 1.83] | 0.92 [0.56; 1.52] | 0.98 [0.41; 2.32] | Haloperidol_high_dosage | . | 0.91 [0.61; 1.36] | . | . | . | . | . | . | . | 0.84 [0.42; 1.67] | . | . | . |
| 0.08 [0.00; 1.56] | ***0.27 [0.07; 0.98]** | 0.36 [0.13; 1.00] | 0.36 [0.10; 1.22] | 0.37 [0.10; 1.43] | 0.44 [0.09; 2.01] | 0.55 [0.23; 1.29] | 0.59 [0.29; 1.19] | 0.49 [0.09; 2.72] | 0.57 [0.19; 1.69] | 0.64 [0.24; 1.66] | 0.62 [0.16; 2.47] | 0.68 [0.24; 1.94] | 0.75 [0.32; 1.77] | 0.76 [0.32; 1.78] | 0.77 [0.24; 2.43] | 0.86 [0.34; 2.18] | 0.86 [0.35; 2.12] | 0.89 [0.42; 1.86] | 0.94 [0.34; 2.62] | 0.96 [0.44; 2.10] | Simvastatin | 0.99 [0.50; 1.93] | . | . | . | . | . | . | . | . | . | . | . |
| 0.08 [0.00; 1.42] | ***0.26 [0.09; 0.80]** | ***0.36 [0.16; 0.77]** | 0.35 [0.12; 0.98] | 0.36 [0.11; 1.17] | 0.43 [0.11; 1.70] | ***0.54 [0.32; 0.91]** | ***0.58 [0.48; 0.71]** | 0.48 [0.10; 2.34] | 0.56 [0.24; 1.32] | 0.63 [0.32; 1.24] | 0.61 [0.18; 2.05] | 0.67 [0.30; 1.50] | 0.74 [0.44; 1.25] | 0.75 [0.45; 1.26] | 0.76 [0.30; 1.93] | 0.85 [0.44; 1.61] | 0.85 [0.47; 1.54] | 0.87 [0.64; 1.19] | 0.92 [0.43; 2.00] | 0.95 [0.64; 1.40] | 0.99 [0.50; 1.93] | Placebo | 0.97 [0.54; 1.74] | 0.92 [0.44; 1.90] | . | 0.85 [0.27; 2.64] | 0.92 [0.50; 1.66] | 0.62 [0.18; 2.18] | 0.81 [0.35; 1.85] | 0.83 [0.45; 1.54] | 0.76 [0.33; 1.79] | 0.57 [0.12; 2.69] | . |
| 0.07 [0.00; 1.46] | ***0.26 [0.07; 0.89]** | ***0.35 [0.13; 0.91]** | 0.34 [0.10; 1.11] | 0.35 [0.10; 1.31] | 0.42 [0.09; 1.85] | 0.52 [0.24; 1.15] | 0.56 [0.30; 1.04] | 0.47 [0.09; 2.52] | 0.54 [0.19; 1.53] | 0.61 [0.25; 1.49] | 0.59 [0.16; 2.27] | 0.65 [0.24; 1.76] | 0.72 [0.33; 1.58] | 0.73 [0.33; 1.59] | 0.74 [0.25; 2.21] | 0.82 [0.35; 1.96] | 0.83 [0.36; 1.90] | 0.85 [0.44; 1.64] | 0.90 [0.34; 2.36] | 0.92 [0.46; 1.86] | 0.96 [0.39; 2.33] | 0.97 [0.54; 1.74] | Ramelteon | . | . | . | . | . | . | . | . | . | . |
| 0.07 [0.00; 1.43] | ***0.24 [0.06; 0.91]** | ***0.33 [0.11; 0.94]** | 0.32 [0.09; 1.14] | 0.33 [0.08; 1.33] | 0.39 [0.08; 1.87] | 0.49 [0.20; 1.22] | 0.53 [0.25; 1.14] | 0.44 [0.08; 2.52] | 0.51 [0.17; 1.58] | 0.58 [0.21; 1.56] | 0.56 [0.14; 2.30] | 0.62 [0.21; 1.82] | 0.68 [0.28; 1.67] | 0.69 [0.28; 1.69] | 0.70 [0.21; 2.28] | 0.78 [0.30; 2.06] | 0.78 [0.31; 2.00] | 0.80 [0.36; 1.77] | 0.85 [0.29; 2.46] | 0.87 [0.38; 1.99] | 0.91 [0.34; 2.44] | 0.92 [0.44; 1.90] | 0.95 [0.37; 2.40] | L_tryptophan | . | . | . | . | . | . | . | . | . |
| 0.07 [0.00; 1.51] | 0.23 [0.05; 1.09] | 0.31 [0.08; 1.18] | 0.30 [0.07; 1.37] | 0.32 [0.06; 1.57] | 0.37 [0.06; 2.17] | 0.47 [0.14; 1.58] | 0.51 [0.17; 1.49] | 0.42 [0.06; 2.86] | 0.49 [0.12; 1.96] | 0.55 [0.15; 1.98] | 0.53 [0.11; 2.55] | 0.58 [0.15; 2.27] | 0.65 [0.19; 2.18] | 0.65 [0.19; 2.20] | 0.66 [0.16; 2.79] | 0.74 [0.21; 2.53] | 0.74 [0.21; 2.58] | 0.76 [0.24; 2.37] | 0.80 [0.21; 3.08] | 0.82 [0.26; 2.64] | 0.86 [0.24; 3.10] | 0.87 [0.29; 2.61] | 0.90 [0.26; 3.10] | 0.95 [0.25; 3.53] | Flurbiprofen_sufentanil | . | . | . | . | . | . | . | 0.69 [0.25; 1.94] |
| 0.07 [0.00; 1.49] | 0.22 [0.05; 1.09] | 0.30 [0.08; 1.19] | 0.30 [0.06; 1.38] | 0.31 [0.06; 1.58] | 0.36 [0.06; 2.16] | 0.46 [0.13; 1.60] | 0.49 [0.16; 1.56] | 0.41 [0.06; 2.86] | 0.47 [0.11; 1.97] | 0.53 [0.14; 2.00] | 0.52 [0.10; 2.72] | 0.57 [0.14; 2.29] | 0.63 [0.18; 2.20] | 0.64 [0.18; 2.22] | 0.65 [0.15; 2.80] | 0.72 [0.19; 2.65] | 0.72 [0.20; 2.60] | 0.74 [0.23; 2.40] | 0.78 [0.20; 3.10] | 0.80 [0.24; 2.67] | 0.84 [0.22; 3.13] | 0.85 [0.27; 2.64] | 0.87 [0.24; 3.13] | 0.92 [0.24; 3.55] | 0.97 [0.20; 4.73] | Thiamine | . | . | . | . | . | . | . |
| 0.07 [0.00; 1.38] | ***0.24 [0.07; 0.85]** | ***0.33 [0.12; 0.86]** | 0.32 [0.10; 1.06] | 0.33 [0.09; 1.24] | 0.39 [0.09; 1.76] | 0.49 [0.22; 1.09] | 0.53 [0.28; 1.00] | 0.44 [0.08; 2.39] | 0.51 [0.18; 1.46] | 0.57 [0.23; 1.42] | 0.56 [0.15; 2.15] | 0.61 [0.23; 1.67] | 0.68 [0.31; 1.50] | 0.69 [0.31; 1.52] | 0.70 [0.23; 2.10] | 0.78 [0.32; 1.87] | 0.78 [0.34; 1.81] | 0.80 [0.41; 1.57] | 0.85 [0.32; 2.25] | 0.87 [0.43; 1.77] | 0.90 [0.37; 2.22] | 0.92 [0.50; 1.66] | 0.94 [0.41; 2.17] | 1.00 [0.39; 2.55] | 1.05 [0.30; 3.67] | 1.08 [0.30; 3.90] | Gabapentin | . | . | . | . | . | . |
| 0.07 [0.00; 1.38] | ***0.23 [0.06; 0.88]** | ***0.31 [0.11; 0.91]** | 0.31 [0.09; 1.10] | 0.32 [0.08; 1.28] | 0.38 [0.08; 1.80] | 0.48 [0.19; 1.18] | 0.51 [0.25; 1.07] | 0.42 [0.07; 2.43] | 0.49 [0.16; 1.53] | 0.55 [0.20; 1.51] | 0.54 [0.14; 2.11] | 0.59 [0.20; 1.76] | 0.66 [0.27; 1.62] | 0.66 [0.27; 1.63] | 0.67 [0.21; 2.20] | 0.75 [0.29; 1.93] | 0.75 [0.29; 1.94] | 0.77 [0.35; 1.72] | 0.82 [0.28; 2.38] | 0.84 [0.36; 1.93] | 0.87 [0.32; 2.36] | 0.88 [0.42; 1.85] | 0.91 [0.36; 2.33] | 0.96 [0.34; 2.71] | 1.02 [0.29; 3.52] | 1.04 [0.27; 4.03] | 0.96 [0.37; 2.49] | Quetiapine | . | . | . | . | 0.59 [0.26; 1.34] |
| 0.06 [0.00; 1.28] | **0.22 [0.06; 0.79]** | ***0.30 [0.11; 0.81]** | ***0.29 [0.09; 0.99]** | 0.31 [0.08; 1.16] | 0.36 [0.08; 1.63] | 0.45 [0.20; 1.03] | ***0.49 [0.26; 0.92]** | 0.40 [0.07; 2.21] | 0.47 [0.16; 1.36] | 0.53 [0.21; 1.33] | 0.51 [0.13; 1.98] | 0.56 [0.20; 1.56] | 0.62 [0.28; 1.41] | 0.63 [0.28; 1.43] | 0.64 [0.21; 1.96] | 0.71 [0.29; 1.74] | 0.71 [0.30; 1.70] | 0.73 [0.36; 1.48] | 0.78 [0.29; 2.11] | 0.80 [0.38; 1.67] | 0.83 [0.33; 2.08] | 0.84 [0.45; 1.58] | 0.87 [0.37; 2.04] | 0.91 [0.35; 2.39] | 0.97 [0.28; 3.38] | 0.99 [0.27; 3.63] | 0.92 [0.39; 2.18] | 0.95 [0.37; 2.48] | Clonidine | . | . | . | . |
| 0.07 [0.00; 1.27] | ***0.22 [0.06; 0.77]** | ***0.30 [0.12; 0.78]** | ***0.30 [0.09; 0.96]** | 0.31 [0.08; 1.13] | 0.36 [0.08; 1.60] | ***0.45 [0.21; 0.98]** | ***0.49 [0.27; 0.89]** | 0.41 [0.08; 2.00] | 0.47 [0.17; 1.32] | 0.53 [0.22; 1.26] | 0.52 [0.14; 1.96] | 0.57 [0.21; 1.51] | 0.63 [0.29; 1.35] | 0.63 [0.30; 1.32] | 0.64 [0.22; 1.91] | 0.72 [0.30; 1.68] | 0.72 [0.32; 1.63] | 0.74 [0.39; 1.40] | 0.78 [0.30; 2.03] | 0.80 [0.45; 1.44] | 0.83 [0.35; 2.00] | 0.85 [0.48; 1.48] | 0.87 [0.39; 1.96] | 0.92 [0.37; 2.30] | 0.97 [0.28; 3.33] | 1.00 [0.28; 3.54] | 0.92 [0.41; 2.09] | 0.96 [0.38; 2.42] | 1.01 [0.43; 2.34] | Haloperidol_low_dosage | . | . | . |
| 0.06 [0.00; 1.23] | ***0.20 [0.05; 0.81]** | ***0.27 [0.09; 0.86]** | 0.27 [0.07; 1.02] | 0.28 [0.07; 1.18] | 0.33 [0.06; 1.65] | 0.41 [0.15; 1.12] | 0.44 [0.18; 1.07] | 0.37 [0.06; 2.21] | 0.43 [0.13; 1.43] | 0.48 [0.16; 1.42] | 0.47 [0.11; 2.05] | 0.51 [0.16; 1.65] | 0.57 [0.21; 1.54] | 0.57 [0.21; 1.55] | 0.58 [0.16; 2.05] | 0.65 [0.22; 1.88] | 0.65 [0.23; 1.84] | 0.67 [0.27; 1.65] | 0.71 [0.22; 2.23] | 0.72 [0.28; 1.85] | 0.75 [0.25; 2.23] | 0.76 [0.33; 1.79] | 0.79 [0.28; 2.21] | 0.83 [0.27; 2.55] | 0.88 [0.22; 3.52] | 0.90 [0.22; 3.72] | 0.83 [0.29; 2.36] | 0.86 [0.28; 2.66] | 0.91 [0.31; 2.62] | 0.90 [0.32; 2.51] | Physostigmine | . | . |
| 0.04 [0.00; 1.19] | 0.15 [0.02; 1.01] | 0.20 [0.04; 1.14] | 0.20 [0.03; 1.28] | 0.21 [0.03; 1.45] | 0.24 [0.03; 1.94] | 0.31 [0.06; 1.58] | 0.33 [0.07; 1.58] | 0.27 [0.03; 2.51] | 0.32 [0.05; 1.87] | 0.36 [0.07; 1.94] | 0.35 [0.05; 2.49] | 0.38 [0.07; 2.19] | 0.42 [0.08; 2.18] | 0.43 [0.08; 2.19] | 0.43 [0.07; 2.64] | 0.48 [0.09; 2.59] | 0.48 [0.09; 2.55] | 0.50 [0.10; 2.42] | 0.53 [0.09; 2.98] | 0.54 [0.11; 2.67] | 0.56 [0.10; 3.04] | 0.57 [0.12; 2.69] | 0.59 [0.11; 3.07] | 0.62 [0.11; 3.43] | 0.65 [0.10; 4.37] | 0.67 [0.10; 4.59] | 0.62 [0.12; 3.27] | 0.64 [0.12; 3.59] | 0.68 [0.13; 3.61] | 0.67 [0.13; 3.51] | 0.74 [0.13; 4.38] | Minocycline | . |
| ***0.05 [0.00; 0.88]** | ***0.16 [0.05; 0.51]** | ***0.21 [0.09; 0.50]** | ***0.21 [0.07; 0.63]** | ***0.22 [0.06; 0.75]** | 0.26 [0.06; 1.08] | ***0.32 [0.17; 0.62]** | ***0.35 [0.25; 0.49]** | 0.29 [0.06; 1.47] | ***0.34 [0.13; 0.86]** | ***0.38 [0.17; 0.82]** | 0.37 [0.11; 1.20] | ***0.40 [0.17; 0.98]** | ***0.45 [0.24; 0.85]** | ***0.45 [0.24; 0.86]** | 0.46 [0.17; 1.25] | 0.51 [0.26; 1.01] | 0.51 [0.25; 1.04] | ***0.53 [0.32; 0.86]** | 0.56 [0.24; 1.32] | ***0.57 [0.33; 0.98]** | 0.59 [0.27; 1.29] | ***0.60 [0.41; 0.88]** | 0.62 [0.31; 1.24] | 0.66 [0.29; 1.49] | 0.69 [0.25; 1.94] | 0.71 [0.21; 2.35] | 0.66 [0.32; 1.33] | 0.68 [0.34; 1.37] | 0.72 [0.35; 1.46] | 0.71 [0.36; 1.40] | 0.79 [0.31; 2.01] | 1.06 [0.21; 5.24] | Control |

Pairwise (upper-right portion) and network (lower-left portion) meta-analysis results are presented as estimate effect sizes for the outcome of incidence of delirium. Interventions are reported in order of mean ranking of incidence of delirium, and outcomes are expressed as risk ratio (RR) (95% confidence intervals). For the pairwise meta-analyses, RR of less than 1 indicate that the treatment specified in the row got less incidence of delirium than that specified in the column. For the network meta-analysis (NMA), RR of less than 1 indicate that the treatment specified in the column got less incidence of delirium than that specified in the row. Bold results marked with * indicate statistical significance.

**eTable 5B:** **League table of primary outcome: incidence of delirium: subgroup of ICU/ward setting**

| Ramelteon | . | . | . | . | . | . | . | . | ***0.09 [0.01; 0.75]** | . | . | . | . |
| --- | --- | --- | --- | --- | --- | --- | --- | --- | --- | --- | --- | --- | --- |
| 1.22 [0.03; 43.23] | Suvorexant | . | . | . | . | . | . | . | 0.08 [0.00; 1.40] | . | . | . | . |
| 0.26 [0.02; 2.75] | 0.21 [0.01; 4.77] | Aripiprazole | . | . | . | . | . | . | 0.36 [0.12; 1.13] | . | . | . | . |
| 0.22 [0.02; 2.60] | 0.18 [0.01; 4.39] | 0.85 [0.15; 4.92] | Cyproheptadine | . | . | . | . | . | 0.43 [0.11; 1.64] | . | . | . | . |
| 0.16 [0.02; 1.49] | 0.13 [0.01; 2.68] | 0.63 [0.16; 2.49] | 0.74 [0.16; 3.50] | Ketamine_high_dosage | . | . | . | . | 0.58 [0.26; 1.27] | . | . | . | . |
| 0.13 [0.02; 1.10] | 0.11 [0.01; 2.04] | 0.51 [0.15; 1.71] | 0.61 [0.15; 2.46] | 0.82 [0.34; 1.99] | Dexmedetomidine | . | 0.30 [0.08; 1.13] | . | 0.69 [0.45; 1.05] | . | . | . | ***0.37 [0.26; 0.54]** |
| ***0.11 [0.01; 0.88]** | 0.09 [0.00; 1.64] | 0.42 [0.13; 1.37] | 0.49 [0.12; 1.97] | 0.66 [0.28; 1.58] | 0.81 [0.47; 1.40] | Melatonin | . | . | 0.87 [0.61; 1.26] | . | . | . | . |
| ***0.10 [0.01; 0.82]** | 0.08 [0.00; 1.51] | 0.38 [0.12; 1.26] | 0.45 [0.11; 1.82] | 0.61 [0.26; 1.46] | 0.75 [0.44; 1.28] | 0.92 [0.55; 1.55] | Haloperidol_high_dosage | . | 0.91 [0.63; 1.32] | . | 0.84 [0.45; 1.56] | . | . |
| ***0.10 [0.01; 0.83]** | 0.08 [0.00; 1.51] | 0.37 [0.10; 1.33] | 0.44 [0.10; 1.90] | 0.59 [0.22; 1.59] | 0.72 [0.35; 1.49] | 0.89 [0.44; 1.80] | 0.96 [0.47; 1.95] | Simvastatin | 0.99 [0.54; 1.80] | . | . | . | . |
| ***0.09 [0.01; 0.75]** | 0.08 [0.00; 1.40] | 0.36 [0.12; 1.13] | 0.43 [0.11; 1.64] | 0.58 [0.26; 1.27] | 0.71 [0.47; 1.06] | 0.87 [0.61; 1.26] | 0.95 [0.66; 1.37] | 0.99 [0.54; 1.80] | Placebo | 0.78 [0.24; 2.48] | 0.83 [0.45; 1.54] | 0.62 [0.19; 2.10] | . |
| ***0.07 [0.01; 0.79]** | 0.06 [0.00; 1.36] | 0.28 [0.06; 1.43] | 0.33 [0.06; 1.97] | 0.45 [0.11; 1.83] | 0.55 [0.16; 1.88] | 0.68 [0.20; 2.29] | 0.74 [0.22; 2.49] | 0.77 [0.21; 2.83] | 0.78 [0.24; 2.48] | Clonidine | . | . | . |
| ***0.08 [0.01; 0.69]** | 0.07 [0.00; 1.27] | 0.31 [0.09; 1.11] | 0.37 [0.09; 1.58] | 0.50 [0.19; 1.31] | 0.61 [0.30; 1.22] | 0.75 [0.38; 1.47] | 0.81 [0.46; 1.44] | 0.84 [0.37; 1.94] | 0.86 [0.49; 1.52] | 1.10 [0.30; 4.00] | Haloperidol_low_dosage | . | . |
| ***0.07 [0.01; 0.68]** | 0.06 [0.00; 1.23] | 0.29 [0.07; 1.13] | 0.34 [0.07; 1.59] | 0.46 [0.16; 1.37] | 0.56 [0.28; 1.15] | 0.70 [0.30; 1.60] | 0.75 [0.33; 1.73] | 0.78 [0.30; 2.05] | 0.80 [0.38; 1.68] | 1.02 [0.26; 4.06] | 0.93 [0.36; 2.37] | Quetiapine | 0.59 [0.27; 1.26] |
| ***0.05 [0.01; 0.41]** | ***0.04 [0.00; 0.75]** | ***0.19 [0.05; 0.65]** | ***0.22 [0.05; 0.93]** | ***0.30 [0.12; 0.77]** | ***0.36 [0.25; 0.52]** | ***0.45 [0.24; 0.85]** | ***0.49 [0.26; 0.91]** | 0.51 [0.23; 1.13] | ***0.51 [0.30; 0.87]** | 0.66 [0.19; 2.35] | 0.60 [0.28; 1.29] | 0.65 [0.33; 1.26] | Control |

Pairwise (upper-right portion) and network (lower-left portion) meta-analysis results are presented as estimate effect sizes for the outcome of incidence of delirium. Interventions are reported in order of mean ranking of incidence of delirium, and outcomes are expressed as risk ratio (RR) (95% confidence intervals). For the pairwise meta-analyses, RR of less than 1 indicate that the treatment specified in the row got less incidence of delirium than that specified in the column. For the network meta-analysis (NMA), RR of less than 1 indicate that the treatment specified in the column got less incidence of delirium than that specified in the row. Bold results marked with * indicate statistical significance.

**eTable 5C: League table of primary outcome: incidence of delirium: subgroup of surgery setting**

| Insulin | . | . | . | . | . | . | . | . | . | . | . | . | . | . | . | . | . | ***0.26 [0.08; 0.85]** | . | . | . | . | . | . | . | . | . | . |
| --- | --- | --- | --- | --- | --- | --- | --- | --- | --- | --- | --- | --- | --- | --- | --- | --- | --- | --- | --- | --- | --- | --- | --- | --- | --- | --- | --- | --- |
| 0.74 [0.17; 3.16] | Olanzapine | . | . | . | . | . | . | . | . | . | . | . | . | . | . | . | . | ***0.36 [0.15; 0.84]** | . | . | . | . | . | . | . | . | . | . |
| 0.75 [0.15; 3.74] | 1.02 [0.25; 4.09] | Risperidone | . | . | . | . | . | . | . | . | . | . | . | . | . | . | . | 0.35 [0.12; 1.05] | . | . | . | . | . | . | . | . | . | . |
| 0.49 [0.15; 1.63] | 0.67 [0.27; 1.62] | 0.66 [0.21; 2.02] | Dexmedetomidine | . | . | . | 1.29 [0.31; 5.43] | . | . | . | . | . | . | . | . | . | 0.30 [0.07; 1.23] | ***0.52 [0.41; 0.67]** | . | . | . | 0.51 [0.19; 1.41] | . | . | . | . | . | ***0.40 [0.17; 0.98]** |
| 0.50 [0.14; 1.82] | 0.67 [0.24; 1.88] | 0.66 [0.19; 2.28] | 1.01 [0.54; 1.87] | Methylprednisolone | . | . | . | . | . | 0.09 [0.00; 1.80] | . | . | . | . | . | . | . | 0.57 [0.32; 1.03] | . | . | . | . | . | . | . | . | . | . |
| 0.47 [0.11; 2.11] | 0.64 [0.18; 2.27] | 0.63 [0.15; 2.66] | 0.95 [0.36; 2.51] | 0.95 [0.32; 2.85] | Parecoxib | . | . | . | . | . | . | . | . | . | . | . | . | 0.56 [0.22; 1.43] | . | . | . | . | . | . | . | . | . | . |
| 0.50 [0.06; 3.94] | 0.68 [0.10; 4.54] | 0.67 [0.09; 5.04] | 1.01 [0.18; 5.65] | 1.01 [0.17; 6.07] | 1.06 [0.15; 7.41] | Haloperidol_Ketamine | . | . | . | . | . | . | . | . | 0.68 [0.10; 4.57] | . | . | 0.48 [0.08; 2.94] | . | . | . | . | . | 0.39 [0.07; 2.28] | . | . | . | . |
| 0.45 [0.08; 2.65] | 0.61 [0.13; 2.96] | 0.60 [0.11; 3.37] | 0.92 [0.25; 3.39] | 0.92 [0.22; 3.86] | 0.96 [0.19; 4.87] | 0.91 [0.11; 7.81] | Lidocaine | . | . | . | . | . | . | . | . | . | . | . | . | . | . | . | . | . | . | . | . | 0.44 [0.12; 1.65] |
| 0.41 [0.10; 1.65] | 0.55 [0.17; 1.74] | 0.54 [0.14; 2.07] | 0.82 [0.36; 1.86] | 0.82 [0.31; 2.14] | 0.86 [0.25; 2.91] | 0.81 [0.13; 4.95] | 0.89 [0.19; 4.13] | Ketamine_low_dosage | . | . | . | . | . | . | 0.84 [0.36; 1.97] | . | . | 0.67 [0.30; 1.48] | . | . | . | . | . | . | . | . | . | . |
| 0.39 [0.09; 1.71] | 0.53 [0.15; 1.83] | 0.52 [0.13; 2.15] | 0.80 [0.32; 2.00] | 0.79 [0.28; 2.28] | 0.83 [0.23; 3.04] | 0.79 [0.12; 5.35] | 0.87 [0.18; 4.25] | 0.97 [0.30; 3.16] | Ondansetron | . | . | . | . | . | . | . | . | 0.67 [0.28; 1.63] | . | . | . | . | . | . | . | . | . | . |
| 0.36 [0.10; 1.30] | 0.48 [0.17; 1.34] | 0.47 [0.14; 1.63] | 0.72 [0.39; 1.33] | 0.72 [0.33; 1.58] | 0.75 [0.25; 2.26] | 0.71 [0.12; 4.26] | 0.78 [0.19; 3.29] | 0.88 [0.33; 2.30] | 0.90 [0.31; 2.59] | Dexamethasone | . | . | . | . | . | . | . | 0.69 [0.38; 1.22] | . | . | . | . | . | . | . | . | . | . |
| 0.35 [0.08; 1.61] | 0.48 [0.13; 1.74] | 0.47 [0.11; 2.03] | 0.71 [0.26; 1.94] | 0.71 [0.23; 2.19] | 0.75 [0.19; 2.88] | 0.70 [0.10; 4.99] | 0.77 [0.15; 3.99] | 0.87 [0.25; 3.02] | 0.89 [0.24; 3.34] | 0.99 [0.32; 3.05] | Donepezil | . | . | . | . | . | . | 0.75 [0.28; 1.98] | . | . | . | . | . | . | . | . | . | . |
| 0.32 [0.08; 1.25] | 0.43 [0.14; 1.31] | 0.42 [0.11; 1.57] | 0.64 [0.30; 1.35] | 0.64 [0.25; 1.60] | 0.67 [0.21; 2.19] | 0.63 [0.10; 4.01] | 0.70 [0.17; 2.87] | 0.78 [0.27; 2.26] | 0.80 [0.26; 2.53] | 0.89 [0.36; 2.23] | 0.90 [0.27; 3.02] | Rivastigmine | . | . | . | . | . | 1.00 [0.44; 2.28] | . | . | . | . | . | . | . | . | . | 0.36 [0.11; 1.16] |
| 0.31 [0.09; 1.15] | 0.42 [0.15; 1.18] | 0.42 [0.12; 1.43] | 0.63 [0.34; 1.18] | 0.63 [0.28; 1.41] | 0.66 [0.22; 1.99] | 0.63 [0.10; 3.76] | 0.69 [0.16; 2.90] | 0.77 [0.29; 2.03] | 0.80 [0.28; 2.28] | 0.88 [0.39; 1.97] | 0.89 [0.29; 2.74] | 0.99 [0.39; 2.48] | Melatonin | . | . | . | . | 0.84 [0.48; 1.48] | . | . | . | . | . | . | . | . | . | . |
| 0.31 [0.08; 1.18] | 0.42 [0.14; 1.23] | 0.41 [0.11; 1.48] | 0.63 [0.31; 1.26] | 0.62 [0.26; 1.49] | 0.66 [0.21; 2.06] | 0.62 [0.10; 3.83] | 0.68 [0.16; 2.98] | 0.77 [0.28; 2.11] | 0.79 [0.26; 2.37] | 0.87 [0.37; 2.07] | 0.88 [0.27; 2.84] | 0.98 [0.37; 2.60] | 0.99 [0.42; 2.35] | Haloperidol_medium_dosage | . | . | . | 0.85 [0.44; 1.63] | . | . | . | . | . | . | . | . | . | . |
| 0.30 [0.08; 1.21] | 0.41 [0.13; 1.27] | 0.40 [0.11; 1.51] | 0.61 [0.28; 1.34] | 0.61 [0.24; 1.55] | 0.64 [0.19; 2.12] | 0.60 [0.11; 3.37] | 0.67 [0.15; 3.03] | 0.75 [0.33; 1.68] | 0.77 [0.24; 2.45] | 0.85 [0.33; 2.17] | 0.86 [0.25; 2.92] | 0.96 [0.34; 2.70] | 0.97 [0.38; 2.46] | 0.97 [0.36; 2.62] | Ketamine_high_dosage | . | . | 0.97 [0.46; 2.07] | . | . | . | . | . | 0.57 [0.12; 2.77] | . | . | . | . |
| 0.28 [0.07; 1.22] | 0.38 [0.11; 1.30] | 0.38 [0.09; 1.53] | 0.58 [0.24; 1.41] | 0.57 [0.20; 1.61] | 0.60 [0.17; 2.16] | 0.57 [0.08; 3.83] | 0.63 [0.13; 3.03] | 0.70 [0.22; 2.24] | 0.72 [0.21; 2.50] | 0.80 [0.29; 2.25] | 0.81 [0.22; 2.97] | 0.90 [0.29; 2.77] | 0.91 [0.32; 2.55] | 0.92 [0.31; 2.71] | 0.94 [0.30; 2.94] | Neostigmine | . | 0.92 [0.39; 2.19] | . | . | . | . | . | . | . | . | . | . |
| 0.28 [0.06; 1.26] | 0.38 [0.10; 1.36] | 0.37 [0.09; 1.59] | 0.57 [0.22; 1.49] | 0.56 [0.19; 1.71] | 0.59 [0.16; 2.26] | 0.56 [0.08; 3.92] | 0.62 [0.12; 3.11] | 0.69 [0.20; 2.36] | 0.71 [0.19; 2.61] | 0.79 [0.26; 2.38] | 0.79 [0.20; 3.09] | 0.88 [0.27; 2.91] | 0.89 [0.29; 2.70] | 0.90 [0.28; 2.86] | 0.93 [0.28; 3.09] | 0.98 [0.27; 3.54] | Haloperidol_high_dosage | 0.77 [0.28; 2.10] | . | . | . | . | . | . | . | . | . | . |
| ***0.26 [0.08; 0.85]** | ***0.36 [0.15; 0.84]** | 0.35 [0.12; 1.05] | ***0.53 [0.42; 0.68]** | ***0.53 [0.30; 0.94]** | 0.56 [0.22; 1.43] | 0.53 [0.10; 2.88] | 0.58 [0.15; 2.17] | 0.65 [0.30; 1.42] | 0.67 [0.28; 1.63] | 0.74 [0.42; 1.31] | 0.75 [0.28; 1.98] | 0.83 [0.40; 1.72] | 0.84 [0.48; 1.48] | 0.85 [0.44; 1.63] | 0.87 [0.41; 1.83] | 0.92 [0.39; 2.19] | 0.94 [0.36; 2.44] | Placebo | . | 0.92 [0.41; 2.09] | 0.93 [0.48; 1.79] | 0.83 [0.25; 2.82] | 0.85 [0.26; 2.81] | 0.82 [0.19; 3.54] | 0.76 [0.30; 1.94] | 0.78 [0.41; 1.49] | 0.57 [0.11; 2.81] | . |
| 0.23 [0.04; 1.39] | 0.32 [0.06; 1.56] | 0.31 [0.05; 1.77] | 0.48 [0.13; 1.80] | 0.47 [0.11; 2.03] | 0.50 [0.10; 2.56] | 0.47 [0.05; 4.09] | 0.52 [0.10; 2.74] | 0.58 [0.12; 2.74] | 0.60 [0.12; 2.99] | 0.66 [0.15; 2.84] | 0.67 [0.13; 3.50] | 0.74 [0.19; 2.98] | 0.75 [0.17; 3.22] | 0.76 [0.17; 3.37] | 0.78 [0.17; 3.61] | 0.82 [0.17; 4.06] | 0.84 [0.16; 4.33] | 0.89 [0.23; 3.41] | Flurbiprofen_sufentanil | . | . | . | . | . | . | . | . | 0.69 [0.23; 2.07] |
| 0.24 [0.06; 1.01] | 0.33 [0.10; 1.07] | 0.32 [0.08; 1.27] | 0.49 [0.21; 1.15] | 0.49 [0.18; 1.32] | 0.51 [0.15; 1.79] | 0.48 [0.07; 3.20] | 0.53 [0.11; 2.52] | 0.60 [0.19; 1.85] | 0.62 [0.18; 2.06] | 0.68 [0.25; 1.85] | 0.69 [0.19; 2.45] | 0.77 [0.26; 2.28] | 0.77 [0.29; 2.10] | 0.78 [0.27; 2.23] | 0.80 [0.27; 2.42] | 0.85 [0.26; 2.79] | 0.87 [0.25; 3.04] | 0.92 [0.41; 2.09] | 1.03 [0.21; 4.98] | L_tryptophan | . | . | . | . | . | . | . | . |
| ***0.24 [0.06; 0.94]** | ***0.33 [0.11; 0.97]** | 0.33 [0.09; 1.17] | 0.50 [0.25; 1.00] | 0.49 [0.21; 1.18] | 0.52 [0.17; 1.63] | 0.49 [0.08; 3.03] | 0.54 [0.12; 2.36] | 0.60 [0.22; 1.67] | 0.62 [0.21; 1.88] | 0.69 [0.29; 1.64] | 0.70 [0.22; 2.25] | 0.77 [0.29; 2.06] | 0.78 [0.33; 1.86] | 0.79 [0.31; 1.99] | 0.81 [0.30; 2.19] | 0.86 [0.29; 2.54] | 0.88 [0.28; 2.78] | 0.93 [0.48; 1.79] | 1.04 [0.23; 4.65] | 1.01 [0.35; 2.89] | Gabapentin | . | . | . | . | . | . | . |
| ***0.24 [0.06; 0.98]** | 0.32 [0.10; 1.04] | 0.32 [0.08; 1.23] | 0.48 [0.22; 1.06] | 0.48 [0.18; 1.28] | 0.51 [0.15; 1.73] | 0.48 [0.07; 3.12] | 0.53 [0.12; 2.41] | 0.59 [0.19; 1.79] | 0.61 [0.19; 2.00] | 0.67 [0.25; 1.78] | 0.68 [0.19; 2.38] | 0.76 [0.26; 2.19] | 0.76 [0.29; 2.02] | 0.77 [0.28; 2.15] | 0.79 [0.27; 2.35] | 0.84 [0.26; 2.71] | 0.86 [0.25; 2.93] | 0.91 [0.41; 2.01] | 1.02 [0.22; 4.77] | 0.99 [0.32; 3.09] | 0.98 [0.35; 2.73] | Clonidine | . | . | . | . | . | . |
| 0.22 [0.04; 1.19] | 0.30 [0.07; 1.31] | 0.30 [0.06; 1.51] | 0.45 [0.13; 1.53] | 0.45 [0.12; 1.70] | 0.47 [0.10; 2.17] | 0.45 [0.06; 3.57] | 0.49 [0.08; 2.92] | 0.55 [0.13; 2.30] | 0.57 [0.13; 2.52] | 0.63 [0.17; 2.37] | 0.63 [0.14; 2.97] | 0.71 [0.17; 2.86] | 0.71 [0.19; 2.68] | 0.72 [0.18; 2.82] | 0.74 [0.18; 3.03] | 0.78 [0.18; 3.43] | 0.80 [0.17; 3.69] | 0.85 [0.26; 2.81] | 0.95 [0.16; 5.76] | 0.92 [0.22; 3.93] | 0.91 [0.23; 3.57] | 0.93 [0.22; 3.92] | Thiamine | . | . | . | . | . |
| 0.20 [0.03; 1.14] | 0.26 [0.05; 1.28] | 0.26 [0.05; 1.45] | 0.40 [0.10; 1.52] | 0.39 [0.09; 1.66] | 0.42 [0.08; 2.10] | 0.39 [0.07; 2.28] | 0.43 [0.07; 2.79] | 0.48 [0.11; 2.08] | 0.50 [0.10; 2.44] | 0.55 [0.13; 2.32] | 0.56 [0.11; 2.86] | 0.62 [0.14; 2.79] | 0.63 [0.15; 2.63] | 0.63 [0.15; 2.75] | 0.65 [0.17; 2.49] | 0.69 [0.14; 3.32] | 0.70 [0.14; 3.56] | 0.74 [0.20; 2.78] | 0.83 [0.13; 5.49] | 0.81 [0.17; 3.82] | 0.80 [0.18; 3.49] | 0.82 [0.18; 3.81] | 0.88 [0.15; 5.21] | Haloperidol_low_dosage | . | . | . | . |
| ***0.20 [0.05; 0.90]** | ***0.27 [0.08; 0.96]** | 0.27 [0.06; 1.13] | 0.41 [0.16; 1.07] | 0.40 [0.14; 1.21] | 0.43 [0.11; 1.60] | 0.40 [0.06; 2.79] | 0.44 [0.09; 2.23] | 0.50 [0.15; 1.67] | 0.51 [0.14; 1.85] | 0.57 [0.19; 1.69] | 0.57 [0.15; 2.20] | 0.64 [0.20; 2.07] | 0.64 [0.22; 1.91] | 0.65 [0.21; 2.02] | 0.67 [0.20; 2.19] | 0.71 [0.20; 2.51] | 0.72 [0.19; 2.72] | 0.76 [0.30; 1.94] | 0.86 [0.17; 4.39] | 0.83 [0.24; 2.87] | 0.82 [0.26; 2.57] | 0.84 [0.25; 2.85] | 0.90 [0.20; 4.10] | 1.03 [0.20; 5.16] | Physostigmine | . | . | . |
| ***0.21 [0.05; 0.78]** | ***0.28 [0.09; 0.81]** | ***0.27 [0.08; 0.98]** | ***0.42 [0.21; 0.83]** | ***0.41 [0.17; 0.98]** | 0.44 [0.14; 1.36] | 0.41 [0.07; 2.53] | 0.45 [0.10; 1.97] | 0.51 [0.18; 1.40] | 0.52 [0.17; 1.57] | 0.58 [0.24; 1.37] | 0.58 [0.18; 1.88] | 0.65 [0.25; 1.72] | 0.66 [0.28; 1.55] | 0.66 [0.26; 1.66] | 0.68 [0.25; 1.83] | 0.72 [0.25; 2.12] | 0.73 [0.23; 2.32] | 0.78 [0.41; 1.49] | 0.88 [0.20; 3.89] | 0.85 [0.30; 2.41] | 0.84 [0.33; 2.11] | 0.86 [0.31; 2.39] | 0.92 [0.24; 3.59] | 1.05 [0.24; 4.56] | 1.02 [0.33; 3.18] | Ramelteon | . | . |
| 0.15 [0.02; 1.08] | 0.20 [0.03; 1.24] | 0.20 [0.03; 1.38] | 0.30 [0.06; 1.53] | 0.30 [0.06; 1.65] | 0.32 [0.05; 2.03] | 0.30 [0.03; 3.09] | 0.33 [0.04; 2.62] | 0.37 [0.06; 2.19] | 0.38 [0.06; 2.37] | 0.42 [0.08; 2.30] | 0.43 [0.07; 2.76] | 0.47 [0.08; 2.74] | 0.48 [0.09; 2.61] | 0.48 [0.09; 2.71] | 0.50 [0.08; 2.89] | 0.53 [0.09; 3.23] | 0.54 [0.08; 3.44] | 0.57 [0.11; 2.81] | 0.64 [0.08; 5.15] | 0.62 [0.10; 3.72] | 0.61 [0.11; 3.44] | 0.63 [0.11; 3.72] | 0.67 [0.09; 4.94] | 0.76 [0.10; 6.07] | 0.74 [0.12; 4.74] | 0.73 [0.13; 4.09] | Minocycline | . |
| ***0.16 [0.04; 0.66]** | ***0.22 [0.07; 0.70]** | ***0.22 [0.06; 0.83]** | ***0.33 [0.15; 0.70]** | ***0.33 [0.12; 0.86]** | 0.34 [0.10; 1.16] | 0.32 [0.05; 2.10] | 0.36 [0.10; 1.26] | 0.40 [0.13; 1.20] | 0.41 [0.13; 1.34] | 0.46 [0.17; 1.20] | 0.46 [0.13; 1.60] | 0.51 [0.22; 1.21] | 0.52 [0.20; 1.36] | 0.52 [0.19; 1.44] | 0.54 [0.18; 1.58] | 0.57 [0.18; 1.82] | 0.58 [0.17; 1.97] | 0.62 [0.28; 1.34] | 0.69 [0.23; 2.07] | 0.67 [0.22; 2.07] | 0.66 [0.24; 1.83] | 0.68 [0.23; 2.01] | 0.73 [0.17; 3.03] | 0.83 [0.18; 3.83] | 0.81 [0.24; 2.72] | 0.79 [0.29; 2.18] | 1.09 [0.18; 6.42] | Control |

Pairwise (upper-right portion) and network (lower-left portion) meta-analysis results are presented as estimate effect sizes for the outcome of incidence of delirium. Interventions are reported in order of mean ranking of incidence of delirium, and outcomes are expressed as risk ratio (RR) (95% confidence intervals). For the pairwise meta-analyses, RR of less than 1 indicate that the treatment specified in the row got less incidence of delirium than that specified in the column. For the network meta-analysis (NMA), RR of less than 1 indicate that the treatment specified in the column got less incidence of delirium than that specified in the row. Bold results marked with * indicate statistical significance.

**eTable 5D: League table of secondary outcome: all-cause mortality**

| Physostigmine | . | . | . | . | . | . | . | . | . | . | . | . | ***0.13 [0.02; 0.99]** | . | . | . | . |
| --- | --- | --- | --- | --- | --- | --- | --- | --- | --- | --- | --- | --- | --- | --- | --- | --- | --- |
| 0.19 [0.02; 1.51] | Dexmedetomidine | . | . | 0.85 [0.32; 2.24] | . | . | . | . | 1.00 [0.15; 6.64] | . | . | . | 0.68 [0.46; 1.02] | . | . | . | 0.25 [0.05; 1.14] |
| 0.38 [0.01; 16.84] | 2.04 [0.08; 50.54] | Methylprednisolone | . | . | . | . | . | . | . | . | . | . | 0.33 [0.01; 8.07] | . | . | . | . |
| 0.38 [0.01; 16.99] | 2.04 [0.08; 51.07] | 1.00 [0.01; 91.24] | Parecoxib | . | . | . | . | . | . | . | . | . | 0.33 [0.01; 8.15] | . | . | . | . |
| 0.17 [0.02; 1.61] | 0.92 [0.40; 2.14] | 0.45 [0.02; 12.33] | 0.45 [0.02; 12.45] | Control | . | . | . | 0.75 [0.20; 2.86] | . | . | . | . | . | . | . | . | . |
| 0.17 [0.01; 2.13] | 0.92 [0.21; 4.15] | 0.45 [0.01; 15.00] | 0.45 [0.01; 15.14] | 1.00 [0.18; 5.48] | Ramelteon | . | . | . | . | . | . | . | 0.74 [0.17; 3.15] | . | . | . | . |
| 0.15 [0.02; 1.19] | 0.79 [0.47; 1.35] | 0.39 [0.02; 9.59] | 0.39 [0.02; 9.69] | 0.86 [0.33; 2.23] | 0.86 [0.19; 3.84] | Ketamine_high_dosage | . | . | . | . | . | . | 0.86 [0.60; 1.24] | . | . | . | . |
| 0.16 [0.01; 2.04] | 0.87 [0.19; 4.02] | 0.42 [0.01; 14.29] | 0.42 [0.01; 14.42] | 0.94 [0.17; 5.29] | 0.94 [0.12; 7.50] | 1.09 [0.24; 5.05] | Neostigmine | . | . | . | . | . | 0.78 [0.18; 3.46] | . | . | . | . |
| 0.15 [0.02; 1.38] | 0.81 [0.34; 1.91] | 0.40 [0.01; 10.63] | 0.40 [0.01; 10.74] | 0.88 [0.33; 2.31] | 0.88 [0.17; 4.63] | 1.02 [0.42; 2.48] | 0.94 [0.17; 5.06] | Quetiapine | . | . | . | . | 0.78 [0.31; 1.95] | . | . | . | . |
| 0.13 [0.02; 1.04] | 0.71 [0.46; 1.09] | 0.35 [0.01; 8.42] | 0.35 [0.01; 8.51] | 0.77 [0.31; 1.89] | 0.77 [0.18; 3.31] | 0.89 [0.59; 1.35] | 0.81 [0.18; 3.64] | 0.87 [0.38; 2.00] | Haloperidol_high_dosage | . | . | 0.98 [0.76; 1.25] | 0.96 [0.79; 1.17] | . | . | . | . |
| 0.12 [0.00; 3.85] | 0.67 [0.04; 10.71] | 0.33 [0.00; 21.99] | 0.33 [0.00; 22.17] | 0.73 [0.04; 12.99] | 0.72 [0.03; 16.20] | 0.84 [0.05; 13.48] | 0.77 [0.03; 17.50] | 0.82 [0.05; 14.44] | 0.95 [0.06; 14.87] | Rivastigmine | . | . | 1.02 [0.07; 15.88] | . | . | . | . |
| 0.13 [0.02; 1.04] | 0.70 [0.45; 1.10] | 0.34 [0.01; 8.39] | 0.34 [0.01; 8.48] | 0.76 [0.31; 1.89] | 0.76 [0.18; 3.31] | 0.89 [0.58; 1.36] | 0.81 [0.18; 3.63] | 0.87 [0.37; 2.00] | 0.99 [0.74; 1.34] | 1.05 [0.07; 16.54] | Melatonin | . | 0.97 [0.77; 1.21] | . | . | . | . |
| 0.13 [0.02; 1.01] | 0.68 [0.43; 1.08] | 0.33 [0.01; 8.18] | 0.33 [0.01; 8.27] | 0.74 [0.30; 1.85] | 0.74 [0.17; 3.23] | 0.86 [0.56; 1.34] | 0.79 [0.18; 3.55] | 0.84 [0.36; 1.96] | 0.97 [0.76; 1.24] | 1.02 [0.06; 16.12] | 0.97 [0.70; 1.36] | Haloperidol_low_dosage | 1.00 [0.78; 1.28] | . | . | . | . |
| ***0.13 [0.02; 0.99]** | 0.68 [0.46; 1.00] | 0.33 [0.01; 8.07] | 0.33 [0.01; 8.15] | 0.74 [0.31; 1.78] | 0.74 [0.17; 3.15] | 0.86 [0.60; 1.24] | 0.78 [0.18; 3.46] | 0.84 [0.37; 1.88] | 0.96 [0.79; 1.17] | 1.02 [0.07; 15.88] | 0.97 [0.77; 1.21] | 1.00 [0.78; 1.27] | Placebo | 0.80 [0.22; 2.86] | 0.78 [0.39; 1.55] | 0.46 [0.10; 2.03] | . |
| 0.10 [0.01; 1.14] | 0.54 [0.14; 2.06] | 0.27 [0.01; 8.24] | 0.27 [0.01; 8.32] | 0.59 [0.13; 2.78] | 0.59 [0.09; 4.07] | 0.69 [0.18; 2.58] | 0.63 [0.09; 4.43] | 0.67 [0.15; 3.03] | 0.77 [0.21; 2.79] | 0.81 [0.04; 16.82] | 0.78 [0.21; 2.82] | 0.80 [0.22; 2.91] | 0.80 [0.22; 2.86] | Simvastatin | . | . | . |
| ***0.10 [0.01; 0.87]** | 0.53 [0.24; 1.17] | 0.26 [0.01; 6.77] | 0.26 [0.01; 6.84] | 0.58 [0.19; 1.76] | 0.58 [0.12; 2.87] | 0.67 [0.31; 1.46] | 0.61 [0.12; 3.14] | 0.65 [0.23; 1.89] | 0.75 [0.37; 1.53] | 0.79 [0.05; 13.48] | 0.76 [0.37; 1.56] | 0.78 [0.37; 1.61] | 0.78 [0.39; 1.55] | 0.98 [0.23; 4.14] | L_tryptophan | . | . |
| ***0.06 [0.00; 0.73]** | 0.31 [0.07; 1.45] | 0.15 [0.00; 5.14] | 0.15 [0.00; 5.18] | 0.34 [0.06; 1.91] | 0.34 [0.04; 2.70] | 0.39 [0.08; 1.82] | 0.36 [0.04; 2.94] | 0.38 [0.07; 2.09] | 0.44 [0.10; 1.98] | 0.46 [0.02; 10.59] | 0.44 [0.10; 2.00] | 0.45 [0.10; 2.06] | 0.46 [0.10; 2.03] | 0.57 [0.08; 4.06] | 0.59 [0.11; 3.02] | Dexamethasone | . |
| ***0.05 [0.00; 0.61]** | 0.25 [0.05; 1.14] | 0.12 [0.00; 4.23] | 0.12 [0.00; 4.27] | 0.27 [0.05; 1.54] | 0.27 [0.03; 2.29] | 0.31 [0.06; 1.58] | 0.28 [0.03; 2.48] | 0.30 [0.05; 1.76] | 0.35 [0.07; 1.71] | 0.37 [0.02; 8.78] | 0.35 [0.07; 1.73] | 0.36 [0.07; 1.78] | 0.36 [0.07; 1.76] | 0.45 [0.06; 3.45] | 0.46 [0.08; 2.60] | 0.79 [0.09; 6.97] | Clonidine |

Pairwise (upper-right portion) and network (lower-left portion) meta-analysis results are presented as estimate effect sizes for the outcome of all-cause mortality. Interventions are reported in order of mean ranking of all-cause mortality, and outcomes are expressed as risk ratio (RR) (95% confidence intervals). For the pairwise meta-analyses, RR of less than 1 indicate that the treatment specified in the row got less all-cause mortality than that specified in the column. For the network meta-analysis (NMA), RR of less than 1 indicate that the treatment specified in the column got less all-cause mortality than that specified in the row. Bold results marked with * indicate statistical significance.

**eTable 6: inconsistency within the network meta-analysis of primary outcome: incidence of delirium**

| Comparison | No.Studies | NMA | Direct | Indirect | Difference | Diff_95CI_lower | Diff_95CI_upper | p-value |
| --- | --- | --- | --- | --- | --- | --- | --- | --- |
| Aripiprazole:Clonidine | 0 | -1.18532 | NA | -1.18532 | NA | NA | NA | NA |
| Aripiprazole:Control | 0 | -1.51823 | NA | -1.51823 | NA | NA | NA | NA |
| Aripiprazole:Cyproheptadine | 0 | -0.1643 | NA | -0.1643 | NA | NA | NA | NA |
| Aripiprazole:Dexamethasone | 0 | -0.71496 | NA | -0.71496 | NA | NA | NA | NA |
| Aripiprazole:Dexmedetomidine | 0 | -0.46846 | NA | -0.46846 | NA | NA | NA | NA |
| Aripiprazole:Donepezil | 0 | -0.7377 | NA | -0.7377 | NA | NA | NA | NA |
| Aripiprazole:Flurbiprofen_sufentanil | 0 | -1.15051 | NA | -1.15051 | NA | NA | NA | NA |
| Aripiprazole:Gabapentin | 0 | -1.09898 | NA | -1.09898 | NA | NA | NA | NA |
| Aripiprazole:Haloperidol_high_dosage | 0 | -0.9572 | NA | -0.9572 | NA | NA | NA | NA |
| Aripiprazole:Haloperidol_Ketamine | 0 | -0.27769 | NA | -0.27769 | NA | NA | NA | NA |
| Aripiprazole:Haloperidol_low_dosage | 0 | -1.17955 | NA | -1.17955 | NA | NA | NA | NA |
| Aripiprazole:Haloperidol_medium_dosage | 0 | -0.84972 | NA | -0.84972 | NA | NA | NA | NA |
| Aripiprazole:Insulin | 0 | 0.3234 | NA | 0.3234 | NA | NA | NA | NA |
| Aripiprazole:Ketamine_high_dosage | 0 | -0.72358 | NA | -0.72358 | NA | NA | NA | NA |
| Aripiprazole:Ketamine_low_dosage | 0 | -0.54364 | NA | -0.54364 | NA | NA | NA | NA |
| Aripiprazole:L_tryptophan | 0 | -1.09521 | NA | -1.09521 | NA | NA | NA | NA |
| Aripiprazole:Lidocaine | 0 | -0.51986 | NA | -0.51986 | NA | NA | NA | NA |
| Aripiprazole:Melatonin | 0 | -0.87443 | NA | -0.87443 | NA | NA | NA | NA |
| Aripiprazole:Methylprednisolone | 0 | -0.39097 | NA | -0.39097 | NA | NA | NA | NA |
| Aripiprazole:Minocycline | 0 | -1.57649 | NA | -1.57649 | NA | NA | NA | NA |
| Aripiprazole:Neostigmine | 0 | -0.93288 | NA | -0.93288 | NA | NA | NA | NA |
| Aripiprazole:Olanzapine | 0 | 0.022908 | NA | 0.022908 | NA | NA | NA | NA |
| Aripiprazole:Ondansetron | 0 | -0.61018 | NA | -0.61018 | NA | NA | NA | NA |
| Aripiprazole:Parecoxib | 0 | -0.42968 | NA | -0.42968 | NA | NA | NA | NA |
| Aripiprazole:Physostigmine | 0 | -1.28163 | NA | -1.28163 | NA | NA | NA | NA |
| Aripiprazole:Placebo | 1 | -1.0116 | -1.0116 | NA | NA | NA | NA | NA |
| Aripiprazole:Quetiapine | 0 | -1.13487 | NA | -1.13487 | NA | NA | NA | NA |
| Aripiprazole:Ramelteon | 0 | -1.0408 | NA | -1.0408 | NA | NA | NA | NA |
| Aripiprazole:Risperidone | 0 | 0.038221 | NA | 0.038221 | NA | NA | NA | NA |
| Aripiprazole:Rivastigmine | 0 | -0.84569 | NA | -0.84569 | NA | NA | NA | NA |
| Aripiprazole:Simvastatin | 0 | -0.99656 | NA | -0.99656 | NA | NA | NA | NA |
| Aripiprazole:Suvorexant | 0 | 1.553348 | NA | 1.553348 | NA | NA | NA | NA |
| Aripiprazole:Thiamine | 0 | -1.1759 | NA | -1.1759 | NA | NA | NA | NA |
| Clonidine:Control | 0 | -0.33291 | NA | -0.33291 | NA | NA | NA | NA |
| Clonidine:Cyproheptadine | 0 | 1.021016 | NA | 1.021016 | NA | NA | NA | NA |
| Clonidine:Dexamethasone | 0 | 0.470354 | NA | 0.470354 | NA | NA | NA | NA |
| Clonidine:Dexmedetomidine | 1 | 0.716857 | 0.664574 | 0.760433 | -0.09586 | -1.36613 | 1.174413 | 0.882417 |
| Clonidine:Donepezil | 0 | 0.447621 | NA | 0.447621 | NA | NA | NA | NA |
| Clonidine:Flurbiprofen_sufentanil | 0 | 0.03481 | NA | 0.03481 | NA | NA | NA | NA |
| Clonidine:Gabapentin | 0 | 0.086338 | NA | 0.086338 | NA | NA | NA | NA |
| Clonidine:Haloperidol_high_dosage | 0 | 0.228115 | NA | 0.228115 | NA | NA | NA | NA |
| Clonidine:Haloperidol_Ketamine | 0 | 0.907625 | NA | 0.907625 | NA | NA | NA | NA |
| Clonidine:Haloperidol_low_dosage | 0 | 0.005767 | NA | 0.005767 | NA | NA | NA | NA |
| Clonidine:Haloperidol_medium_dosage | 0 | 0.335595 | NA | 0.335595 | NA | NA | NA | NA |
| Clonidine:Insulin | 0 | 1.50872 | NA | 1.50872 | NA | NA | NA | NA |
| Clonidine:Ketamine_high_dosage | 0 | 0.46174 | NA | 0.46174 | NA | NA | NA | NA |
| Clonidine:Ketamine_low_dosage | 0 | 0.64168 | NA | 0.64168 | NA | NA | NA | NA |
| Clonidine:L_tryptophan | 0 | 0.090112 | NA | 0.090112 | NA | NA | NA | NA |
| Clonidine:Lidocaine | 0 | 0.665457 | NA | 0.665457 | NA | NA | NA | NA |
| Clonidine:Melatonin | 0 | 0.310885 | NA | 0.310885 | NA | NA | NA | NA |
| Clonidine:Methylprednisolone | 0 | 0.794354 | NA | 0.794354 | NA | NA | NA | NA |
| Clonidine:Minocycline | 0 | -0.39117 | NA | -0.39117 | NA | NA | NA | NA |
| Clonidine:Neostigmine | 0 | 0.252437 | NA | 0.252437 | NA | NA | NA | NA |
| Clonidine:Olanzapine | 0 | 1.208228 | NA | 1.208228 | NA | NA | NA | NA |
| Clonidine:Ondansetron | 0 | 0.575135 | NA | 0.575135 | NA | NA | NA | NA |
| Clonidine:Parecoxib | 0 | 0.75564 | NA | 0.75564 | NA | NA | NA | NA |
| Clonidine:Physostigmine | 0 | -0.09631 | NA | -0.09631 | NA | NA | NA | NA |
| Clonidine:Placebo | 2 | 0.173719 | 0.214849 | 0.11899 | 0.095859 | -1.17441 | 1.366132 | 0.882417 |
| Clonidine:Quetiapine | 0 | 0.050449 | NA | 0.050449 | NA | NA | NA | NA |
| Clonidine:Ramelteon | 0 | 0.144524 | NA | 0.144524 | NA | NA | NA | NA |
| Clonidine:Risperidone | 0 | 1.223541 | NA | 1.223541 | NA | NA | NA | NA |
| Clonidine:Rivastigmine | 0 | 0.339631 | NA | 0.339631 | NA | NA | NA | NA |
| Clonidine:Simvastatin | 0 | 0.188756 | NA | 0.188756 | NA | NA | NA | NA |
| Clonidine:Suvorexant | 0 | 2.738668 | NA | 2.738668 | NA | NA | NA | NA |
| Clonidine:Thiamine | 0 | 0.009415 | NA | 0.009415 | NA | NA | NA | NA |
| Cyproheptadine:Control | 0 | -1.35393 | NA | -1.35393 | NA | NA | NA | NA |
| Dexamethasone:Control | 0 | -0.80327 | NA | -0.80327 | NA | NA | NA | NA |
| Dexmedetomidine:Control | 10 | -1.04977 | -0.98612 | -1.58453 | 0.59841 | -0.48499 | 1.681805 | 0.278995 |
| Donepezil:Control | 0 | -0.78054 | NA | -0.78054 | NA | NA | NA | NA |
| Flurbiprofen_sufentanil:Control | 1 | -0.36772 | -0.36772 | NA | NA | NA | NA | NA |
| Gabapentin:Control | 0 | -0.41925 | NA | -0.41925 | NA | NA | NA | NA |
| Haloperidol_high_dosage:Control | 0 | -0.56103 | NA | -0.56103 | NA | NA | NA | NA |
| Haloperidol_Ketamine:Control | 0 | -1.24054 | NA | -1.24054 | NA | NA | NA | NA |
| Haloperidol_low_dosage:Control | 0 | -0.33868 | NA | -0.33868 | NA | NA | NA | NA |
| Haloperidol_medium_dosage:Control | 0 | -0.66851 | NA | -0.66851 | NA | NA | NA | NA |
| Insulin:Control | 0 | -1.84163 | NA | -1.84163 | NA | NA | NA | NA |
| Ketamine_high_dosage:Control | 0 | -0.79466 | NA | -0.79466 | NA | NA | NA | NA |
| Ketamine_low_dosage:Control | 0 | -0.97459 | NA | -0.97459 | NA | NA | NA | NA |
| L_tryptophan:Control | 0 | -0.42303 | NA | -0.42303 | NA | NA | NA | NA |
| Lidocaine:Control | 1 | -0.99837 | -0.81093 | -2.44111 | 1.63018 | -2.07411 | 5.334465 | 0.38839 |
| Melatonin:Control | 0 | -0.6438 | NA | -0.6438 | NA | NA | NA | NA |
| Methylprednisolone:Control | 0 | -1.12727 | NA | -1.12727 | NA | NA | NA | NA |
| Minocycline:Control | 0 | 0.058259 | NA | 0.058259 | NA | NA | NA | NA |
| Neostigmine:Control | 0 | -0.58535 | NA | -0.58535 | NA | NA | NA | NA |
| Olanzapine:Control | 0 | -1.54114 | NA | -1.54114 | NA | NA | NA | NA |
| Ondansetron:Control | 0 | -0.90805 | NA | -0.90805 | NA | NA | NA | NA |
| Parecoxib:Control | 0 | -1.08856 | NA | -1.08856 | NA | NA | NA | NA |
| Physostigmine:Control | 0 | -0.23661 | NA | -0.23661 | NA | NA | NA | NA |
| Placebo:Control | 0 | -0.50663 | NA | -0.50663 | NA | NA | NA | NA |
| Quetiapine:Control | 1 | -0.38336 | -0.53422 | -0.00273 | -0.5315 | -2.07804 | 1.015048 | 0.500581 |
| Ramelteon:Control | 0 | -0.47744 | NA | -0.47744 | NA | NA | NA | NA |
| Risperidone:Control | 0 | -1.55646 | NA | -1.55646 | NA | NA | NA | NA |
| Rivastigmine:Control | 1 | -0.67255 | -1.02962 | -0.45846 | -0.57116 | -1.98037 | 0.838046 | 0.42697 |
| Simvastatin:Control | 0 | -0.52167 | NA | -0.52167 | NA | NA | NA | NA |
| Suvorexant:Control | 0 | -3.07158 | NA | -3.07158 | NA | NA | NA | NA |
| Thiamine:Control | 0 | -0.34233 | NA | -0.34233 | NA | NA | NA | NA |
| Cyproheptadine:Dexamethasone | 0 | -0.55066 | NA | -0.55066 | NA | NA | NA | NA |
| Cyproheptadine:Dexmedetomidine | 0 | -0.30416 | NA | -0.30416 | NA | NA | NA | NA |
| Cyproheptadine:Donepezil | 0 | -0.5734 | NA | -0.5734 | NA | NA | NA | NA |
| Cyproheptadine:Flurbiprofen_sufentanil | 0 | -0.98621 | NA | -0.98621 | NA | NA | NA | NA |
| Cyproheptadine:Gabapentin | 0 | -0.93468 | NA | -0.93468 | NA | NA | NA | NA |
| Cyproheptadine:Haloperidol_high_dosage | 0 | -0.7929 | NA | -0.7929 | NA | NA | NA | NA |
| Cyproheptadine:Haloperidol_Ketamine | 0 | -0.11339 | NA | -0.11339 | NA | NA | NA | NA |
| Cyproheptadine:Haloperidol_low_dosage | 0 | -1.01525 | NA | -1.01525 | NA | NA | NA | NA |
| Cyproheptadine:Haloperidol_medium_dosage | 0 | -0.68542 | NA | -0.68542 | NA | NA | NA | NA |
| Cyproheptadine:Insulin | 0 | 0.487703 | NA | 0.487703 | NA | NA | NA | NA |
| Cyproheptadine:Ketamine_high_dosage | 0 | -0.55928 | NA | -0.55928 | NA | NA | NA | NA |
| Cyproheptadine:Ketamine_low_dosage | 0 | -0.37934 | NA | -0.37934 | NA | NA | NA | NA |
| Cyproheptadine:L_tryptophan | 0 | -0.9309 | NA | -0.9309 | NA | NA | NA | NA |
| Cyproheptadine:Lidocaine | 0 | -0.35556 | NA | -0.35556 | NA | NA | NA | NA |
| Cyproheptadine:Melatonin | 0 | -0.71013 | NA | -0.71013 | NA | NA | NA | NA |
| Cyproheptadine:Methylprednisolone | 0 | -0.22666 | NA | -0.22666 | NA | NA | NA | NA |
| Cyproheptadine:Minocycline | 0 | -1.41219 | NA | -1.41219 | NA | NA | NA | NA |
| Cyproheptadine:Neostigmine | 0 | -0.76858 | NA | -0.76858 | NA | NA | NA | NA |
| Cyproheptadine:Olanzapine | 0 | 0.187212 | NA | 0.187212 | NA | NA | NA | NA |
| Cyproheptadine:Ondansetron | 0 | -0.44588 | NA | -0.44588 | NA | NA | NA | NA |
| Cyproheptadine:Parecoxib | 0 | -0.26538 | NA | -0.26538 | NA | NA | NA | NA |
| Cyproheptadine:Physostigmine | 0 | -1.11732 | NA | -1.11732 | NA | NA | NA | NA |
| Cyproheptadine:Placebo | 1 | -0.8473 | -0.8473 | NA | NA | NA | NA | NA |
| Cyproheptadine:Quetiapine | 0 | -0.97057 | NA | -0.97057 | NA | NA | NA | NA |
| Cyproheptadine:Ramelteon | 0 | -0.87649 | NA | -0.87649 | NA | NA | NA | NA |
| Cyproheptadine:Risperidone | 0 | 0.202524 | NA | 0.202524 | NA | NA | NA | NA |
| Cyproheptadine:Rivastigmine | 0 | -0.68139 | NA | -0.68139 | NA | NA | NA | NA |
| Cyproheptadine:Simvastatin | 0 | -0.83226 | NA | -0.83226 | NA | NA | NA | NA |
| Cyproheptadine:Suvorexant | 0 | 1.717651 | NA | 1.717651 | NA | NA | NA | NA |
| Cyproheptadine:Thiamine | 0 | -1.0116 | NA | -1.0116 | NA | NA | NA | NA |
| Dexamethasone:Dexmedetomidine | 0 | 0.246502 | NA | 0.246502 | NA | NA | NA | NA |
| Dexamethasone:Donepezil | 0 | -0.02273 | NA | -0.02273 | NA | NA | NA | NA |
| Dexamethasone:Flurbiprofen_sufentanil | 0 | -0.43554 | NA | -0.43554 | NA | NA | NA | NA |
| Dexamethasone:Gabapentin | 0 | -0.38402 | NA | -0.38402 | NA | NA | NA | NA |
| Dexamethasone:Haloperidol_high_dosage | 0 | -0.24224 | NA | -0.24224 | NA | NA | NA | NA |
| Dexamethasone:Haloperidol_Ketamine | 0 | 0.437271 | NA | 0.437271 | NA | NA | NA | NA |
| Dexamethasone:Haloperidol_low_dosage | 0 | -0.46459 | NA | -0.46459 | NA | NA | NA | NA |
| Dexamethasone:Haloperidol_medium_dosage | 0 | -0.13476 | NA | -0.13476 | NA | NA | NA | NA |
| Dexamethasone:Insulin | 0 | 1.038365 | NA | 1.038365 | NA | NA | NA | NA |
| Dexamethasone:Ketamine_high_dosage | 0 | -0.00861 | NA | -0.00861 | NA | NA | NA | NA |
| Dexamethasone:Ketamine_low_dosage | 0 | 0.171325 | NA | 0.171325 | NA | NA | NA | NA |
| Dexamethasone:L_tryptophan | 0 | -0.38024 | NA | -0.38024 | NA | NA | NA | NA |
| Dexamethasone:Lidocaine | 0 | 0.195103 | NA | 0.195103 | NA | NA | NA | NA |
| Dexamethasone:Melatonin | 0 | -0.15947 | NA | -0.15947 | NA | NA | NA | NA |
| Dexamethasone:Methylprednisolone | 1 | 0.323999 | 2.360854 | 0.188013 | 2.172841 | -0.84606 | 5.191739 | 0.15834 |
| Dexamethasone:Minocycline | 0 | -0.86153 | NA | -0.86153 | NA | NA | NA | NA |
| Dexamethasone:Neostigmine | 0 | -0.21792 | NA | -0.21792 | NA | NA | NA | NA |
| Dexamethasone:Olanzapine | 0 | 0.737873 | NA | 0.737873 | NA | NA | NA | NA |
| Dexamethasone:Ondansetron | 0 | 0.104781 | NA | 0.104781 | NA | NA | NA | NA |
| Dexamethasone:Parecoxib | 0 | 0.285286 | NA | 0.285286 | NA | NA | NA | NA |
| Dexamethasone:Physostigmine | 0 | -0.56666 | NA | -0.56666 | NA | NA | NA | NA |
| Dexamethasone:Placebo | 3 | -0.29664 | -0.3637 | 1.809143 | -2.17284 | -5.19174 | 0.846058 | 0.15834 |
| Dexamethasone:Quetiapine | 0 | -0.41991 | NA | -0.41991 | NA | NA | NA | NA |
| Dexamethasone:Ramelteon | 0 | -0.32583 | NA | -0.32583 | NA | NA | NA | NA |
| Dexamethasone:Risperidone | 0 | 0.753186 | NA | 0.753186 | NA | NA | NA | NA |
| Dexamethasone:Rivastigmine | 0 | -0.13072 | NA | -0.13072 | NA | NA | NA | NA |
| Dexamethasone:Simvastatin | 0 | -0.2816 | NA | -0.2816 | NA | NA | NA | NA |
| Dexamethasone:Suvorexant | 0 | 2.268313 | NA | 2.268313 | NA | NA | NA | NA |
| Dexamethasone:Thiamine | 0 | -0.46094 | NA | -0.46094 | NA | NA | NA | NA |
| Dexmedetomidine:Donepezil | 0 | -0.26924 | NA | -0.26924 | NA | NA | NA | NA |
| Dexmedetomidine:Flurbiprofen_sufentanil | 0 | -0.68205 | NA | -0.68205 | NA | NA | NA | NA |
| Dexmedetomidine:Gabapentin | 0 | -0.63052 | NA | -0.63052 | NA | NA | NA | NA |
| Dexmedetomidine:Haloperidol_high_dosage | 1 | -0.48874 | -1.20397 | -0.4072 | -0.79677 | -2.23437 | 0.64083 | 0.277354 |
| Dexmedetomidine:Haloperidol_Ketamine | 0 | 0.190769 | NA | 0.190769 | NA | NA | NA | NA |
| Dexmedetomidine:Haloperidol_low_dosage | 0 | -0.71109 | NA | -0.71109 | NA | NA | NA | NA |
| Dexmedetomidine:Haloperidol_medium_dosage | 0 | -0.38126 | NA | -0.38126 | NA | NA | NA | NA |
| Dexmedetomidine:Insulin | 0 | 0.791863 | NA | 0.791863 | NA | NA | NA | NA |
| Dexmedetomidine:Ketamine_high_dosage | 0 | -0.25512 | NA | -0.25512 | NA | NA | NA | NA |
| Dexmedetomidine:Ketamine_low_dosage | 0 | -0.07518 | NA | -0.07518 | NA | NA | NA | NA |
| Dexmedetomidine:L_tryptophan | 0 | -0.62674 | NA | -0.62674 | NA | NA | NA | NA |
| Dexmedetomidine:Lidocaine | 1 | -0.0514 | 0.257045 | -0.94524 | 1.202289 | -1.52969 | 3.934272 | 0.38839 |
| Dexmedetomidine:Melatonin | 0 | -0.40597 | NA | -0.40597 | NA | NA | NA | NA |
| Dexmedetomidine:Methylprednisolone | 0 | 0.077497 | NA | 0.077497 | NA | NA | NA | NA |
| Dexmedetomidine:Minocycline | 0 | -1.10803 | NA | -1.10803 | NA | NA | NA | NA |
| Dexmedetomidine:Neostigmine | 0 | -0.46442 | NA | -0.46442 | NA | NA | NA | NA |
| Dexmedetomidine:Olanzapine | 0 | 0.491371 | NA | 0.491371 | NA | NA | NA | NA |
| Dexmedetomidine:Ondansetron | 0 | -0.14172 | NA | -0.14172 | NA | NA | NA | NA |
| Dexmedetomidine:Parecoxib | 0 | 0.038784 | NA | 0.038784 | NA | NA | NA | NA |
| Dexmedetomidine:Physostigmine | 0 | -0.81317 | NA | -0.81317 | NA | NA | NA | NA |
| Dexmedetomidine:Placebo | 21 | -0.54314 | -0.56756 | -0.17977 | -0.38779 | -1.21239 | 0.43682 | 0.35668 |
| Dexmedetomidine:Quetiapine | 0 | -0.66641 | NA | -0.66641 | NA | NA | NA | NA |
| Dexmedetomidine:Ramelteon | 0 | -0.57233 | NA | -0.57233 | NA | NA | NA | NA |
| Dexmedetomidine:Risperidone | 0 | 0.506684 | NA | 0.506684 | NA | NA | NA | NA |
| Dexmedetomidine:Rivastigmine | 0 | -0.37723 | NA | -0.37723 | NA | NA | NA | NA |
| Dexmedetomidine:Simvastatin | 0 | -0.5281 | NA | -0.5281 | NA | NA | NA | NA |
| Dexmedetomidine:Suvorexant | 0 | 2.021811 | NA | 2.021811 | NA | NA | NA | NA |
| Dexmedetomidine:Thiamine | 0 | -0.70744 | NA | -0.70744 | NA | NA | NA | NA |
| Donepezil:Flurbiprofen_sufentanil | 0 | -0.41281 | NA | -0.41281 | NA | NA | NA | NA |
| Donepezil:Gabapentin | 0 | -0.36128 | NA | -0.36128 | NA | NA | NA | NA |
| Donepezil:Haloperidol_high_dosage | 0 | -0.21951 | NA | -0.21951 | NA | NA | NA | NA |
| Donepezil:Haloperidol_Ketamine | 0 | 0.460004 | NA | 0.460004 | NA | NA | NA | NA |
| Donepezil:Haloperidol_low_dosage | 0 | -0.44185 | NA | -0.44185 | NA | NA | NA | NA |
| Donepezil:Haloperidol_medium_dosage | 0 | -0.11203 | NA | -0.11203 | NA | NA | NA | NA |
| Donepezil:Insulin | 0 | 1.061098 | NA | 1.061098 | NA | NA | NA | NA |
| Donepezil:Ketamine_high_dosage | 0 | 0.014119 | NA | 0.014119 | NA | NA | NA | NA |
| Donepezil:Ketamine_low_dosage | 0 | 0.194059 | NA | 0.194059 | NA | NA | NA | NA |
| Donepezil:L_tryptophan | 0 | -0.35751 | NA | -0.35751 | NA | NA | NA | NA |
| Donepezil:Lidocaine | 0 | 0.217836 | NA | 0.217836 | NA | NA | NA | NA |
| Donepezil:Melatonin | 0 | -0.13674 | NA | -0.13674 | NA | NA | NA | NA |
| Donepezil:Methylprednisolone | 0 | 0.346732 | NA | 0.346732 | NA | NA | NA | NA |
| Donepezil:Minocycline | 0 | -0.8388 | NA | -0.8388 | NA | NA | NA | NA |
| Donepezil:Neostigmine | 0 | -0.19518 | NA | -0.19518 | NA | NA | NA | NA |
| Donepezil:Olanzapine | 0 | 0.760607 | NA | 0.760607 | NA | NA | NA | NA |
| Donepezil:Ondansetron | 0 | 0.127514 | NA | 0.127514 | NA | NA | NA | NA |
| Donepezil:Parecoxib | 0 | 0.308019 | NA | 0.308019 | NA | NA | NA | NA |
| Donepezil:Physostigmine | 0 | -0.54393 | NA | -0.54393 | NA | NA | NA | NA |
| Donepezil:Placebo | 2 | -0.2739 | -0.2739 | NA | NA | NA | NA | NA |
| Donepezil:Quetiapine | 0 | -0.39717 | NA | -0.39717 | NA | NA | NA | NA |
| Donepezil:Ramelteon | 0 | -0.3031 | NA | -0.3031 | NA | NA | NA | NA |
| Donepezil:Risperidone | 0 | 0.77592 | NA | 0.77592 | NA | NA | NA | NA |
| Donepezil:Rivastigmine | 0 | -0.10799 | NA | -0.10799 | NA | NA | NA | NA |
| Donepezil:Simvastatin | 0 | -0.25886 | NA | -0.25886 | NA | NA | NA | NA |
| Donepezil:Suvorexant | 0 | 2.291047 | NA | 2.291047 | NA | NA | NA | NA |
| Donepezil:Thiamine | 0 | -0.43821 | NA | -0.43821 | NA | NA | NA | NA |
| Flurbiprofen_sufentanil:Gabapentin | 0 | 0.051528 | NA | 0.051528 | NA | NA | NA | NA |
| Flurbiprofen_sufentanil:Haloperidol_high_dosage | 0 | 0.193305 | NA | 0.193305 | NA | NA | NA | NA |
| Flurbiprofen_sufentanil:Haloperidol_Ketamine | 0 | 0.872815 | NA | 0.872815 | NA | NA | NA | NA |
| Flurbiprofen_sufentanil:Haloperidol_low_dosage | 0 | -0.02904 | NA | -0.02904 | NA | NA | NA | NA |
| Flurbiprofen_sufentanil:Haloperidol_medium_dosage | 0 | 0.300785 | NA | 0.300785 | NA | NA | NA | NA |
| Flurbiprofen_sufentanil:Insulin | 0 | 1.47391 | NA | 1.47391 | NA | NA | NA | NA |
| Flurbiprofen_sufentanil:Ketamine_high_dosage | 0 | 0.426931 | NA | 0.426931 | NA | NA | NA | NA |
| Flurbiprofen_sufentanil:Ketamine_low_dosage | 0 | 0.60687 | NA | 0.60687 | NA | NA | NA | NA |
| Flurbiprofen_sufentanil:L_tryptophan | 0 | 0.055302 | NA | 0.055302 | NA | NA | NA | NA |
| Flurbiprofen_sufentanil:Lidocaine | 0 | 0.630647 | NA | 0.630647 | NA | NA | NA | NA |
| Flurbiprofen_sufentanil:Melatonin | 0 | 0.276075 | NA | 0.276075 | NA | NA | NA | NA |
| Flurbiprofen_sufentanil:Methylprednisolone | 0 | 0.759544 | NA | 0.759544 | NA | NA | NA | NA |
| Flurbiprofen_sufentanil:Minocycline | 0 | -0.42598 | NA | -0.42598 | NA | NA | NA | NA |
| Flurbiprofen_sufentanil:Neostigmine | 0 | 0.217627 | NA | 0.217627 | NA | NA | NA | NA |
| Flurbiprofen_sufentanil:Olanzapine | 0 | 1.173418 | NA | 1.173418 | NA | NA | NA | NA |
| Flurbiprofen_sufentanil:Ondansetron | 0 | 0.540325 | NA | 0.540325 | NA | NA | NA | NA |
| Flurbiprofen_sufentanil:Parecoxib | 0 | 0.72083 | NA | 0.72083 | NA | NA | NA | NA |
| Flurbiprofen_sufentanil:Physostigmine | 0 | -0.13112 | NA | -0.13112 | NA | NA | NA | NA |
| Flurbiprofen_sufentanil:Placebo | 0 | 0.138909 | NA | 0.138909 | NA | NA | NA | NA |
| Flurbiprofen_sufentanil:Quetiapine | 0 | 0.015639 | NA | 0.015639 | NA | NA | NA | NA |
| Flurbiprofen_sufentanil:Ramelteon | 0 | 0.109714 | NA | 0.109714 | NA | NA | NA | NA |
| Flurbiprofen_sufentanil:Risperidone | 0 | 1.188731 | NA | 1.188731 | NA | NA | NA | NA |
| Flurbiprofen_sufentanil:Rivastigmine | 0 | 0.304821 | NA | 0.304821 | NA | NA | NA | NA |
| Flurbiprofen_sufentanil:Simvastatin | 0 | 0.153947 | NA | 0.153947 | NA | NA | NA | NA |
| Flurbiprofen_sufentanil:Suvorexant | 0 | 2.703858 | NA | 2.703858 | NA | NA | NA | NA |
| Flurbiprofen_sufentanil:Thiamine | 0 | -0.02539 | NA | -0.02539 | NA | NA | NA | NA |
| Gabapentin:Haloperidol_high_dosage | 0 | 0.141777 | NA | 0.141777 | NA | NA | NA | NA |
| Gabapentin:Haloperidol_Ketamine | 0 | 0.821288 | NA | 0.821288 | NA | NA | NA | NA |
| Gabapentin:Haloperidol_low_dosage | 0 | -0.08057 | NA | -0.08057 | NA | NA | NA | NA |
| Gabapentin:Haloperidol_medium_dosage | 0 | 0.249257 | NA | 0.249257 | NA | NA | NA | NA |
| Gabapentin:Insulin | 0 | 1.422382 | NA | 1.422382 | NA | NA | NA | NA |
| Gabapentin:Ketamine_high_dosage | 0 | 0.375403 | NA | 0.375403 | NA | NA | NA | NA |
| Gabapentin:Ketamine_low_dosage | 0 | 0.555342 | NA | 0.555342 | NA | NA | NA | NA |
| Gabapentin:L_tryptophan | 0 | 0.003775 | NA | 0.003775 | NA | NA | NA | NA |
| Gabapentin:Lidocaine | 0 | 0.579119 | NA | 0.579119 | NA | NA | NA | NA |
| Gabapentin:Melatonin | 0 | 0.224547 | NA | 0.224547 | NA | NA | NA | NA |
| Gabapentin:Methylprednisolone | 0 | 0.708016 | NA | 0.708016 | NA | NA | NA | NA |
| Gabapentin:Minocycline | 0 | -0.47751 | NA | -0.47751 | NA | NA | NA | NA |
| Gabapentin:Neostigmine | 0 | 0.1661 | NA | 0.1661 | NA | NA | NA | NA |
| Gabapentin:Olanzapine | 0 | 1.12189 | NA | 1.12189 | NA | NA | NA | NA |
| Gabapentin:Ondansetron | 0 | 0.488798 | NA | 0.488798 | NA | NA | NA | NA |
| Gabapentin:Parecoxib | 0 | 0.669303 | NA | 0.669303 | NA | NA | NA | NA |
| Gabapentin:Physostigmine | 0 | -0.18265 | NA | -0.18265 | NA | NA | NA | NA |
| Gabapentin:Placebo | 3 | 0.087381 | 0.087381 | NA | NA | NA | NA | NA |
| Gabapentin:Quetiapine | 0 | -0.03589 | NA | -0.03589 | NA | NA | NA | NA |
| Gabapentin:Ramelteon | 0 | 0.058187 | NA | 0.058187 | NA | NA | NA | NA |
| Gabapentin:Risperidone | 0 | 1.137203 | NA | 1.137203 | NA | NA | NA | NA |
| Gabapentin:Rivastigmine | 0 | 0.253294 | NA | 0.253294 | NA | NA | NA | NA |
| Gabapentin:Simvastatin | 0 | 0.102419 | NA | 0.102419 | NA | NA | NA | NA |
| Gabapentin:Suvorexant | 0 | 2.65233 | NA | 2.65233 | NA | NA | NA | NA |
| Gabapentin:Thiamine | 0 | -0.07692 | NA | -0.07692 | NA | NA | NA | NA |
| Haloperidol_high_dosage:Haloperidol_Ketamine | 0 | 0.679511 | NA | 0.679511 | NA | NA | NA | NA |
| Haloperidol_high_dosage:Haloperidol_low_dosage | 1 | -0.22235 | -0.17515 | -0.3485 | 0.173344 | -1.14587 | 1.492558 | 0.796763 |
| Haloperidol_high_dosage:Haloperidol_medium_dosage | 0 | 0.10748 | NA | 0.10748 | NA | NA | NA | NA |
| Haloperidol_high_dosage:Insulin | 0 | 1.280605 | NA | 1.280605 | NA | NA | NA | NA |
| Haloperidol_high_dosage:Ketamine_high_dosage | 0 | 0.233626 | NA | 0.233626 | NA | NA | NA | NA |
| Haloperidol_high_dosage:Ketamine_low_dosage | 0 | 0.413565 | NA | 0.413565 | NA | NA | NA | NA |
| Haloperidol_high_dosage:L_tryptophan | 0 | -0.138 | NA | -0.138 | NA | NA | NA | NA |
| Haloperidol_high_dosage:Lidocaine | 0 | 0.437342 | NA | 0.437342 | NA | NA | NA | NA |
| Haloperidol_high_dosage:Melatonin | 0 | 0.08277 | NA | 0.08277 | NA | NA | NA | NA |
| Haloperidol_high_dosage:Methylprednisolone | 0 | 0.566239 | NA | 0.566239 | NA | NA | NA | NA |
| Haloperidol_high_dosage:Minocycline | 0 | -0.61929 | NA | -0.61929 | NA | NA | NA | NA |
| Haloperidol_high_dosage:Neostigmine | 0 | 0.024323 | NA | 0.024323 | NA | NA | NA | NA |
| Haloperidol_high_dosage:Olanzapine | 0 | 0.980113 | NA | 0.980113 | NA | NA | NA | NA |
| Haloperidol_high_dosage:Ondansetron | 0 | 0.34702 | NA | 0.34702 | NA | NA | NA | NA |
| Haloperidol_high_dosage:Parecoxib | 0 | 0.527525 | NA | 0.527525 | NA | NA | NA | NA |
| Haloperidol_high_dosage:Physostigmine | 0 | -0.32442 | NA | -0.32442 | NA | NA | NA | NA |
| Haloperidol_high_dosage:Placebo | 4 | -0.0544 | -0.09493 | 0.950655 | -1.04558 | -3.07321 | 0.982049 | 0.312165 |
| Haloperidol_high_dosage:Quetiapine | 0 | -0.17767 | NA | -0.17767 | NA | NA | NA | NA |
| Haloperidol_high_dosage:Ramelteon | 0 | -0.08359 | NA | -0.08359 | NA | NA | NA | NA |
| Haloperidol_high_dosage:Risperidone | 0 | 0.995426 | NA | 0.995426 | NA | NA | NA | NA |
| Haloperidol_high_dosage:Rivastigmine | 0 | 0.111517 | NA | 0.111517 | NA | NA | NA | NA |
| Haloperidol_high_dosage:Simvastatin | 0 | -0.03936 | NA | -0.03936 | NA | NA | NA | NA |
| Haloperidol_high_dosage:Suvorexant | 0 | 2.510553 | NA | 2.510553 | NA | NA | NA | NA |
| Haloperidol_high_dosage:Thiamine | 0 | -0.2187 | NA | -0.2187 | NA | NA | NA | NA |
| Haloperidol_Ketamine:Haloperidol_low_dosage | 1 | -0.90186 | -0.93827 | -0.67857 | -0.2597 | -4.85967 | 4.340277 | 0.911893 |
| Haloperidol_Ketamine:Haloperidol_medium_dosage | 0 | -0.57203 | NA | -0.57203 | NA | NA | NA | NA |
| Haloperidol_Ketamine:Insulin | 0 | 0.601094 | NA | 0.601094 | NA | NA | NA | NA |
| Haloperidol_Ketamine:Ketamine_high_dosage | 1 | -0.44588 | -0.38396 | -0.63253 | 0.248573 | -3.49033 | 3.987478 | 0.896326 |
| Haloperidol_Ketamine:Ketamine_low_dosage | 0 | -0.26595 | NA | -0.26595 | NA | NA | NA | NA |
| Haloperidol_Ketamine:L_tryptophan | 0 | -0.81751 | NA | -0.81751 | NA | NA | NA | NA |
| Haloperidol_Ketamine:Lidocaine | 0 | -0.24217 | NA | -0.24217 | NA | NA | NA | NA |
| Haloperidol_Ketamine:Melatonin | 0 | -0.59674 | NA | -0.59674 | NA | NA | NA | NA |
| Haloperidol_Ketamine:Methylprednisolone | 0 | -0.11327 | NA | -0.11327 | NA | NA | NA | NA |
| Haloperidol_Ketamine:Minocycline | 0 | -1.2988 | NA | -1.2988 | NA | NA | NA | NA |
| Haloperidol_Ketamine:Neostigmine | 0 | -0.65519 | NA | -0.65519 | NA | NA | NA | NA |
| Haloperidol_Ketamine:Olanzapine | 0 | 0.300603 | NA | 0.300603 | NA | NA | NA | NA |
| Haloperidol_Ketamine:Ondansetron | 0 | -0.33249 | NA | -0.33249 | NA | NA | NA | NA |
| Haloperidol_Ketamine:Parecoxib | 0 | -0.15199 | NA | -0.15199 | NA | NA | NA | NA |
| Haloperidol_Ketamine:Physostigmine | 0 | -1.00393 | NA | -1.00393 | NA | NA | NA | NA |
| Haloperidol_Ketamine:Placebo | 1 | -0.73391 | -0.7376 | -0.71975 | -0.01785 | -3.92415 | 3.888455 | 0.992855 |
| Haloperidol_Ketamine:Quetiapine | 0 | -0.85718 | NA | -0.85718 | NA | NA | NA | NA |
| Haloperidol_Ketamine:Ramelteon | 0 | -0.7631 | NA | -0.7631 | NA | NA | NA | NA |
| Haloperidol_Ketamine:Risperidone | 0 | 0.315915 | NA | 0.315915 | NA | NA | NA | NA |
| Haloperidol_Ketamine:Rivastigmine | 0 | -0.56799 | NA | -0.56799 | NA | NA | NA | NA |
| Haloperidol_Ketamine:Simvastatin | 0 | -0.71887 | NA | -0.71887 | NA | NA | NA | NA |
| Haloperidol_Ketamine:Suvorexant | 0 | 1.831043 | NA | 1.831043 | NA | NA | NA | NA |
| Haloperidol_Ketamine:Thiamine | 0 | -0.89821 | NA | -0.89821 | NA | NA | NA | NA |
| Haloperidol_low_dosage:Haloperidol_medium_dosage | 0 | 0.329827 | NA | 0.329827 | NA | NA | NA | NA |
| Haloperidol_low_dosage:Insulin | 0 | 1.502952 | NA | 1.502952 | NA | NA | NA | NA |
| Haloperidol_low_dosage:Ketamine_high_dosage | 1 | 0.455973 | 0.554311 | 0.426326 | 0.127984 | -1.61278 | 1.868753 | 0.885422 |
| Haloperidol_low_dosage:Ketamine_low_dosage | 0 | 0.635912 | NA | 0.635912 | NA | NA | NA | NA |
| Haloperidol_low_dosage:L_tryptophan | 0 | 0.084345 | NA | 0.084345 | NA | NA | NA | NA |
| Haloperidol_low_dosage:Lidocaine | 0 | 0.65969 | NA | 0.65969 | NA | NA | NA | NA |
| Haloperidol_low_dosage:Melatonin | 0 | 0.305117 | NA | 0.305117 | NA | NA | NA | NA |
| Haloperidol_low_dosage:Methylprednisolone | 0 | 0.788586 | NA | 0.788586 | NA | NA | NA | NA |
| Haloperidol_low_dosage:Minocycline | 0 | -0.39694 | NA | -0.39694 | NA | NA | NA | NA |
| Haloperidol_low_dosage:Neostigmine | 0 | 0.24667 | NA | 0.24667 | NA | NA | NA | NA |
| Haloperidol_low_dosage:Olanzapine | 0 | 1.202461 | NA | 1.202461 | NA | NA | NA | NA |
| Haloperidol_low_dosage:Ondansetron | 0 | 0.569368 | NA | 0.569368 | NA | NA | NA | NA |
| Haloperidol_low_dosage:Parecoxib | 0 | 0.749873 | NA | 0.749873 | NA | NA | NA | NA |
| Haloperidol_low_dosage:Physostigmine | 0 | -0.10208 | NA | -0.10208 | NA | NA | NA | NA |
| Haloperidol_low_dosage:Placebo | 2 | 0.167951 | 0.189241 | 0.067056 | 0.122184 | -1.36144 | 1.605809 | 0.871768 |
| Haloperidol_low_dosage:Quetiapine | 0 | 0.044681 | NA | 0.044681 | NA | NA | NA | NA |
| Haloperidol_low_dosage:Ramelteon | 0 | 0.138757 | NA | 0.138757 | NA | NA | NA | NA |
| Haloperidol_low_dosage:Risperidone | 0 | 1.217773 | NA | 1.217773 | NA | NA | NA | NA |
| Haloperidol_low_dosage:Rivastigmine | 0 | 0.333864 | NA | 0.333864 | NA | NA | NA | NA |
| Haloperidol_low_dosage:Simvastatin | 0 | 0.182989 | NA | 0.182989 | NA | NA | NA | NA |
| Haloperidol_low_dosage:Suvorexant | 0 | 2.732901 | NA | 2.732901 | NA | NA | NA | NA |
| Haloperidol_low_dosage:Thiamine | 0 | 0.003648 | NA | 0.003648 | NA | NA | NA | NA |
| Haloperidol_medium_dosage:Insulin | 0 | 1.173125 | NA | 1.173125 | NA | NA | NA | NA |
| Haloperidol_medium_dosage:Ketamine_high_dosage | 0 | 0.126146 | NA | 0.126146 | NA | NA | NA | NA |
| Haloperidol_medium_dosage:Ketamine_low_dosage | 0 | 0.306085 | NA | 0.306085 | NA | NA | NA | NA |
| Haloperidol_medium_dosage:L_tryptophan | 0 | -0.24548 | NA | -0.24548 | NA | NA | NA | NA |
| Haloperidol_medium_dosage:Lidocaine | 0 | 0.329862 | NA | 0.329862 | NA | NA | NA | NA |
| Haloperidol_medium_dosage:Melatonin | 0 | -0.02471 | NA | -0.02471 | NA | NA | NA | NA |
| Haloperidol_medium_dosage:Methylprednisolone | 0 | 0.458759 | NA | 0.458759 | NA | NA | NA | NA |
| Haloperidol_medium_dosage:Minocycline | 0 | -0.72677 | NA | -0.72677 | NA | NA | NA | NA |
| Haloperidol_medium_dosage:Neostigmine | 0 | -0.08316 | NA | -0.08316 | NA | NA | NA | NA |
| Haloperidol_medium_dosage:Olanzapine | 0 | 0.872633 | NA | 0.872633 | NA | NA | NA | NA |
| Haloperidol_medium_dosage:Ondansetron | 0 | 0.23954 | NA | 0.23954 | NA | NA | NA | NA |
| Haloperidol_medium_dosage:Parecoxib | 0 | 0.420045 | NA | 0.420045 | NA | NA | NA | NA |
| Haloperidol_medium_dosage:Physostigmine | 0 | -0.4319 | NA | -0.4319 | NA | NA | NA | NA |
| Haloperidol_medium_dosage:Placebo | 2 | -0.16188 | -0.16188 | NA | NA | NA | NA | NA |
| Haloperidol_medium_dosage:Quetiapine | 0 | -0.28515 | NA | -0.28515 | NA | NA | NA | NA |
| Haloperidol_medium_dosage:Ramelteon | 0 | -0.19107 | NA | -0.19107 | NA | NA | NA | NA |
| Haloperidol_medium_dosage:Risperidone | 0 | 0.887946 | NA | 0.887946 | NA | NA | NA | NA |
| Haloperidol_medium_dosage:Rivastigmine | 0 | 0.004037 | NA | 0.004037 | NA | NA | NA | NA |
| Haloperidol_medium_dosage:Simvastatin | 0 | -0.14684 | NA | -0.14684 | NA | NA | NA | NA |
| Haloperidol_medium_dosage:Suvorexant | 0 | 2.403073 | NA | 2.403073 | NA | NA | NA | NA |
| Haloperidol_medium_dosage:Thiamine | 0 | -0.32618 | NA | -0.32618 | NA | NA | NA | NA |
| Insulin:Ketamine_high_dosage | 0 | -1.04698 | NA | -1.04698 | NA | NA | NA | NA |
| Insulin:Ketamine_low_dosage | 0 | -0.86704 | NA | -0.86704 | NA | NA | NA | NA |
| Insulin:L_tryptophan | 0 | -1.41861 | NA | -1.41861 | NA | NA | NA | NA |
| Insulin:Lidocaine | 0 | -0.84326 | NA | -0.84326 | NA | NA | NA | NA |
| Insulin:Melatonin | 0 | -1.19783 | NA | -1.19783 | NA | NA | NA | NA |
| Insulin:Methylprednisolone | 0 | -0.71437 | NA | -0.71437 | NA | NA | NA | NA |
| Insulin:Minocycline | 0 | -1.89989 | NA | -1.89989 | NA | NA | NA | NA |
| Insulin:Neostigmine | 0 | -1.25628 | NA | -1.25628 | NA | NA | NA | NA |
| Insulin:Olanzapine | 0 | -0.30049 | NA | -0.30049 | NA | NA | NA | NA |
| Insulin:Ondansetron | 0 | -0.93358 | NA | -0.93358 | NA | NA | NA | NA |
| Insulin:Parecoxib | 0 | -0.75308 | NA | -0.75308 | NA | NA | NA | NA |
| Insulin:Physostigmine | 0 | -1.60503 | NA | -1.60503 | NA | NA | NA | NA |
| Insulin:Placebo | 1 | -1.335 | -1.335 | NA | NA | NA | NA | NA |
| Insulin:Quetiapine | 0 | -1.45827 | NA | -1.45827 | NA | NA | NA | NA |
| Insulin:Ramelteon | 0 | -1.3642 | NA | -1.3642 | NA | NA | NA | NA |
| Insulin:Risperidone | 0 | -0.28518 | NA | -0.28518 | NA | NA | NA | NA |
| Insulin:Rivastigmine | 0 | -1.16909 | NA | -1.16909 | NA | NA | NA | NA |
| Insulin:Simvastatin | 0 | -1.31996 | NA | -1.31996 | NA | NA | NA | NA |
| Insulin:Suvorexant | 0 | 1.229948 | NA | 1.229948 | NA | NA | NA | NA |
| Insulin:Thiamine | 0 | -1.4993 | NA | -1.4993 | NA | NA | NA | NA |
| Ketamine_high_dosage:Ketamine_low_dosage | 1 | 0.179939 | 0.179046 | 0.184117 | -0.00507 | -1.83594 | 1.825803 | 0.995669 |
| Ketamine_high_dosage:L_tryptophan | 0 | -0.37163 | NA | -0.37163 | NA | NA | NA | NA |
| Ketamine_high_dosage:Lidocaine | 0 | 0.203717 | NA | 0.203717 | NA | NA | NA | NA |
| Ketamine_high_dosage:Melatonin | 0 | -0.15086 | NA | -0.15086 | NA | NA | NA | NA |
| Ketamine_high_dosage:Methylprednisolone | 0 | 0.332613 | NA | 0.332613 | NA | NA | NA | NA |
| Ketamine_high_dosage:Minocycline | 0 | -0.85291 | NA | -0.85291 | NA | NA | NA | NA |
| Ketamine_high_dosage:Neostigmine | 0 | -0.2093 | NA | -0.2093 | NA | NA | NA | NA |
| Ketamine_high_dosage:Olanzapine | 0 | 0.746488 | NA | 0.746488 | NA | NA | NA | NA |
| Ketamine_high_dosage:Ondansetron | 0 | 0.113395 | NA | 0.113395 | NA | NA | NA | NA |
| Ketamine_high_dosage:Parecoxib | 0 | 0.2939 | NA | 0.2939 | NA | NA | NA | NA |
| Ketamine_high_dosage:Physostigmine | 0 | -0.55805 | NA | -0.55805 | NA | NA | NA | NA |
| Ketamine_high_dosage:Placebo | 3 | -0.28802 | -0.22758 | -1.70613 | 1.478546 | -1.15226 | 4.109356 | 0.270669 |
| Ketamine_high_dosage:Quetiapine | 0 | -0.41129 | NA | -0.41129 | NA | NA | NA | NA |
| Ketamine_high_dosage:Ramelteon | 0 | -0.31722 | NA | -0.31722 | NA | NA | NA | NA |
| Ketamine_high_dosage:Risperidone | 0 | 0.7618 | NA | 0.7618 | NA | NA | NA | NA |
| Ketamine_high_dosage:Rivastigmine | 0 | -0.12211 | NA | -0.12211 | NA | NA | NA | NA |
| Ketamine_high_dosage:Simvastatin | 0 | -0.27298 | NA | -0.27298 | NA | NA | NA | NA |
| Ketamine_high_dosage:Suvorexant | 0 | 2.276928 | NA | 2.276928 | NA | NA | NA | NA |
| Ketamine_high_dosage:Thiamine | 0 | -0.45232 | NA | -0.45232 | NA | NA | NA | NA |
| Ketamine_low_dosage:L_tryptophan | 0 | -0.55157 | NA | -0.55157 | NA | NA | NA | NA |
| Ketamine_low_dosage:Lidocaine | 0 | 0.023777 | NA | 0.023777 | NA | NA | NA | NA |
| Ketamine_low_dosage:Melatonin | 0 | -0.33079 | NA | -0.33079 | NA | NA | NA | NA |
| Ketamine_low_dosage:Methylprednisolone | 0 | 0.152674 | NA | 0.152674 | NA | NA | NA | NA |
| Ketamine_low_dosage:Minocycline | 0 | -1.03285 | NA | -1.03285 | NA | NA | NA | NA |
| Ketamine_low_dosage:Neostigmine | 0 | -0.38924 | NA | -0.38924 | NA | NA | NA | NA |
| Ketamine_low_dosage:Olanzapine | 0 | 0.566548 | NA | 0.566548 | NA | NA | NA | NA |
| Ketamine_low_dosage:Ondansetron | 0 | -0.06654 | NA | -0.06654 | NA | NA | NA | NA |
| Ketamine_low_dosage:Parecoxib | 0 | 0.11396 | NA | 0.11396 | NA | NA | NA | NA |
| Ketamine_low_dosage:Physostigmine | 0 | -0.73799 | NA | -0.73799 | NA | NA | NA | NA |
| Ketamine_low_dosage:Placebo | 2 | -0.46796 | -0.36313 | -1.25774 | 0.89461 | -1.22295 | 3.012172 | 0.407654 |
| Ketamine_low_dosage:Quetiapine | 0 | -0.59123 | NA | -0.59123 | NA | NA | NA | NA |
| Ketamine_low_dosage:Ramelteon | 0 | -0.49716 | NA | -0.49716 | NA | NA | NA | NA |
| Ketamine_low_dosage:Risperidone | 0 | 0.581861 | NA | 0.581861 | NA | NA | NA | NA |
| Ketamine_low_dosage:Rivastigmine | 0 | -0.30205 | NA | -0.30205 | NA | NA | NA | NA |
| Ketamine_low_dosage:Simvastatin | 0 | -0.45292 | NA | -0.45292 | NA | NA | NA | NA |
| Ketamine_low_dosage:Suvorexant | 0 | 2.096988 | NA | 2.096988 | NA | NA | NA | NA |
| Ketamine_low_dosage:Thiamine | 0 | -0.63226 | NA | -0.63226 | NA | NA | NA | NA |
| L_tryptophan:Lidocaine | 0 | 0.575345 | NA | 0.575345 | NA | NA | NA | NA |
| L_tryptophan:Melatonin | 0 | 0.220773 | NA | 0.220773 | NA | NA | NA | NA |
| L_tryptophan:Methylprednisolone | 0 | 0.704241 | NA | 0.704241 | NA | NA | NA | NA |
| L_tryptophan:Minocycline | 0 | -0.48129 | NA | -0.48129 | NA | NA | NA | NA |
| L_tryptophan:Neostigmine | 0 | 0.162325 | NA | 0.162325 | NA | NA | NA | NA |
| L_tryptophan:Olanzapine | 0 | 1.118116 | NA | 1.118116 | NA | NA | NA | NA |
| L_tryptophan:Ondansetron | 0 | 0.485023 | NA | 0.485023 | NA | NA | NA | NA |
| L_tryptophan:Parecoxib | 0 | 0.665528 | NA | 0.665528 | NA | NA | NA | NA |
| L_tryptophan:Physostigmine | 0 | -0.18642 | NA | -0.18642 | NA | NA | NA | NA |
| L_tryptophan:Placebo | 1 | 0.083606 | 0.083606 | NA | NA | NA | NA | NA |
| L_tryptophan:Quetiapine | 0 | -0.03966 | NA | -0.03966 | NA | NA | NA | NA |
| L_tryptophan:Ramelteon | 0 | 0.054412 | NA | 0.054412 | NA | NA | NA | NA |
| L_tryptophan:Risperidone | 0 | 1.133429 | NA | 1.133429 | NA | NA | NA | NA |
| L_tryptophan:Rivastigmine | 0 | 0.249519 | NA | 0.249519 | NA | NA | NA | NA |
| L_tryptophan:Simvastatin | 0 | 0.098644 | NA | 0.098644 | NA | NA | NA | NA |
| L_tryptophan:Suvorexant | 0 | 2.648556 | NA | 2.648556 | NA | NA | NA | NA |
| L_tryptophan:Thiamine | 0 | -0.0807 | NA | -0.0807 | NA | NA | NA | NA |
| Lidocaine:Melatonin | 0 | -0.35457 | NA | -0.35457 | NA | NA | NA | NA |
| Lidocaine:Methylprednisolone | 0 | 0.128897 | NA | 0.128897 | NA | NA | NA | NA |
| Lidocaine:Minocycline | 0 | -1.05663 | NA | -1.05663 | NA | NA | NA | NA |
| Lidocaine:Neostigmine | 0 | -0.41302 | NA | -0.41302 | NA | NA | NA | NA |
| Lidocaine:Olanzapine | 0 | 0.542771 | NA | 0.542771 | NA | NA | NA | NA |
| Lidocaine:Ondansetron | 0 | -0.09032 | NA | -0.09032 | NA | NA | NA | NA |
| Lidocaine:Parecoxib | 0 | 0.090183 | NA | 0.090183 | NA | NA | NA | NA |
| Lidocaine:Physostigmine | 0 | -0.76177 | NA | -0.76177 | NA | NA | NA | NA |
| Lidocaine:Placebo | 0 | -0.49174 | NA | -0.49174 | NA | NA | NA | NA |
| Lidocaine:Quetiapine | 0 | -0.61501 | NA | -0.61501 | NA | NA | NA | NA |
| Lidocaine:Ramelteon | 0 | -0.52093 | NA | -0.52093 | NA | NA | NA | NA |
| Lidocaine:Risperidone | 0 | 0.558084 | NA | 0.558084 | NA | NA | NA | NA |
| Lidocaine:Rivastigmine | 0 | -0.32583 | NA | -0.32583 | NA | NA | NA | NA |
| Lidocaine:Simvastatin | 0 | -0.4767 | NA | -0.4767 | NA | NA | NA | NA |
| Lidocaine:Suvorexant | 0 | 2.073211 | NA | 2.073211 | NA | NA | NA | NA |
| Lidocaine:Thiamine | 0 | -0.65604 | NA | -0.65604 | NA | NA | NA | NA |
| Melatonin:Methylprednisolone | 0 | 0.483469 | NA | 0.483469 | NA | NA | NA | NA |
| Melatonin:Minocycline | 0 | -0.70206 | NA | -0.70206 | NA | NA | NA | NA |
| Melatonin:Neostigmine | 0 | -0.05845 | NA | -0.05845 | NA | NA | NA | NA |
| Melatonin:Olanzapine | 0 | 0.897343 | NA | 0.897343 | NA | NA | NA | NA |
| Melatonin:Ondansetron | 0 | 0.26425 | NA | 0.26425 | NA | NA | NA | NA |
| Melatonin:Parecoxib | 0 | 0.444755 | NA | 0.444755 | NA | NA | NA | NA |
| Melatonin:Physostigmine | 0 | -0.40719 | NA | -0.40719 | NA | NA | NA | NA |
| Melatonin:Placebo | 8 | -0.13717 | -0.13717 | NA | NA | NA | NA | NA |
| Melatonin:Quetiapine | 0 | -0.26044 | NA | -0.26044 | NA | NA | NA | NA |
| Melatonin:Ramelteon | 0 | -0.16636 | NA | -0.16636 | NA | NA | NA | NA |
| Melatonin:Risperidone | 0 | 0.912656 | NA | 0.912656 | NA | NA | NA | NA |
| Melatonin:Rivastigmine | 0 | 0.028747 | NA | 0.028747 | NA | NA | NA | NA |
| Melatonin:Simvastatin | 0 | -0.12213 | NA | -0.12213 | NA | NA | NA | NA |
| Melatonin:Suvorexant | 0 | 2.427783 | NA | 2.427783 | NA | NA | NA | NA |
| Melatonin:Thiamine | 0 | -0.30147 | NA | -0.30147 | NA | NA | NA | NA |
| Methylprednisolone:Minocycline | 0 | -1.18553 | NA | -1.18553 | NA | NA | NA | NA |
| Methylprednisolone:Neostigmine | 0 | -0.54192 | NA | -0.54192 | NA | NA | NA | NA |
| Methylprednisolone:Olanzapine | 0 | 0.413874 | NA | 0.413874 | NA | NA | NA | NA |
| Methylprednisolone:Ondansetron | 0 | -0.21922 | NA | -0.21922 | NA | NA | NA | NA |
| Methylprednisolone:Parecoxib | 0 | -0.03871 | NA | -0.03871 | NA | NA | NA | NA |
| Methylprednisolone:Physostigmine | 0 | -0.89066 | NA | -0.89066 | NA | NA | NA | NA |
| Methylprednisolone:Placebo | 3 | -0.62064 | -0.55171 | -2.72455 | 2.172841 | -0.84606 | 5.191739 | 0.15834 |
| Methylprednisolone:Quetiapine | 0 | -0.7439 | NA | -0.7439 | NA | NA | NA | NA |
| Methylprednisolone:Ramelteon | 0 | -0.64983 | NA | -0.64983 | NA | NA | NA | NA |
| Methylprednisolone:Risperidone | 0 | 0.429187 | NA | 0.429187 | NA | NA | NA | NA |
| Methylprednisolone:Rivastigmine | 0 | -0.45472 | NA | -0.45472 | NA | NA | NA | NA |
| Methylprednisolone:Simvastatin | 0 | -0.6056 | NA | -0.6056 | NA | NA | NA | NA |
| Methylprednisolone:Suvorexant | 0 | 1.944314 | NA | 1.944314 | NA | NA | NA | NA |
| Methylprednisolone:Thiamine | 0 | -0.78494 | NA | -0.78494 | NA | NA | NA | NA |
| Minocycline:Neostigmine | 0 | 0.643611 | NA | 0.643611 | NA | NA | NA | NA |
| Minocycline:Olanzapine | 0 | 1.599402 | NA | 1.599402 | NA | NA | NA | NA |
| Minocycline:Ondansetron | 0 | 0.966309 | NA | 0.966309 | NA | NA | NA | NA |
| Minocycline:Parecoxib | 0 | 1.146814 | NA | 1.146814 | NA | NA | NA | NA |
| Minocycline:Physostigmine | 0 | 0.294866 | NA | 0.294866 | NA | NA | NA | NA |
| Minocycline:Placebo | 1 | 0.564893 | 0.564893 | NA | NA | NA | NA | NA |
| Minocycline:Quetiapine | 0 | 0.441623 | NA | 0.441623 | NA | NA | NA | NA |
| Minocycline:Ramelteon | 0 | 0.535699 | NA | 0.535699 | NA | NA | NA | NA |
| Minocycline:Risperidone | 0 | 1.614715 | NA | 1.614715 | NA | NA | NA | NA |
| Minocycline:Rivastigmine | 0 | 0.730806 | NA | 0.730806 | NA | NA | NA | NA |
| Minocycline:Simvastatin | 0 | 0.579931 | NA | 0.579931 | NA | NA | NA | NA |
| Minocycline:Suvorexant | 0 | 3.129842 | NA | 3.129842 | NA | NA | NA | NA |
| Minocycline:Thiamine | 0 | 0.40059 | NA | 0.40059 | NA | NA | NA | NA |
| Neostigmine:Olanzapine | 0 | 0.955791 | NA | 0.955791 | NA | NA | NA | NA |
| Neostigmine:Ondansetron | 0 | 0.322698 | NA | 0.322698 | NA | NA | NA | NA |
| Neostigmine:Parecoxib | 0 | 0.503203 | NA | 0.503203 | NA | NA | NA | NA |
| Neostigmine:Physostigmine | 0 | -0.34875 | NA | -0.34875 | NA | NA | NA | NA |
| Neostigmine:Placebo | 1 | -0.07872 | -0.07872 | NA | NA | NA | NA | NA |
| Neostigmine:Quetiapine | 0 | -0.20199 | NA | -0.20199 | NA | NA | NA | NA |
| Neostigmine:Ramelteon | 0 | -0.10791 | NA | -0.10791 | NA | NA | NA | NA |
| Neostigmine:Risperidone | 0 | 0.971103 | NA | 0.971103 | NA | NA | NA | NA |
| Neostigmine:Rivastigmine | 0 | 0.087194 | NA | 0.087194 | NA | NA | NA | NA |
| Neostigmine:Simvastatin | 0 | -0.06368 | NA | -0.06368 | NA | NA | NA | NA |
| Neostigmine:Suvorexant | 0 | 2.486231 | NA | 2.486231 | NA | NA | NA | NA |
| Neostigmine:Thiamine | 0 | -0.24302 | NA | -0.24302 | NA | NA | NA | NA |
| Olanzapine:Ondansetron | 0 | -0.63309 | NA | -0.63309 | NA | NA | NA | NA |
| Olanzapine:Parecoxib | 0 | -0.45259 | NA | -0.45259 | NA | NA | NA | NA |
| Olanzapine:Physostigmine | 0 | -1.30454 | NA | -1.30454 | NA | NA | NA | NA |
| Olanzapine:Placebo | 1 | -1.03451 | -1.03451 | NA | NA | NA | NA | NA |
| Olanzapine:Quetiapine | 0 | -1.15778 | NA | -1.15778 | NA | NA | NA | NA |
| Olanzapine:Ramelteon | 0 | -1.0637 | NA | -1.0637 | NA | NA | NA | NA |
| Olanzapine:Risperidone | 0 | 0.015313 | NA | 0.015313 | NA | NA | NA | NA |
| Olanzapine:Rivastigmine | 0 | -0.8686 | NA | -0.8686 | NA | NA | NA | NA |
| Olanzapine:Simvastatin | 0 | -1.01947 | NA | -1.01947 | NA | NA | NA | NA |
| Olanzapine:Suvorexant | 0 | 1.53044 | NA | 1.53044 | NA | NA | NA | NA |
| Olanzapine:Thiamine | 0 | -1.19881 | NA | -1.19881 | NA | NA | NA | NA |
| Ondansetron:Parecoxib | 0 | 0.180505 | NA | 0.180505 | NA | NA | NA | NA |
| Ondansetron:Physostigmine | 0 | -0.67144 | NA | -0.67144 | NA | NA | NA | NA |
| Ondansetron:Placebo | 1 | -0.40142 | -0.40142 | NA | NA | NA | NA | NA |
| Ondansetron:Quetiapine | 0 | -0.52469 | NA | -0.52469 | NA | NA | NA | NA |
| Ondansetron:Ramelteon | 0 | -0.43061 | NA | -0.43061 | NA | NA | NA | NA |
| Ondansetron:Risperidone | 0 | 0.648406 | NA | 0.648406 | NA | NA | NA | NA |
| Ondansetron:Rivastigmine | 0 | -0.2355 | NA | -0.2355 | NA | NA | NA | NA |
| Ondansetron:Simvastatin | 0 | -0.38638 | NA | -0.38638 | NA | NA | NA | NA |
| Ondansetron:Suvorexant | 0 | 2.163533 | NA | 2.163533 | NA | NA | NA | NA |
| Ondansetron:Thiamine | 0 | -0.56572 | NA | -0.56572 | NA | NA | NA | NA |
| Parecoxib:Physostigmine | 0 | -0.85195 | NA | -0.85195 | NA | NA | NA | NA |
| Parecoxib:Placebo | 1 | -0.58192 | -0.58192 | NA | NA | NA | NA | NA |
| Parecoxib:Quetiapine | 0 | -0.70519 | NA | -0.70519 | NA | NA | NA | NA |
| Parecoxib:Ramelteon | 0 | -0.61112 | NA | -0.61112 | NA | NA | NA | NA |
| Parecoxib:Risperidone | 0 | 0.467901 | NA | 0.467901 | NA | NA | NA | NA |
| Parecoxib:Rivastigmine | 0 | -0.41601 | NA | -0.41601 | NA | NA | NA | NA |
| Parecoxib:Simvastatin | 0 | -0.56688 | NA | -0.56688 | NA | NA | NA | NA |
| Parecoxib:Suvorexant | 0 | 1.983028 | NA | 1.983028 | NA | NA | NA | NA |
| Parecoxib:Thiamine | 0 | -0.74622 | NA | -0.74622 | NA | NA | NA | NA |
| Physostigmine:Placebo | 1 | 0.270027 | 0.270027 | NA | NA | NA | NA | NA |
| Physostigmine:Quetiapine | 0 | 0.146757 | NA | 0.146757 | NA | NA | NA | NA |
| Physostigmine:Ramelteon | 0 | 0.240833 | NA | 0.240833 | NA | NA | NA | NA |
| Physostigmine:Risperidone | 0 | 1.319849 | NA | 1.319849 | NA | NA | NA | NA |
| Physostigmine:Rivastigmine | 0 | 0.43594 | NA | 0.43594 | NA | NA | NA | NA |
| Physostigmine:Simvastatin | 0 | 0.285065 | NA | 0.285065 | NA | NA | NA | NA |
| Physostigmine:Suvorexant | 0 | 2.834976 | NA | 2.834976 | NA | NA | NA | NA |
| Physostigmine:Thiamine | 0 | 0.105724 | NA | 0.105724 | NA | NA | NA | NA |
| Placebo:Quetiapine | 1 | -0.12327 | -0.47 | 0.061494 | -0.5315 | -2.07804 | 1.015048 | 0.500581 |
| Placebo:Ramelteon | 4 | -0.02919 | -0.02919 | NA | NA | NA | NA | NA |
| Placebo:Risperidone | 1 | 1.049822 | 1.049822 | NA | NA | NA | NA | NA |
| Placebo:Rivastigmine | 2 | 0.165913 | -0.00362 | 0.567545 | -0.57116 | -1.98037 | 0.838046 | 0.42697 |
| Placebo:Simvastatin | 1 | 0.015038 | 0.015038 | NA | NA | NA | NA | NA |
| Placebo:Suvorexant | 1 | 2.564949 | 2.564949 | NA | NA | NA | NA | NA |
| Placebo:Thiamine | 1 | -0.1643 | -0.1643 | NA | NA | NA | NA | NA |
| Quetiapine:Ramelteon | 0 | 0.094076 | NA | 0.094076 | NA | NA | NA | NA |
| Quetiapine:Risperidone | 0 | 1.173092 | NA | 1.173092 | NA | NA | NA | NA |
| Quetiapine:Rivastigmine | 0 | 0.289183 | NA | 0.289183 | NA | NA | NA | NA |
| Quetiapine:Simvastatin | 0 | 0.138308 | NA | 0.138308 | NA | NA | NA | NA |
| Quetiapine:Suvorexant | 0 | 2.688219 | NA | 2.688219 | NA | NA | NA | NA |
| Quetiapine:Thiamine | 0 | -0.04103 | NA | -0.04103 | NA | NA | NA | NA |
| Ramelteon:Risperidone | 0 | 1.079016 | NA | 1.079016 | NA | NA | NA | NA |
| Ramelteon:Rivastigmine | 0 | 0.195107 | NA | 0.195107 | NA | NA | NA | NA |
| Ramelteon:Simvastatin | 0 | 0.044232 | NA | 0.044232 | NA | NA | NA | NA |
| Ramelteon:Suvorexant | 0 | 2.594144 | NA | 2.594144 | NA | NA | NA | NA |
| Ramelteon:Thiamine | 0 | -0.13511 | NA | -0.13511 | NA | NA | NA | NA |
| Risperidone:Rivastigmine | 0 | -0.88391 | NA | -0.88391 | NA | NA | NA | NA |
| Risperidone:Simvastatin | 0 | -1.03478 | NA | -1.03478 | NA | NA | NA | NA |
| Risperidone:Suvorexant | 0 | 1.515127 | NA | 1.515127 | NA | NA | NA | NA |
| Risperidone:Thiamine | 0 | -1.21413 | NA | -1.21413 | NA | NA | NA | NA |
| Rivastigmine:Simvastatin | 0 | -0.15087 | NA | -0.15087 | NA | NA | NA | NA |
| Rivastigmine:Suvorexant | 0 | 2.399037 | NA | 2.399037 | NA | NA | NA | NA |
| Rivastigmine:Thiamine | 0 | -0.33022 | NA | -0.33022 | NA | NA | NA | NA |
| Simvastatin:Suvorexant | 0 | 2.549911 | NA | 2.549911 | NA | NA | NA | NA |
| Simvastatin:Thiamine | 0 | -0.17934 | NA | -0.17934 | NA | NA | NA | NA |
| Suvorexant:Thiamine | 0 | -2.72925 | NA | -2.72925 | NA | NA | NA | NA |

Abbreviation: NA: not applicable; NMA: network meta-analysis

**Reference list of supplement tables:**

1. Page MJ, McKenzie JE, Bossuyt PM *et al.* The PRISMA 2020 statement: an updated guideline for reporting systematic reviews. *Bmj.* 2021; **372**: n71.

2. Aizawa K, Kanai T, Saikawa Y *et al.* A novel approach to the prevention of postoperative delirium in the elderly after gastrointestinal surgery. *Surg Today.* 2002; **32**: 310-314.

3. Al-Qadheeb NS, Skrobik Y, Schumaker G *et al.* Preventing ICU Subsyndromal Delirium Conversion to Delirium With Low-Dose IV Haloperidol: A Double-Blind, Placebo-Controlled Pilot Study. *Crit Care Med.* 2016; **44**: 583-591.

4. Bartoszek M, McGuire JM, Wilson JT, Sorensen JS, Vice TFR, Hudson AJ. The Effectiveness of Dexmedetomidine as a Prophylactic Treatment for Emergence Delirium Among Combat Veterans With High Anxiety: A Randomized Placebo-Controlled Trial. *Mil Med.* 2023; **188**: e286-e294.

5. Chen J, Xie S, Chen Y, Qiu T, Lin J. Effect of Preoperative Oral Saline Administration on Postoperative Delirium in Older Persons: A Randomized Controlled Trial. *Clin Interv Aging.* 2022; **17**: 1539-1548.

6. Fukata S, Kawabata Y, Fujisiro K *et al.* Haloperidol prophylaxis does not prevent postoperative delirium in elderly patients: a randomized, open-label prospective trial. *Surg Today.* 2014; **44**: 2305-2313.

7. Fukata S, Kawabata Y, Fujishiro K *et al.* Haloperidol prophylaxis for preventing aggravation of postoperative delirium in elderly patients: a randomized, open-label prospective trial. *Surg Today.* 2017; **47**: 815-826.

8. Kawazoe Y, Miyamoto K, Morimoto T *et al.* Effect of Dexmedetomidine on Mortality and Ventilator-Free Days in Patients Requiring Mechanical Ventilation With Sepsis: A Randomized Clinical Trial. *Jama.* 2017; **317**: 1321-1328.

9. Levanen J, Makela ML, Scheinin H. Dexmedetomidine premedication attenuates ketamine-induced cardiostimulatory effects and postanesthetic delirium. *Anesthesiology.* 1995; **82**: 1117-1125.

10. Niu JY, Yang N, Tao QY *et al.* Effect of Different Administration Routes of Dexmedetomidine on Postoperative Delirium in Elderly Patients Undergoing Elective Spinal Surgery: A Prospective Randomized Double-Blinded Controlled Trial. *Anesth Analg.* 2023; **136**: 1075-1083.

11. Qiu Z, Zhou S, Zhang M *et al.* Preventive effect of dexmedetomidine on postictal delirium after electroconvulsive therapy: A randomised controlled study. *Eur J Anaesthesiol.* 2020; **37**: 5-13.

12. Rood PJT, Zegers M, Slooter AJC *et al.* Prophylactic Haloperidol Effects on Long-term Quality of Life in Critically Ill Patients at High Risk for Delirium: Results of the REDUCE Study. *Anesthesiology.* 2019; **131**: 328-335.

13. Shehabi Y, Howe BD, Bellomo R *et al.* Early Sedation with Dexmedetomidine in Critically Ill Patients. *N Engl J Med.* 2019; **380**: 2506-2517.

14. Siripoonyothai S, Sindhvananda W. Comparison of postoperative delirium within 24 hours between ketamine and propofol infusion during cardiopulmonary bypass machine: A randomized controlled trial. *Annals of cardiac anaesthesia.* 2021; **24**: 294-301.

15. Subramaniam B, Shankar P, Shaefi S *et al.* Effect of Intravenous Acetaminophen vs Placebo Combined With Propofol or Dexmedetomidine on Postoperative Delirium Among Older Patients Following Cardiac Surgery: The DEXACET Randomized Clinical Trial. *Jama.* 2019; **321**: 686-696.

16. Tang C, Hu Y, Zhang Z *et al.* Dexmedetomidine with sufentanil in intravenous patient-controlled analgesia for relief from postoperative pain, inflammation and delirium after esophageal cancer surgery. *Biosci Rep.* 2020; **40**.

17. Wang HB, Jia Y, Zhang CB *et al.* A randomised controlled trial of dexmedetomidine for delirium in adults undergoing heart valve surgery. *Anaesthesia.* 2023; **78**: 571-576.

18. Wong J, Doherty HR, Singh M *et al.* The prevention of delirium in elderly surgical patients with obstructive sleep apnea (PODESA): a randomized controlled trial. *BMC Anesthesiol.* 2022; **22**: 290.

19. Xin X, Huo SP, Zhang Q, Li YN, Wang L, Wang QJ. [Effects of preconditioning with hypertonic saline solution on postoperative delirium in the aged]. *Zhonghua yi xue za zhi.* 2017; **97**: 3072-3078.

20. Xin X, Xin F, Chen X *et al.* Hypertonic saline for prevention of delirium in geriatric patients who underwent hip surgery. *Journal of neuroinflammation.* 2017; **14**: 221.

21. Huang JW, Yang YF, Gao XS, Xu ZH. A single preoperative low-dose dexamethasone may reduce the incidence and severity of postoperative delirium in the geriatric intertrochanteric fracture patients with internal fixation surgery: an exploratory analysis of a randomized, placebo-controlled trial. *J Orthop Surg Res.* 2023; **18**: 441.

22. Kinouchi M, Mihara T, Taguri M, Ogura M. The Efficacy of Ramelteon to Prevent Postoperative Delirium After General Anesthesia in the Elderly: A Double-Blind, Randomized, Placebo-Controlled Trial. *Am J Geriatr Psychiatry.* 2023; **31**: 1178-1189.

23. Lai Y, Chen Q, Xiang C, Li G, Wei K. Comparison of the Effects of Dexmedetomidine and Lidocaine on Stress Response and Postoperative Delirium of Older Patients Undergoing Thoracoscopic Surgery: A Randomized Controlled Trial. *Clin Interv Aging.* 2023; **18**: 1275-1283.

24. Li S, Li R, Li M *et al.* Dexmedetomidine administration during brain tumour resection for prevention of postoperative delirium: a randomised trial. *Br J Anaesth.* 2023; **130**: e307-e316.

25. Takazawa T, Horiuchi T, Orihara M *et al.* Prevention of Postoperative Cognitive Dysfunction by Minocycline in Elderly Patients after Total Knee Arthroplasty: A Randomized, Double-blind, Placebo-controlled Clinical Trial. *Anesthesiology.* 2023; **138**: 172-183.

26. Xie K, Chen J, Tian L *et al.* Postoperative infusion of dexmedetomidine via intravenous patient-controlled analgesia for prevention of postoperative delirium in elderly patients undergoing surgery. *Aging Clin Exp Res.* 2023; **35**: 2137-2144.

27. Awada HN, Steinthorsdottir KJ, Schultz NA *et al.* High-dose preoperative glucocorticoid for prevention of emergence and postoperative delirium in liver resection: A double-blinded randomized clinical trial substudy. *Acta Anaesthesiol Scand.* 2022; **66**: 696-703.

28. Liu F, Lin X, Lin Y *et al.* The effect of neostigmine on postoperative delirium after colon carcinoma surgery: a randomized, double-blind, controlled trial. *BMC Anesthesiol.* 2022; **22**: 267.

29. Lv Y, Gu L. Dexmedetomidine potential in attenuating postoperative delirium in elderly patients after total hip joint replacement. *Rev Assoc Med Bras (1992).* 2022; **68**: 1166-1171.

30. Tang Y, Wang Y, Kong G, Zhao Y, Wei L, Liu J. Prevention of dexmedetomidine on postoperative delirium and early postoperative cognitive dysfunction in elderly patients undergoing hepatic lobectomy. *Zhong Nan Da Xue Xue Bao Yi Xue Ban.* 2022; **47**: 219-225.

31. Wibrow B, Martinez FE, Myers E *et al.* Prophylactic melatonin for delirium in intensive care (Pro-MEDIC): a randomized controlled trial. *Intensive Care Med.* 2022; **48**: 414-425.

32. Xiang XB, Chen H, Wu YL, Wang K, Yue X, Cheng XQ. The Effect of Preoperative Methylprednisolone on Postoperative Delirium in Older Patients Undergoing Gastrointestinal Surgery: A Randomized, Double-Blind, Placebo-Controlled Trial. *J Gerontol A Biol Sci Med Sci.* 2022; **77**: 517-523.

33. Abraham MP, Hinds M, Tayidi I *et al.* Quetiapine for delirium prophylaxis in high-risk critically ill patients. *Surgeon.* 2021; **19**: 65-71.

34. He X, Cheng KM, Duan YQ *et al.* Feasibility of low-dose dexmedetomidine for prevention of postoperative delirium after intracranial operations: a pilot randomized controlled trial. *BMC Neurol.* 2021; **21**: 472.

35. Hollinger A, Rust CA, Riegger H *et al.* Ketamine vs. haloperidol for prevention of cognitive dysfunction and postoperative delirium: A phase IV multicentre randomised placebo-controlled double-blind clinical trial. *J Clin Anesth.* 2021; **68**: 110099.

36. Hu J, Zhu M, Gao Z *et al.* Dexmedetomidine for prevention of postoperative delirium in older adults undergoing oesophagectomy with total intravenous anaesthesia: A double-blind, randomised clinical trial. *Eur J Anaesthesiol.* 2021; **38**: S9-S17.

37. Huang Q, Li Q, Qin F *et al.* Repeated Preoperative Intranasal Administration of Insulin Decreases the Incidence of Postoperative Delirium in Elderly Patients Undergoing Laparoscopic Radical Gastrointestinal Surgery: A Randomized, Placebo-Controlled, Double-Blinded Clinical Study. *Am J Geriatr Psychiatry.* 2021; **29**: 1202-1211.

38. Javaherforoosh Zadeh F, Janatmakan F, Shafaeebejestan E, Jorairahmadi S. Effect of Melatonin on Delirium After on-Pump Coronary Artery Bypass Graft Surgery: A Randomized Clinical Trial. *Iran J Med Sci.* 2021; **46**: 120-127.

39. Kluger MT, Skarin M, Collier J *et al.* Steroids to reduce the impact on delirium (STRIDE): a double-blind, randomised, placebo-controlled feasibility trial of pre-operative dexamethasone in people with hip fracture. *Anaesthesia.* 2021; **76**: 1031-1041.

40. Likhvantsev VV, Landoni G, Grebenchikov OA *et al.* Perioperative Dexmedetomidine Supplement Decreases Delirium Incidence After Adult Cardiac Surgery: A Randomized, Double-Blind, Controlled Study. *Journal of cardiothoracic and vascular anesthesia.* 2021; **35**: 449-457.

41. Momeni M, Khalifa C, Lemaire G *et al.* Propofol plus low-dose dexmedetomidine infusion and postoperative delirium in older patients undergoing cardiac surgery. *Br J Anaesth.* 2021; **126**: 665-673.

42. Nakamura ZM, Deal AM, Park EM *et al.* A randomized double-blind placebo-controlled trial of intravenous thiamine for prevention of delirium following allogeneic hematopoietic stem cell transplantation. *Journal of psychosomatic research.* 2021; **146**: 110503.

43. Oh ES, Leoutsakos JM, Rosenberg PB *et al.* Effects of Ramelteon on the Prevention of Postoperative Delirium in Older Patients Undergoing Orthopedic Surgery: The RECOVER Randomized Controlled Trial. *Am J Geriatr Psychiatry.* 2021; **29**: 90-100.

44. Shi Y. Effects of Melatonin on Postoperative Delirium After PCI in Elderly Patients: A Randomized, Single-Center, Double-Blind, Placebo-Controlled Trial. *Heart Surg Forum.* 2021; **24**: E893-E897.

45. Spies CD, Knaak C, Mertens M *et al.* Physostigmine for prevention of postoperative delirium and long-term cognitive dysfunction in liver surgery: A double-blinded randomised controlled trial. *Eur J Anaesthesiol.* 2021; **38**: 943-956.

46. Thanapluetiwong S, Ruangritchankul S, Sriwannopas O *et al.* Efficacy of quetiapine for delirium prevention in hospitalized older medical patients: a randomized double-blind controlled trial. *BMC Geriatr.* 2021; **21**: 215.

47. van Norden J, Spies CD, Borchers F *et al.* The effect of peri-operative dexmedetomidine on the incidence of postoperative delirium in cardiac and non-cardiac surgical patients: a randomised, double-blind placebo-controlled trial. *Anaesthesia.* 2021; **76**: 1342-1351.

48. Ford AH, Flicker L, Kelly R *et al.* The Healthy Heart-Mind Trial: Randomized Controlled Trial of Melatonin for Prevention of Delirium. *J Am Geriatr Soc.* 2020; **68**: 112-119.

49. Lawlor PG, McNamara-Kilian MT, MacDonald AR *et al.* Melatonin to prevent delirium in patients with advanced cancer: a double blind, parallel, randomized, controlled, feasibility trial. *BMC Palliat Care.* 2020; **19**: 163.

50. Lee H, Yang SM, Chung J *et al.* Effect of Perioperative Low-Dose Dexmedetomidine on Postoperative Delirium After Living-Donor Liver Transplantation: A Randomized Controlled Trial. *Transplant Proc.* 2020; **52**: 239-245.

51. Li CJ, Wang BJ, Mu DL *et al.* Randomized clinical trial of intraoperative dexmedetomidine to prevent delirium in the elderly undergoing major non-cardiac surgery. *Br J Surg.* 2020; **107**: e123-e132.

52. Mokhtari M, Farasatinasab M, Jafarpour Machian M *et al.* Aripiprazole for prevention of delirium in the neurosurgical intensive care unit: a double-blind, randomized, placebo-controlled study. *Eur J Clin Pharmacol.* 2020; **76**: 491-499.

53. Shokri H, Ali I. A randomized control trial comparing prophylactic dexmedetomidine versus clonidine on rates and duration of delirium in older adult patients undergoing coronary artery bypass grafting. *J Clin Anesth.* 2020; **61**: 109622.

54. Turan A, Duncan A, Leung S *et al.* Dexmedetomidine for reduction of atrial fibrillation and delirium after cardiac surgery (DECADE): a randomised placebo-controlled trial. *Lancet.* 2020; **396**: 177-185.

55. Jaiswal SJ, Vyas AD, Heisel AJ *et al.* Ramelteon for Prevention of Postoperative Delirium: A Randomized Controlled Trial in Patients Undergoing Elective Pulmonary Thromboendarterectomy. *Crit Care Med.* 2019; **47**: 1751-1758.

56. Kim JA, Ahn HJ, Yang M, Lee SH, Jeong H, Seong BG. Intraoperative use of dexmedetomidine for the prevention of emergence agitation and postoperative delirium in thoracic surgery: a randomized-controlled trial. *Can J Anaesth.* 2019; **66**: 371-379.

57. Li Y, Yu ZX, Ji MS *et al.* A Pilot Study of the Use of Dexmedetomidine for the Control of Delirium by Reducing the Serum Concentrations of Brain-Derived Neurotrophic Factor, Neuron-Specific Enolase, and S100B in Polytrauma Patients. *Journal of intensive care medicine.* 2019; **34**: 674-681.

58. Wang X, Wang Y, Hu Y *et al.* Effect of flurbiprofen axetil on postoperative delirium for elderly patients. *Brain Behav.* 2019; **9**: e01290.

59. Clemmesen CG, Lunn TH, Kristensen MT, Palm H, Foss NB. Effect of a single pre-operative 125 mg dose of methylprednisolone on postoperative delirium in hip fracture patients; a randomised, double-blind, placebo-controlled trial. *Anaesthesia.* 2018; **73**: 1353-1360.

60. Jaiswal SJ, McCarthy TJ, Wineinger NE *et al.* Melatonin and Sleep in Preventing Hospitalized Delirium: A Randomized Clinical Trial. *The American journal of medicine.* 2018; **131**: 1110-1117 e1114.

61. Khan BA, Perkins AJ, Campbell NL *et al.* Preventing Postoperative Delirium After Major Noncardiac Thoracic Surgery-A Randomized Clinical Trial. *J Am Geriatr Soc.* 2018; **66**: 2289-2297.

62. Lee C, Lee CH, Lee G, Lee M, Hwang J. The effect of the timing and dose of dexmedetomidine on postoperative delirium in elderly patients after laparoscopic major non-cardiac surgery: A double blind randomized controlled study. *J Clin Anesth.* 2018; **47**: 27-32.

63. Perbet S, Verdonk F, Godet T *et al.* Low doses of ketamine reduce delirium but not opiate consumption in mechanically ventilated and sedated ICU patients: A randomised double-blind control trial. *Anaesth Crit Care Pain Med.* 2018; **37**: 589-595.

64. Schrijver EJM, de Vries OJ, van de Ven PM *et al.* Haloperidol versus placebo for delirium prevention in acutely hospitalised older at risk patients: a multi-centre double-blind randomised controlled clinical trial. *Age and ageing.* 2018; **47**: 48-55.

65. Skrobik Y, Duprey MS, Hill NS, Devlin JW. Low-Dose Nocturnal Dexmedetomidine Prevents ICU Delirium. A Randomized, Placebo-controlled Trial. *Am J Respir Crit Care Med.* 2018; **197**: 1147-1156.

66. van den Boogaard M, Slooter AJC, Bruggemann RJM *et al.* Effect of Haloperidol on Survival Among Critically Ill Adults With a High Risk of Delirium: The REDUCE Randomized Clinical Trial. *Jama.* 2018; **319**: 680-690.

67. Avidan MS, Maybrier HR, Abdallah AB *et al.* Intraoperative ketamine for prevention of postoperative delirium or pain after major surgery in older adults: an international, multicentre, double-blind, randomised clinical trial. *Lancet.* 2017; **390**: 267-275.

68. Deiner S, Luo X, Lin HM *et al.* Intraoperative Infusion of Dexmedetomidine for Prevention of Postoperative Delirium and Cognitive Dysfunction in Elderly Patients Undergoing Major Elective Noncardiac Surgery: A Randomized Clinical Trial. *JAMA Surg.* 2017; **152**: e171505.

69. Hatta K, Kishi Y, Wada K *et al.* Preventive Effects of Suvorexant on Delirium: A Randomized Placebo-Controlled Trial. *The Journal of clinical psychiatry.* 2017; **78**: e970-e979.

70. Leung JM, Sands LP, Chen N *et al.* Perioperative Gabapentin Does Not Reduce Postoperative Delirium in Older Surgical Patients: A Randomized Clinical Trial. *Anesthesiology.* 2017; **127**: 633-644.

71. Li X, Yang J, Nie XL *et al.* Impact of dexmedetomidine on the incidence of delirium in elderly patients after cardiac surgery: A randomized controlled trial. *PloS one.* 2017; **12**: e0170757.

72. Mu DL, Zhang DZ, Wang DX *et al.* Parecoxib Supplementation to Morphine Analgesia Decreases Incidence of Delirium in Elderly Patients After Hip or Knee Replacement Surgery: A Randomized Controlled Trial. *Anesth Analg.* 2017; **124**: 1992-2000.

73. Page VJ, Casarin A, Ely EW *et al.* Evaluation of early administration of simvastatin in the prevention and treatment of delirium in critically ill patients undergoing mechanical ventilation (MoDUS): a randomised, double-blind, placebo-controlled trial. *Lancet Respir Med.* 2017; **5**: 727-737.

74. Royse CF, Saager L, Whitlock R *et al.* Impact of Methylprednisolone on Postoperative Quality of Recovery and Delirium in the Steroids in Cardiac Surgery Trial: A Randomized, Double-blind, Placebo-controlled Substudy. *Anesthesiology.* 2017; **126**: 223-233.

75. Youn YC, Shin HW, Choi BS, Kim S, Lee JY, Ha YC. Rivastigmine patch reduces the incidence of postoperative delirium in older patients with cognitive impairment. *Int J Geriatr Psychiatry.* 2017; **32**: 1079-1084.

76. Abdelgalel EF. Dexmedetomidine versus haloperidol for prevention of delirium during non-invasive mechanical ventilation. *Egypt J Anaesth.* 2016; **32**: 473–481.

77. Djaiani G, Silverton N, Fedorko L *et al.* Dexmedetomidine versus Propofol Sedation Reduces Delirium after Cardiac Surgery: A Randomized Controlled Trial. *Anesthesiology.* 2016; **124**: 362-368.

78. Liu Y, Ma L, Gao M, Guo W, Ma Y. Dexmedetomidine reduces postoperative delirium after joint replacement in elderly patients with mild cognitive impairment. *Aging Clin Exp Res.* 2016; **28**: 729-736.

79. Lu X, Li J, Li T *et al.* Clinical study of midazolam sequential with dexmedetomidine for agitated patients undergoing weaning to implement light sedation in intensive care unit. *Chin J Traumatol.* 2016; **19**: 94-96.

80. Mohammadi M, Ahmadi M, Khalili H, Cheraghchi H, Arbabi M. Cyproheptadine for the Prevention of Postoperative Delirium: A Pilot Study. *The Annals of pharmacotherapy.* 2016; **50**: 180-187.

81. Su X, Meng ZT, Wu XH *et al.* Dexmedetomidine for prevention of delirium in elderly patients after non-cardiac surgery: a randomised, double-blind, placebo-controlled trial. *Lancet.* 2016; **388**: 1893-1902.

82. Guo Y, Sun LL, Chen ZF, Li QF, Jiang H. [Preventive effect of dexmedetomidine on postoperative delirium in elderly patients with oral cancer]. *Shanghai Kou Qiang Yi Xue.* 2015; **24**: 236-239.

83. Song R, Li J, Dong C, Yang J. [A study of using dexmedetomidine in ventilator bundle treatment in an ICU]. *Zhonghua Wei Zhong Bing Ji Jiu Yi Xue.* 2015; **27**: 836-840.

84. Wang K, Li C, Shi J, Wei H. [Effects of patient-controlled intravenous analgesia with dexmedetomidine and sufentanil on postoperative cognition in elderly patients after spine surgery]. *Zhonghua yi xue za zhi.* 2015; **95**: 2437-2441.

85. Yang X, Li Z, Gao C, Liu R. Effect of dexmedetomidine on preventing agitation and delirium after microvascular free flap surgery: a randomized, double-blind, control study. *J Oral Maxillofac Surg.* 2015; **73**: 1065-1072.

86. de Jonghe A, van Munster BC, Goslings JC *et al.* Effect of melatonin on incidence of delirium among patients with hip fracture: a multicentre, double-blind randomized controlled trial. *CMAJ.* 2014; **186**: E547-556.

87. Dighe K, Clarke H, McCartney CJ, Wong CL. Perioperative gabapentin and delirium following total knee arthroplasty: a post-hoc analysis of a double-blind randomized placebo-controlled trial. *Can J Anaesth.* 2014; **61**: 1136-1137.

88. Hatta K, Kishi Y, Wada K *et al.* Preventive effects of ramelteon on delirium: a randomized placebo-controlled trial. *JAMA psychiatry.* 2014; **71**: 397-403.

89. Papadopoulos G, Pouangare M, Papathanakos G, Arnaoutoglou E, Petrou A, Tzimas P. The effect of ondansetron on postoperative delirium and cognitive function in aged orthopedic patients. *Minerva Anestesiol.* 2014; **80**: 444-451.

90. Pretto G, Westphal GA, Silva E. Clonidine for reduction of hemodynamic and psychological effects of S+ ketamine anesthesia for dressing changes in patients with major burns: an RCT. *Burns.* 2014; **40**: 1300-1307.

91. Robinson TN, Dunn CL, Adams JC *et al.* Tryptophan supplementation and postoperative delirium--a randomized controlled trial. *J Am Geriatr Soc.* 2014; **62**: 1764-1771.

92. Sauer AM, Slooter AJ, Veldhuijzen DS, van Eijk MM, Devlin JW, van Dijk D. Intraoperative dexamethasone and delirium after cardiac surgery: a randomized clinical trial. *Anesth Analg.* 2014; **119**: 1046-1052.

93. Wang W, Li HL, Wang DX *et al.* Haloperidol prophylaxis decreases delirium incidence in elderly patients after noncardiac surgery: a randomized controlled trial*. *Crit Care Med.* 2012; **40**: 731-739.

94. Zaslavsky A, Haile M, Kline R, Iospa A, Frempong-Boadu A, Bekker A. Rivastigmine in the treatment of postoperative delirium: a pilot clinical trial. *Int J Geriatr Psychiatry.* 2012; **27**: 986-988.

95. Al-Aama T, Brymer C, Gutmanis I, Woolmore-Goodwin SM, Esbaugh J, Dasgupta M. Melatonin decreases delirium in elderly patients: a randomized, placebo-controlled trial. *Int J Geriatr Psychiatry.* 2011; **26**: 687-694.

96. Wan LJ, Huang QQ, Yue JX, Lin L, Li SH. [Comparison of sedative effect of dexmedetomidine and midazolam for post-operative patients undergoing mechanical ventilation in surgical intensive care unit]. *Zhongguo Wei Zhong Bing Ji Jiu Yi Xue.* 2011; **23**: 543-546.

97. Larsen KA, Kelly SE, Stern TA *et al.* Administration of olanzapine to prevent postoperative delirium in elderly joint-replacement patients: a randomized, controlled trial. *Psychosomatics.* 2010; **51**: 409-418.

98. Rubino AS, Onorati F, Caroleo S *et al.* Impact of clonidine administration on delirium and related respiratory weaning after surgical correction of acute type-A aortic dissection: results of a pilot study. *Interact Cardiovasc Thorac Surg.* 2010; **10**: 58-62.

99. Gamberini M, Bolliger D, Lurati Buse GA *et al.* Rivastigmine for the prevention of postoperative delirium in elderly patients undergoing elective cardiac surgery--a randomized controlled trial. *Crit Care Med.* 2009; **37**: 1762-1768.

100. Hudetz JA, Patterson KM, Iqbal Z *et al.* Ketamine attenuates delirium after cardiac surgery with cardiopulmonary bypass. *Journal of cardiothoracic and vascular anesthesia.* 2009; **23**: 651-657.

101. Maldonado JR, Wysong A, van der Starre PJ, Block T, Miller C, Reitz BA. Dexmedetomidine and the reduction of postoperative delirium after cardiac surgery. *Psychosomatics.* 2009; **50**: 206-217.

102. Prakanrattana U, Prapaitrakool S. Efficacy of risperidone for prevention of postoperative delirium in cardiac surgery. *Anaesthesia and intensive care.* 2007; **35**: 714-719.

103. Sampson EL, Raven PR, Ndhlovu PN *et al.* A randomized, double-blind, placebo-controlled trial of donepezil hydrochloride (Aricept) for reducing the incidence of postoperative delirium after elective total hip replacement. *Int J Geriatr Psychiatry.* 2007; **22**: 343-349.

104. Leung JM, Sands LP, Rico M *et al.* Pilot clinical trial of gabapentin to decrease postoperative delirium in older patients. *Neurology.* 2006; **67**: 1251-1253.

105. Kalisvaart KJ, de Jonghe JF, Bogaards MJ *et al.* Haloperidol prophylaxis for elderly hip-surgery patients at risk for delirium: a randomized placebo-controlled study. *J Am Geriatr Soc.* 2005; **53**: 1658-1666.

106. Liptzin B, Laki A, Garb JL, Fingeroth R, Krushell R. Donepezil in the prevention and treatment of post-surgical delirium. *Am J Geriatr Psychiatry.* 2005; **13**: 1100-1106.
